# Integrated sRNAome and RNA-Seq analysis reveals miRNA effects on betalain biosynthesis in pitaya

**DOI:** 10.1186/s12870-020-02622-x

**Published:** 2020-09-22

**Authors:** Canbin Chen, Fangfang Xie, Qingzhu Hua, Noemi Tel Zur, Lulu Zhang, Zhike Zhang, Rong Zhang, Jietang Zhao, Guibing Hu, Yonghua Qin

**Affiliations:** 1 State Key Laboratory for Conservation and Utilization of Subtropical Agrobioresources/Guangdong Provincial Key Laboratory of Postharvest Science of Fruits and Vegetables/Key Laboratory of Biology and Genetic Improvement of Horticultural Crops (South China), Ministry of Agriculture and Rural Affairs, College of Horticulture, South China Agricultural University, Guangzhou, Guangdong 510642 P. R. China; 2 French Associates Institute for Agriculture and Biotechnology of Drylands, The J. Blaustein Institutes for Desert Research, Ben-Gurion University of the Negev, Sede Boqer Campus, 84990 Beersheba, Israel

**Keywords:** *Hylocereus*, Betalain biosynthesis, sRNAome and RNA-Seq, miRNA, Gene expression, 5′RACE

## Abstract

**Background:**

MicroRNAs (miRNAs) and their regulatory functions in anthocyanin, carotenoid, and chlorophyll accumulation have been extensively characterized in many plant species. However, the miRNA regulatory mechanism in betalain biosynthesis remains mostly unknown.

**Results:**

In this study, 126 conserved miRNAs and 41 novel miRNAs were first isolated from *Hylocereus monacanthus*, among which 95 conserved miRNAs belonged to 53 miRNA families. Thirty-four candidate miRNAs related to betalain biosynthesis were differentially expressed. The expression patterns of those differential expressed miRNAs were analyzed in various pitaya tissues by RT-qPCR. A significantly negative correlation was detected between the expression levels of half those miRNAs and corresponding target genes. Target genes of miRNAs i.e. Hmo-miR157b-*HmSPL6-like*, Hmo-miR160a-*Hpcyt P450-like3*, Hmo-miR6020-*HmCYP71A8-like*, Hmo-novel-2-*HmCYP83B1-like*, Hmo-novel-15-*HmTPST-like*, Hmo-miR828a-*HmTT2-like*, Hmo-miR858-*HmMYB12-like*, Hmo-miR858-*HmMYBC1-like* and Hmo-miR858-*HmMYB2-like* were verified by 5′RACE and transient expression system in tobacco.

**Conclusions:**

Hmo-miR157b, Hmo-miR160a, Hmo-miR6020 Hmo-novel-2, Hmo-novel-15, Hmo-miR828a and Hmo-miR858 play important roles in pitaya fruit coloration and betalain accumulation. Our findings provide new insights into the roles of miRNAs and their target genes of regulatory functions involved in betalain biosynthesis of pitaya.

## Background

Mature microRNAs (miRNAs) are a type of endogenous non-coding small RNAs with 20–24 nucleotide (nt) length. miRNAs regulate their target genes by mRNA at the post-transcriptional level via the RNA-induced silencing complex (RISC) by binding with the Argonaute (AGO) protein to cleavage target mRNA or repress translation of target mRNA [1]. miRNAs play vital roles in plant growth and development, (a)biotic stress response, post-transcriptional regulation, and pigment regulation [2–4].

miRNAs are involved in chlorophyll, carotenoid, and anthocyanin biosynthesis. miRNAs can regulate coloration and chlorophyll accumulation [3, 5–7]. Overexpression of osa-miR171b by an artificial miRNA could enhance chlorophyll accumulation in rice leaves [7]. Based on the reduction of chlorophyll concentration under severe drought stress, a miRNA regulatory network consisting of two up-regulated miRNAs and nineteen down-regulated miRNAs was constructed in *Camellia sinensis* [3]. miRNAs are involved in regulating carotenoid pathways [8–12]. miR1857 affected carotenogenesis in a sweet orange red-flesh mutant and its wild type [8]. In different rose cultivars, miRNAs may negatively regulate target genes to prevent carotenoid accumulation resulting in white flowers according to expression analyses of five miRNAs [9]. miRNAs can also regulate anthocyanin accumulation through their target genes [13–25]. Anthocyanin accumulation is promoted with increasing of miR156 abundance in *Arabidopsis* [13] and litchi [20]. However, the reduction of miR156 abundance could lead to the accumulation of flavonols [13]. miR156 positively regulates anthocyanin accumulation by targeting the *SPL* transcription factors (TFs) [23]. miR828 negatively regulates anthocyanin accumulation by inhibiting the expression of *MYB75*, *MYB90*, and *MYB113* in *Arabidopsis* [14]. Moreover, miR858 and its targets involved in anthocyanin accumulation have been identified from apple, cotton, and tomato [15, 17, 18].

Pitaya, also known as pitahaya or dragon fruit, is a perennial climbing fruit crop belonging to the genus *Hylocereus* (Cactaceae) under the order Caryophyllales. As a member of the Cactaceae, pitaya exhibits a range of specific adaptations to arid lands in term of succulent stems with spines instead of leaves, the crassulacean acid metabolism (CAM) pathway [26–28]. It is an excellent plant material for basic and applied biological research. The potential economic impact of pitaya lies in its diverse uses not only as agricultural produce and processed foods but also in industrial and medicinal products. Pitaya is a fast-return fruit crop with production in the second year after planting and full production in 3–4 years. Pitaya fruit is mature in 28–50 days (28–35 days in summer and 35–50 days in autumn) after flowering and has 7–12 separate fruiting cycles per year due to climatic or nutritional limitations in South China. Therefore, pitaya has become a favorite fruit of many farmers and home gardeners in Southeast Asia, China, the United States, Israel, Australia, Cyprus and the Canary Islands.

The color of pitaya is attributed to the presence of betalains [29–32]. Betalains are red and yellow alkaloid pigments that are found in all families of the Caryophyllales with the exception of Molluginaceae and Caryophyllaceae which produce anthocyanins [33]. Betalains in pitaya fruit are not only good for human health but also can help consumers distinguish cultivars [29, 34, 35]. Betalains also play vital roles in the protection against drought, UV radiation, high saline soils, and diseases [35–39]. Pitaya is the only at large-scale commercially grown fruit containing abundant betalains for the consumer. Previous studies are mainly focused on characterizations of key genes and TFs involved in betalain biosynthesis. Key genes such as *tyrosinase* (*TYR*), *cytochrome P450* (*Cyt P450*), *4,5-dihydroxy-phenylalanine* (*DOPA*)*-dioxygenase* (*DOD*) and *glucosyltransferases* (*GTs*) [40] and TFs such as *WRKY* and *MYB* involved in betalain biosynthesis have been investigated in detail [41–43]. However, the roles of miRNAs in betalain biosynthesis has not been reported yet. In this study, candidate miRNAs and their target genes related to betalain biosynthesis were identified based on small RNA and transcriptome databases of pitaya pulp at different developmental stages. The aim of the present study is to explore the roles of miRNA in pitaya betalain biosynthesis, which may contribute to a better understanding of betalain biosynthesis in *Hylocereus*.

## Results

### Sequencing of sRNAs and the transcriptome

Six sRNA libraries were generated to identify miRNAs using pulps from ‘Guanhuahong’ pitaya on the 19^th^ day after flowering (DAF) (white pulp stage, Hp19d_1, and Hp19d_2), 25^th^ DAF (pulp coloration stages, Hp25d_1, and Hp25d_2) and 29^th^ DAF (mature stage, Hp29d_1, and Hp29d_2). The Illumina sequencing data of sRNAs from the 19^th^, 25^th^ and 29^th^ DAF showed that 24 nt sRNAs are the most abundant, followed by 21 nt sRNAs (Figure S1). A total of 82,318,241 reads were obtained from the sRNA datasets. After removal of the adaptor, insert, polyA and short RNAs of *<* 18 nt in length, 62,729,725 (76.20%) valid reads were obtained, including the rRNA, tRNA, snRNA, snoRNA and some other Rfam RNA (Table S1).

Three transcriptome libraries were constructed to identify the target genes of miRNAs using pulps from ‘Guanhuahong’ pitaya on the 19^th^ DAF (white pulp stage, Hp19d), 25^th^ DAF (pulp coloration stages, Hp25d) and 29^th^ DAF (mature stage, Hp29d). All screened reads were de novo assembled into 68,505 transcripts with an N50 of 1700 bp. And then, these transcripts were assembled into 39,737 unique sequences with an average length of 879 bp. The length distributions of those transcripts and unigenes were shown in Figure S2. The distribution of assembled transcripts and genes with different GC contents in the transcriptome datasets were summarized in Figure S3. The majority of transcripts and genes were in the range of 35–50% in GC contents.

### Identification of miRNAs

A total of 126 conserved miRNAs were identified based on BLAST searches and sequence analyses in comparison of sRNA sequences with known mature plant miRNAs in miRBase (Table S2). Among them, 95 known miRNAs belonging to 53 families were obtained. The number of miRNA members for each family varied from 1 (for MIR535) to 7 (for MIR482) (Figure S4). Novel miRNAs were predicted using the sRNA valid reads based on structure and expression criteria [44]. Forty-one novel candidate miRNAs with a clear precursor including stem-loop secondary structure were identified (Table S2). The length of novel miRNAs was between 20 and 25 nt. The most abundant sequences (49%) were 21 nt-length, and followed by 24 nt-length (29%), which was consistent with typical length distribution of mature miRNAs. The higher expression levels of the miRNA, the more copies of the miRNAs were sequenced. The abundance of known miRNAs was higher than that of putative novel miRNAs, except Hmo-novel-2, Hmo-novel-6, Hmo-novel-21 and Hmo-novel-22, which possessed more than 100 normalized reads (Table S2).

### Analyses of differentially expressed miRNAs

High-throughput sequencing (HTS) was performed to explore the expression changes of miRNAs involved in betalain biosynthesis of ‘Guanhuahong’ pitaya. Only miRNAs with expression values at *p* < 0.05 were considered to be significantly regulated. In Hp29d/Hp25d/Hp19d, 33 known miRNAs and five novel miRNAs were found to be differentially expressed (Table 1).

**Table 1:** The information of differentially expressed miRNAs in pitaya

miRNA names	miRNA sequences	Pvalue (ANOVA)	Hp19d (norm)	Hp25d (norm)	Hp29d (norm)
Hmo-MIR2916-p5	TACCGTCCTAGTCTCAACCATA	2.99E-07	807	141	2298
Hmo-miR171c	TTGAGCCGCGCCAATATCACC	1.97E-05	156	72	39
Hmo-MIR2916-p3	CAGGGATCGGCGGATGTTGCT	1.38E-04	2332	738	3765
Hmo-miR408	TTGCACTGCCTCTTCCCTGGC	5.14E-04	682	103	68
Hmo-miR396b	CGGTTCAATAAAGCTGTGGGA	8.78E-04	193	334	332
Hmo-miR393	TCCAAAGGGATCGCATTGATCC	1.98E-03	697	560	405
Hmo-miR164b	CATGTGCCTGTCTTCCCCATC	2.20E-03	1852	7112	24,055
Hmo-miR398c	TGTGTTCTCAGGTCGCCCCTG	2.58E-03	650	136	66
Hmo-miR390a	CGCTATCCATCCTGAGTTTCA	2.75E-03	187	57	85
Hmo-miR168a	GATCCCGCCTTGCATCAATTGAAT	3.04E-03	193	139	102
Hmo-miR529b	AGAAGAGGGAGAGTACAGCT	3.81E-03	1970	853	572
Hmo-miR398b	TGTGTTCTCAGGTCGCCCC	4.13E-03	89	11	7
Hmo-miR164a	TGGAGAAGCAGGGCACGTGCA	4.28E-03	4479	27,331	89,762
Hmo-novel-7	TTACTTGGCACTTACGACAGA	4.32E-03	29	63	59
Hmo-miR399a	CGCCAAAGGAGAGTTGCCCTT	4.40E-03	4	10	18
Hmo-miR8175	GTTCGATCCCCGGCAACGGCGCCA	4.40E-03	33	7	4
Hmo-miR156	TTGCCAGAAGAGAGTGAGCAC	6.04E-03	30	65	75
Hmo-miR6149a	TTGATACGCACCTGAATCGGC	6.08E-03	38	7	10
Hmo-miR172a	CGAATCTTGATGATGCTGCAT	7.33E-03	461	369	227
Hmo-miR397b	TTGAGTGCAGCGTTGATGAAAT	7.48E-03	31	5	1
Hmo-miR535	TGACAACGAGAGAGAGCACGC	8.93E-03	3907	10,306	9059
Hmo-miR159b	GGCTTGGATTGAAGGGAGCTCC	1.17E-02	20	7	9
Hmo-miR159a	TTTGGATTGAAGGGAGCTCTA	1.20E-02	198	182	108
Hmo-miR530	TGCATTTGCACCTGCACCTGA	1.71E-02	15	5	8
Hmo-miR6300	GTCGTTGTAGTATAGTGG	1.80E-02	2638	88	161
Hmo-novel-21	ACGCCTAATGCTGTGTATGGGAGG	1.90E-02	179	222	118
Hmo-miR390b	AAGCTCAGGAGGGATAGCGCC	1.91E-02	29	5	6
Hmo-miR398a	TGTGTTCTCAGGTCACCCCTT	1.93E-02	64	27	25
Hmo-novel-2	CAGCTTTCTTGAACTTTCCCC	2.23E-02	566	501	773
Hmo-miR394	TGGGCATTCTGTCCACCTCC	2.38E-02	6	2	2
Hmo-miR157b	CTGCCAGAAGATAGAGAGCAC	2.38E-02	50	105	148
Hmo-miR6020	AAATGTTCTTCGAGTATCTTC	2.51E-02	5	0	1
Hmo-miR160b	TGCCTGGCTCCCTGTATGCCG	2.89E-02	164	189	76
Hmo-miR5072	TCCCCAGTGGAGTCGCCA	2.95E-02	21	2	3
Hmo-novel-15	TCGCGCCTCGGGACCCTTTGC	2.99E-02	48	31	32
Hmo-novel-12	AGAGAAAGCATAAGCAACTGT	3.28E-02	0	8	6
Hmo-miR171d	TTGAGCCGTGCCAATATCCCA	4.38E-02	9	2	3
Hmo-miR482f	GGTATTGGTGGGTTGGAAAGC	4.88E-02	2	0	0

Changes in expression levels of miRNAs during fruit development of pitaya possibly reflected their potential functional differences. In different pulp coloration stages of ‘Guanhuahong’ pitaya, the expression levels of Hmo-miR396b, Hmo-miR535, Hmo-novel-7 and Hmo-novel-12 at the red pulp stages (25^th^ and 29^th^ DAP) were significantly higher than that of the white pulp stage (19^th^ DAP) (Table 1). Expression levels of Hmo-miR156, Hmo-miR157b, Hmo-miR164a, Hmo-miR164b and Hmo-miR399a increased gradually from 19^th^ DAF to 29^th^ DAF in ‘Guanhuahong’ pitaya. Those results were in consistent with betalain accumulation pattern [45, 46]. However, the expression levels of Hmo-miR171c, Hmo-miR408, Hmo-miR393, Hmo-miR398c, Hmo-miR8175, Hmo-miR398a, Hmo-miR168a, Hmo-miR529b, Hmo-miR398b, Hmo-miR172a, Hmo-miR159a and Hmo-miR397b decreased gradually from 19^th^ DAF to 29^th^ DAF during fruit maturation of ‘Guanhuahong’ pitaya. Twelve differentially expressed miRNAs i.e. Hmo-miR390a, Hmo-miR6149a, Hmo-miR159b, Hmo-miR530, Hmo-miR6300, Hmo-miR390b, Hmo-miR6020, Hmo-miR394, Hmo-miR5072, Hmo-novel-15, Hmo-miR171d and Hmo-miR482f showed a lower expression level at the red pulp stages (25^th^ and 29^th^ DAP) compared to a relatively higher expression level at white pulp stage (19^th^ DAP). Those results were in contrast to betalain accumulation pattern suggesting that those miRNAs negatively regulated their target genes in betalain accumulation.

### Bioinformatics of transcriptome analyses

All 39,737 unique sequences were annotated according to BLAST (cut-off E-value ≤10^− 5^) searches of Nr, Swiss-Prot Protein, Pfam, Gene Ontology (GO), euKaryotic Ortholog Groups (KOG) and Kyoto Encyclopedia of Genes and Genomes (KEGG) database (Table S3).

**Fig. 1:**
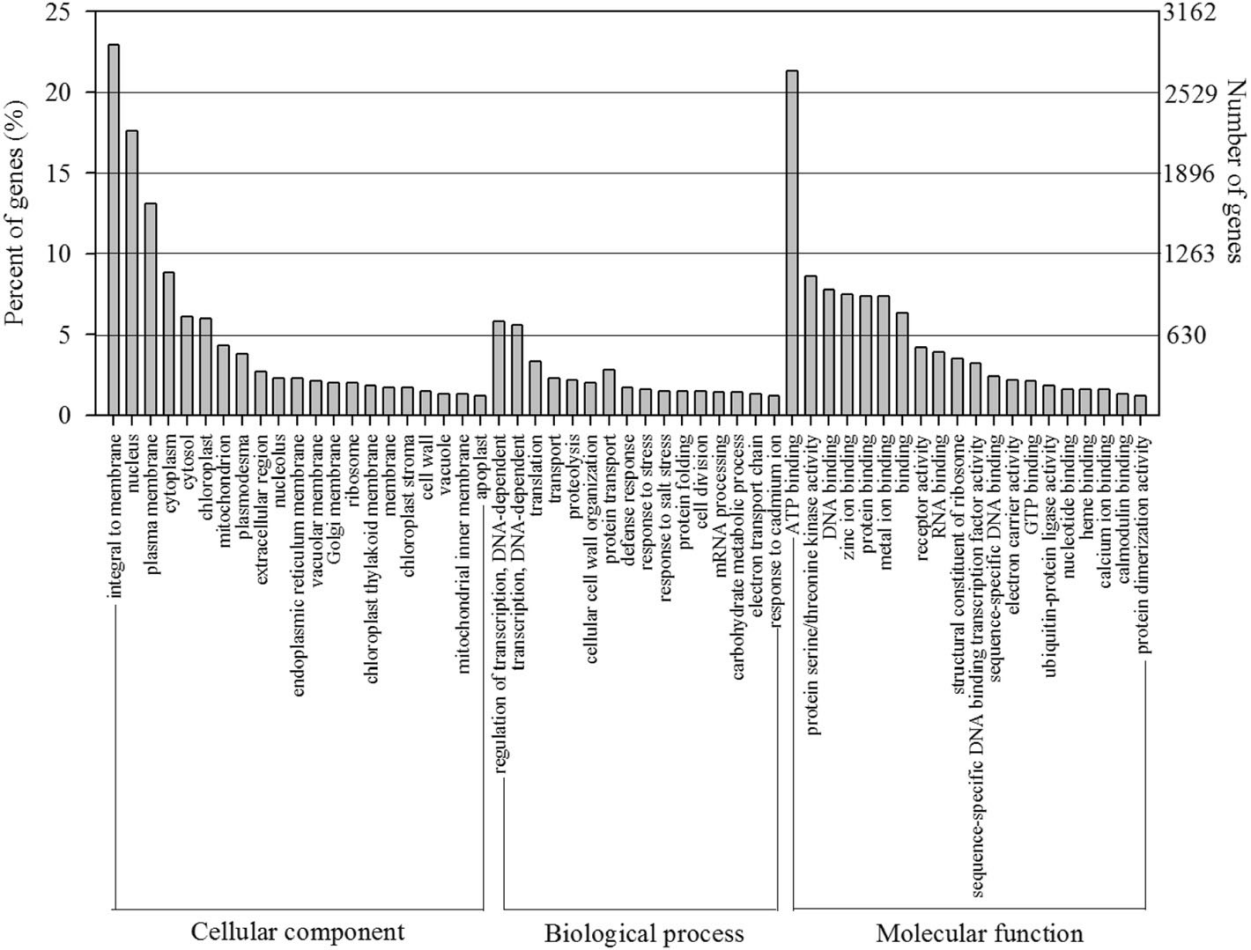
Histogram of GO classifications for transcripts of pitaya pulp

The GO, KEGG and KOG databases were used to classify the functions of the predicted unigenes. Twelve thousand five hundred fifty-nine unigenes were classified into three main categories: ‘biological process’, ‘cellular component’, and ‘molecular function’ by GO database (Fig. 1). As for the ‘molecular function’ category, the largest number of unigenes was gathered in ‘ATP binding’, while the major groups for the ‘cellular component’ category were ‘integral to membrane’ (2878 unigenes, 23%), ‘nucleus’ (2211 unigenes, 18%) and ‘plasma membrane’ (1646 unigenes, 13%). In the category of ‘biological process’, ‘regulation of transcription, DNA-dependent’ (729 unigenes, 5.8%) and ‘transcription, DNA-dependent’ (706 unigenes, 5.6%) were the two most abundant subcategories.

**Fig. 2:**
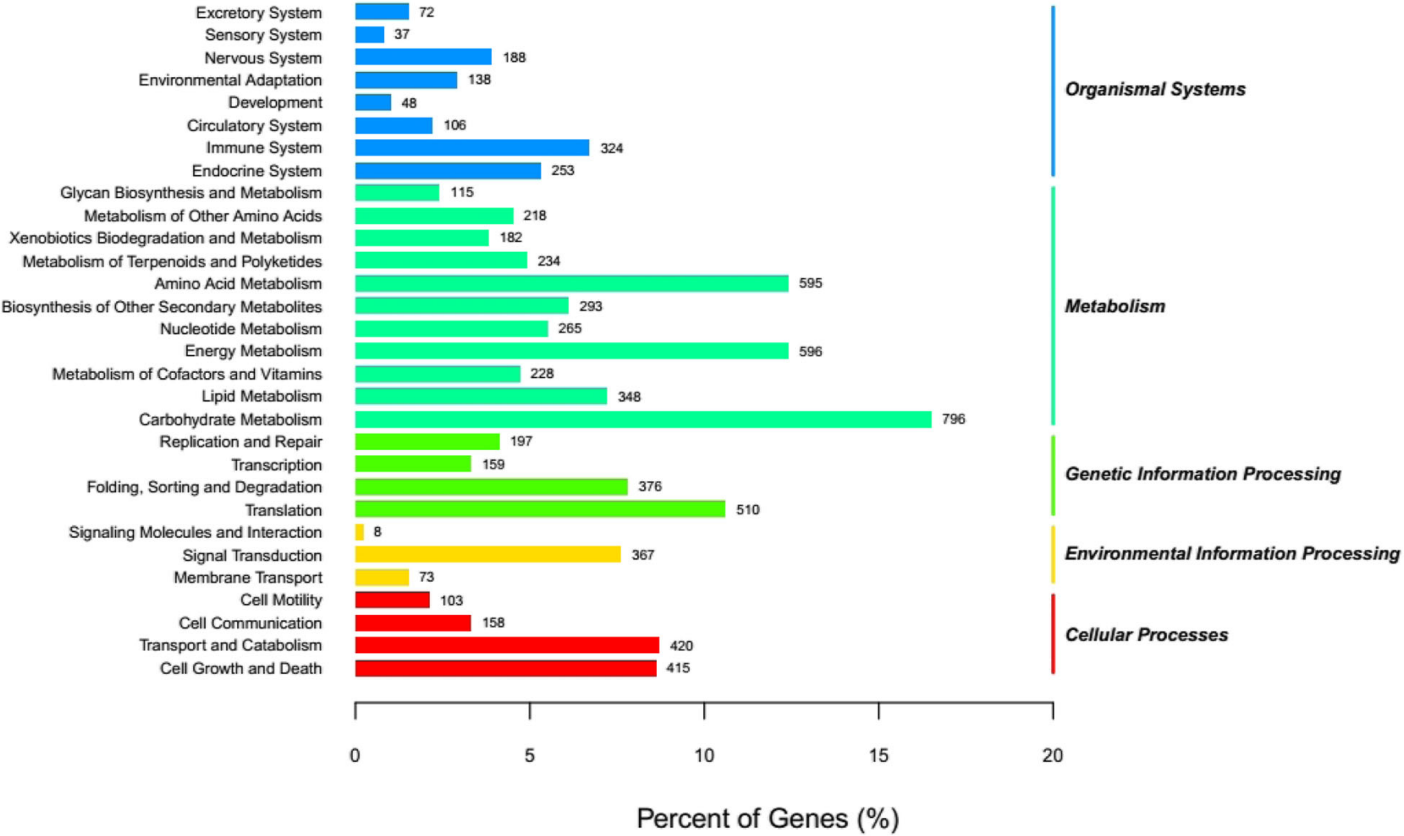
KEGG pathway classifications for transcripts of pitaya pulp

**Fig. 3:**
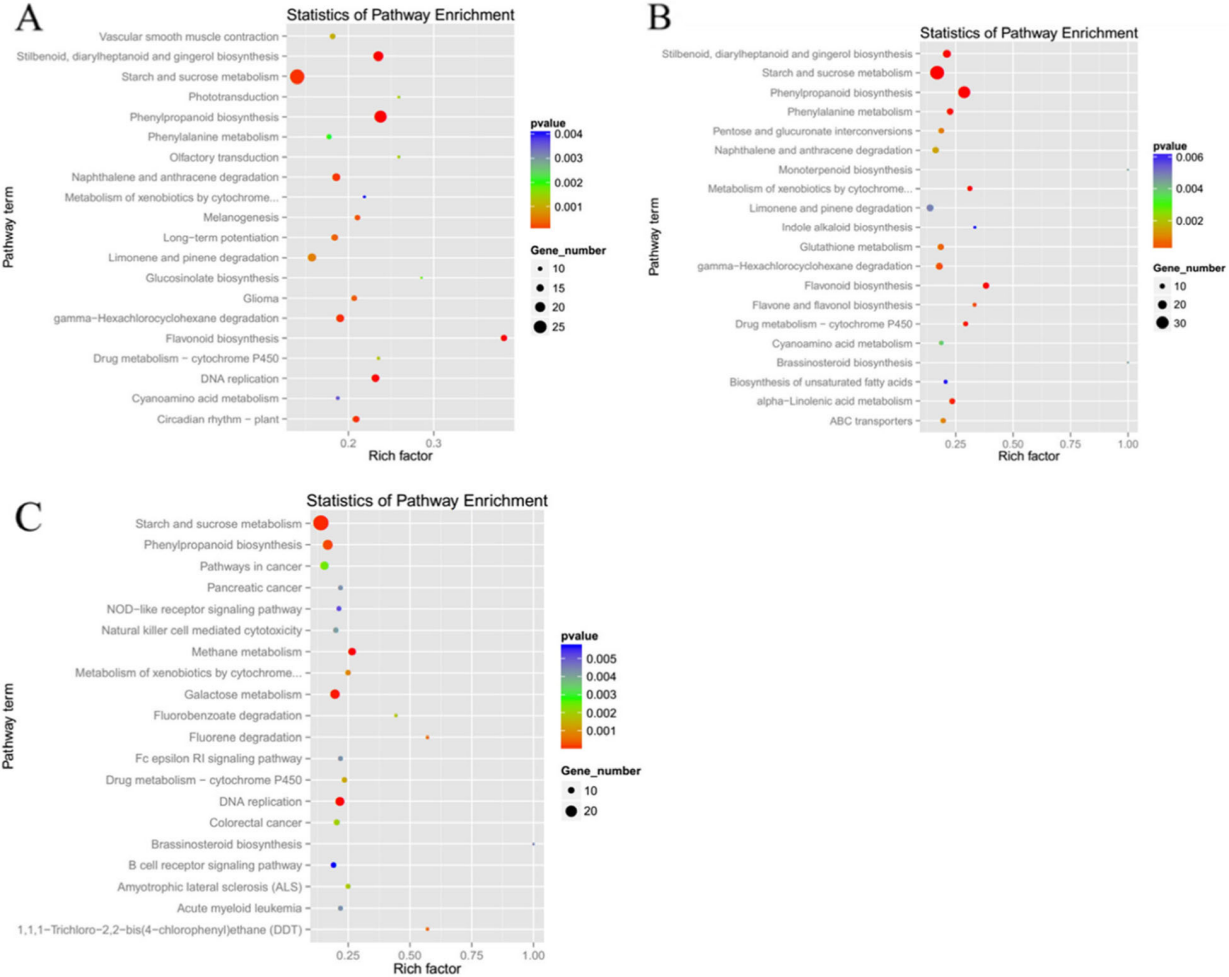
KEGG pathway enrichment for differential gene expression of the three coloration stages. **a** *Hp*25 d-VS-*Hp*19 d. **b** *Hp*29 d-VS-*Hp*19 d. **c** *Hp*29 d-VS-*Hp*25 d

Seven thousand eight hundred twenty-two unigenes were mapped onto KEGG pathways. The highest number of unigenes was carbohydrate metabolism (796 unigenes), followed by energy metabolism (596 unigenes), amino acid metabolism (595 unigenes) and translation (510 unigenes) (Fig. 2). Differential expression genes (DEGs) in the Hp25d-VS-Hp19d, Hp29d-VS-Hp19d, and Hp29d-VS-Hp25d were analyzed. In the Hp25d-VS-Hp19d, 24, 20, 16 and 13 DEGs were involved in phenylpropanoid, stilbenoid, diarylheptanoid and gingerol biosynthesis, DNA replication and flavonoid biosynthesis pathway (*p* < 0.0001) (Fig. 3a), respectively. In Hp29d-VS-Hp19d, 140 genes were found to be significantly differentially expressed (p < 0.0001), including biosynthesis of phenylpropanoid, flavonoid, stilbenoid, diarylheptanoid and gingerol, metabolism of phenylalanine, α-linolenic acid, starch and sucrose, xenobiotics by Cyt P450, as well as drug metabolism-Cyt P450 (Fig. 3b). The expression levels of DEGs among different groups in one pathway showed different scales of changes. Thirteen DEGs were detected from the flavonoid biosynthesis pathway in the Hp25d-VS-Hp19d and Hp29d-VS-Hp19d, respectively. However, no DEGs related to flavonoid biosynthesis were found in Hp29d-VS-Hp25d (Fig. 3c). Those results suggested that flavonoid biosynthesis is a crucial pathway in pitaya betalain biosynthesis.

**Fig. 4:**
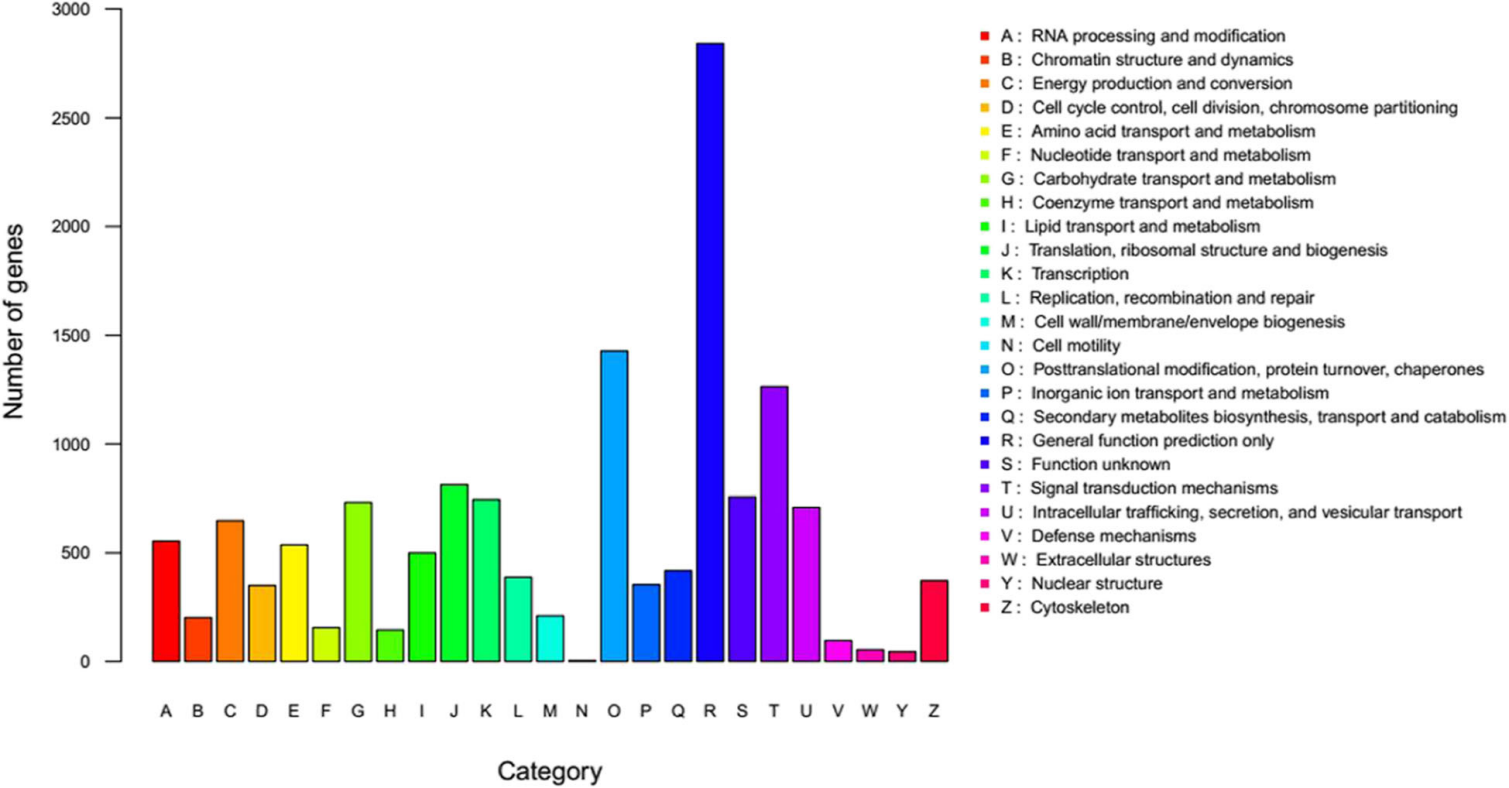
Histogram of KOG functional categories for transcripts of pitaya pulp

The KOG database is used to study the classification and evolutionary rates of orthologous proteins. As shown in Fig. 4, group R (general function prediction only), group O (posttranslational modification, protein turnover, chaperones) and group T (signal transduction mechanisms) are the three most abundant groups in pitaya dataset, suggesting that a large number of transcriptional and posttranslational regulation of gene expression and function are involved in pitaya fruit development.

**Fig. 5:**
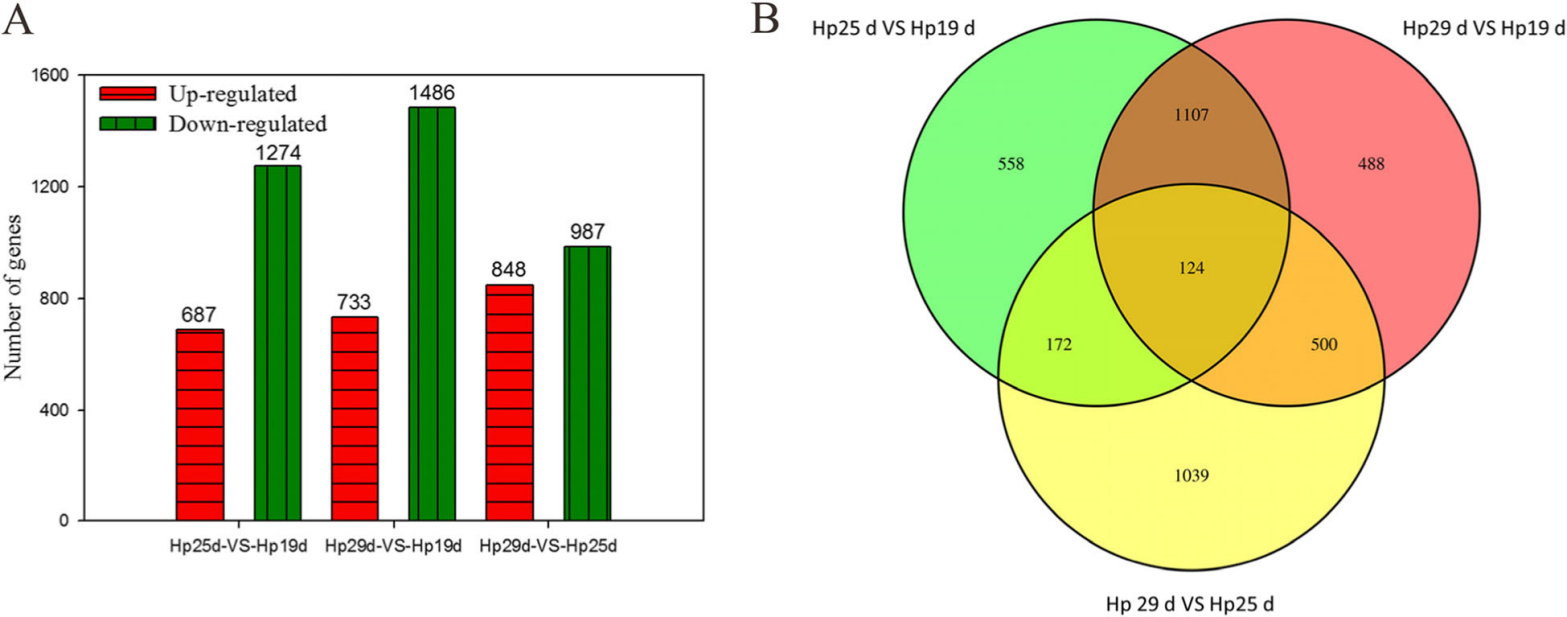
Differential gene expression profiles based on the libraries of the three coloration stages. **a** The numbers of up- and down-regulated genes in comparisons of the *Hp*25 d-VS-*Hp*19 d, *Hp*29 d-VS-*Hp*19 d and *Hp*29 d-VS-*Hp*25 d. **b** The comparison of DEGs between any two stages of the pitaya pulp

DEGs from three development stages (Hp25d-VS-Hp19d, Hp29d-VS-Hp19d, and Hp29d-VS-Hp25d) were evaluated by pairwise comparisons using the expression fold (|log_2_fold change| ≥ 1) and *p* values< 0.05 as the thresholds (Fig. 5). In the pairwise comparisons between any two stages, 3988 genes were found to be significantly differentially expressed. The highest amount of DEGs was obtained between the Hp19d and Hp29d libraries, including 1486 down-regulated and 733 up-regulated (Fig. 5a). The lowest number of DEGs (1835) was detected between the Hp25d and Hp29d libraries (987 down-regulated and 848 up-regulated), followed by the Hp19d and Hp25d libraries (1961 DEGs including 1274 down-regulated and 687 up-regulated). Among those DEGs, 124 genes were significantly differentially expressed in all three fruit development stages of ‘Guanhuahong’ pitaya (Fig. 5b).

### Verification of the accuracy of the sRNAome and RNA-Seq data

**Fig. 6:**
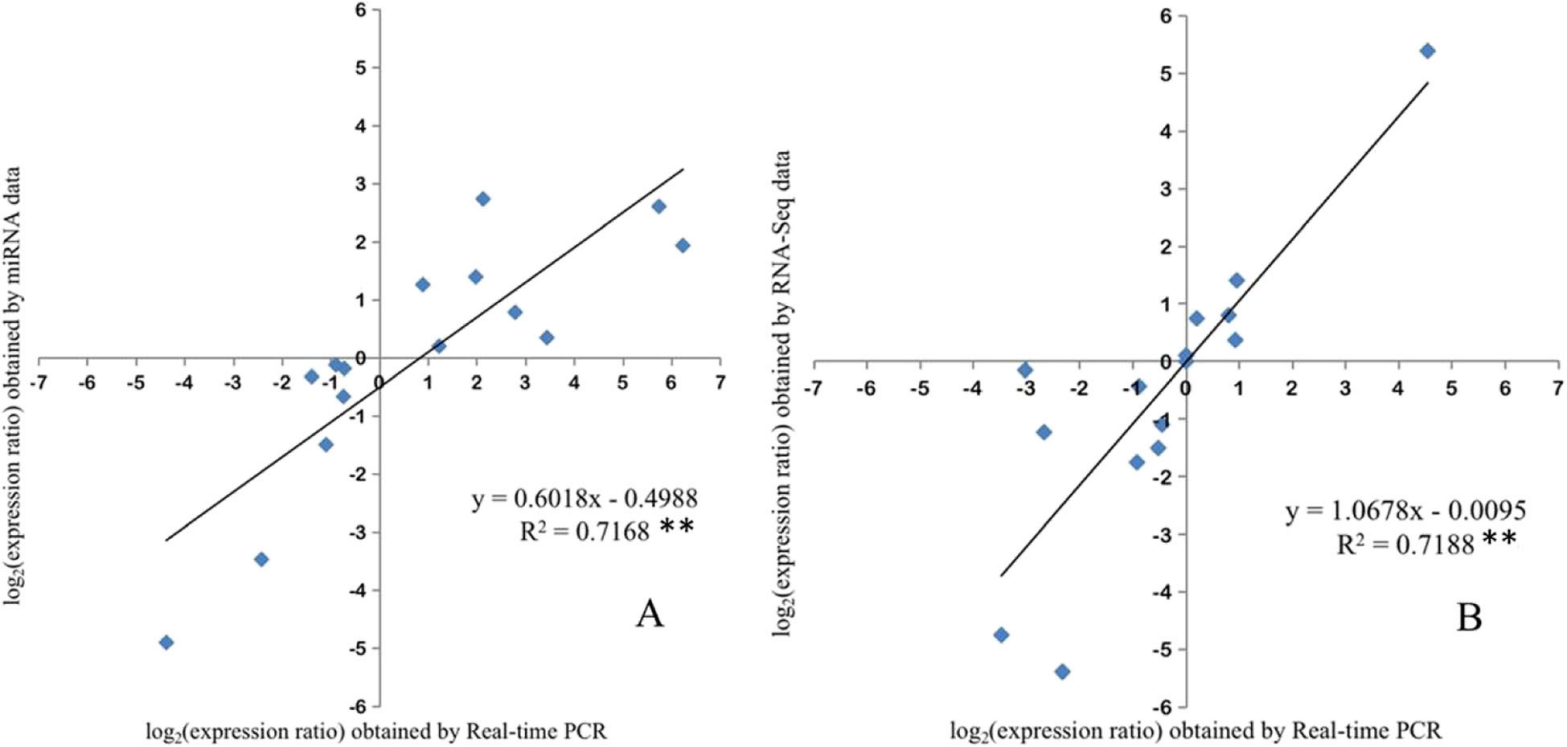
Verification of the accuracy of the sRNAome and RNA-Seq data by coefficient analyses. **a** Coefficient analyses of miRNA expression levels from sRNAome and RT-qPCR data. **b** Coefficient analyses of gene expression levels from RNA-Seq and RT-qPCR data. The RT-qPCR log_2_ values (x-axis) were plotted against high-throughput data (y-axis). **indicates a significant difference at p<0.01 (*n* = 15)

To verify the reliability of the sRNAome and RNA-Seq results, the expression of fifteen miRNAs and fifteen genes related to betalain biosynthesis were analyzed by RT-qPCR. The IDs, Reads Per Kilobase of exon model per Million mapped read (RPKM) value, and primers for the 30 transcripts were shown in Tables S2, S5, S6, and S8, respectively. The overall correlation coefficients of 0.717** and 0.719** were obtained by linear regression [(Q-PCR value) = a (sRNAome or RNA-Seq value) + b] analysis (Fig. 6), respectively, indicating that the results of sRNAome and transcriptome were consistent with those of RT-qPCR. Those results suggested that sRNAome and RNA-Seq data can be used for subsequent experiments.

### Prediction of targets for differentially expressed miRNAs

To investigate the functions of miRNAs in betalain biosynthesis of pitaya, it is crucial to predict their target genes. Target genes of differentially-expressed miRNAs were predicted by Target Finder software. Transcripts of transcriptome were identified as possible target genes for the majority of miRNAs. A total of 124 target mRNAs, 22 target mRNAs for 25 conserved miRNAs and 5 novel miRNAs were obtained, respectively (Table S9). More than one target gene was predicted for most miRNAs. miRNAs have the potential to regulate targets belonging to certain gene families with different biological functions. For example, the predicted target genes of Hmo-miR529b were found to be involved in squamosa promoter-binding-like protein 6 (SPL6), histone-lysine N-methyltransferase and ubiquitin-protein ligase. Based on transcriptome annotation, six targets involved in betalain biosynthesis were selected [23, 40–42]. Among them, *HmCYP83B1-like*, *HmCYP71A8-like*, *HmTPST-like*, *HmWDTC1-like* and *HmSPL16-like* were respectively targeted by Hmo-novel-2, Hmo-miR6020, Hmo-novel-15, Hmo-miR164a and Hmo-miR156 while *HmSPL6-like* was co-targeted by Hmo-miR156, Hmo-miR157b and Hmo-miR529b.

### Tissue-specific analyses of differentially expressed miRNAs

**Fig. 7:**
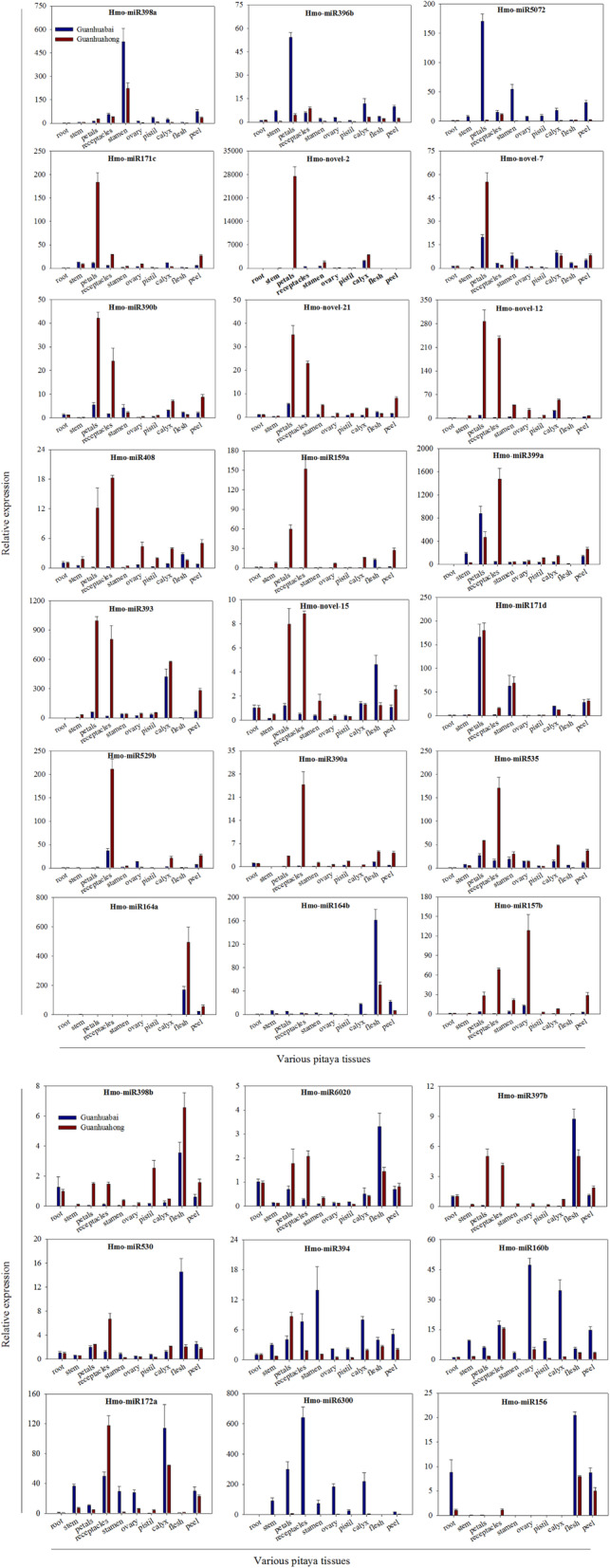
RT-qPCR analyses of miRNAs in various pitaya tissues using the expression of the *U6* gene as the reference

miRNA preferentially expressed in specific tissues can provide clues to its physiological functions. Thirty differentially expressed miRNAs were analyzed their functions by RT-qPCR using ten different tissues from ‘Guanhuabai’ and ‘Guanhuahong’ pitayas. As shown in Fig. 7, the 30 differentially expressed miRNAs showed different expression levels in those pitaya tissues. Hmo-miR172a, Hmo-miR394, Hmo-miR530, Hmo-miR6020, Hmo-miR397b, Hmo-miR160b and Hmo-miR398b displayed similar expression patterns. Hmo-miR156 preferentially expressed in pitaya roots and fruits. Hmo-miR398a strongly expressed in pitaya stamens compared to weak expression in the other tissues. Higher expression levels of Hmo-novel-7 and Hmo-miR171d were detected in petals but moderately or weakly expressed in the other tissues. Hmo-miR164a and Hmo-miR164b displayed constitutive expression and the highest expression level was detected in pitaya fruits. Hmo-miR396b and Hmo-miR5072 had higher expression in petals of ‘Guanhuabai’ pitaya compared to higher expression of Hmo-miR171c and Hmo-novel-2 in petals of ‘Guanhuahong’ pitaya. Hmo-novel-12, Hmo-novel-21, Hmo-novel-15, Hmo-miR393, Hmo-miR390b, Hmo-miR159a and Hmo-miR408 were preferentially expressed in petals and receptacles of ‘Guanhuahong’ pitaya. Hmo-miR157b and Hmo-miR6300 were highly expressed in ‘Guanhuahong’ and ‘Guanhuabai’ pitayas, respectively. Results from RT-qPCR indicated that expression of the 30 miRNAs from pitaya had tissue- and/or cultivar- characteristics.

### Validation of miRNAs and target genes related to betalain biosynthesis

**Fig. 8:**
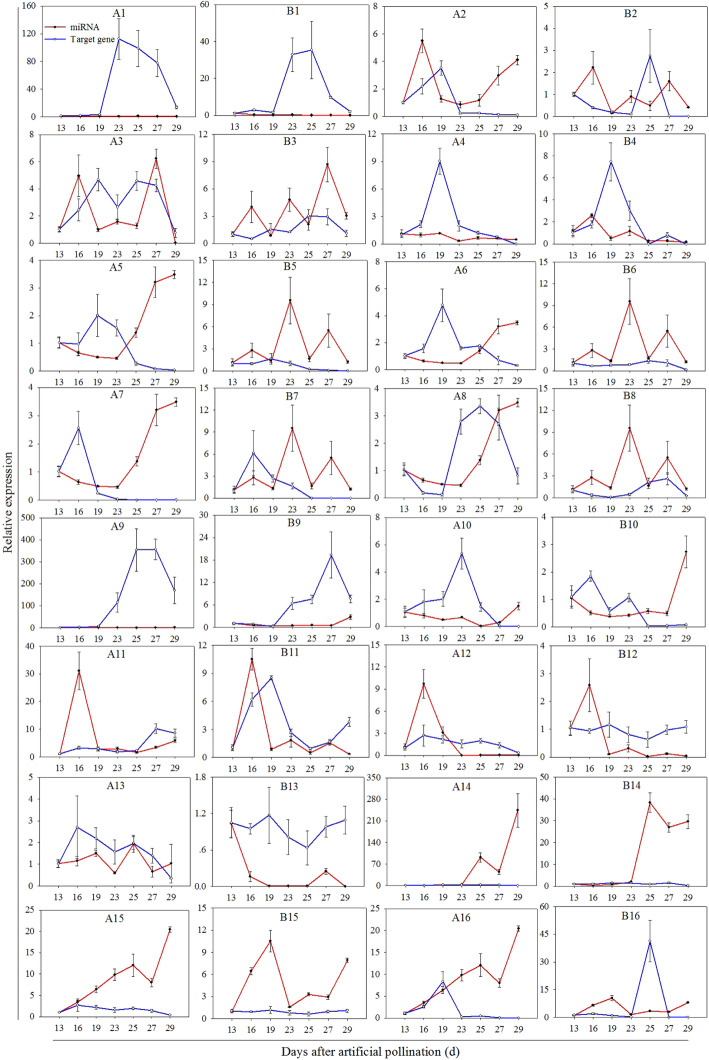
Expression analyses of miRNAs and their target genes by RT-qPCR. (A1-A16) ‘Guanhuabai’ pitaya. (**B1-B16**) ‘Guanhuahong’ pitaya. (**A1-B1**) Hmo-miR828a and *HmTT2-like*. (**A2-B2**) Hmo-miR6020 and *HmCYP71A8-like*. (**A3-B3**) Hmo-novel-2 and *HmCYP83B1-like*. (**A4-B4**) Hmo-miR159c and *HmGmSGT2-like*. (**A5-B5**) Hmo-miR858 and *HmMYB12-like*. (**A6-B6**) Hmo-miR858 and *HmMYBC1-like*. (**A7-B7**) Hmo-miR858 and *HmMYB2-like*. (**A8-B8**) Hmo-miR858 and *HmMYB315-like*. (**A9-B9**) Hmo-miR160a and *Hpcyt P450-like3*. (**A10-B10**) Hmo-miR160a and *Hpcyt P450-like2*. (**A11-B11**) Hmo-novel-15 and *HmTPST-like*. (**A12-B12**) Hmo-miR157b and *HmSPL6-like*. (**A13-B13**) Hmo-miR529b and *HmSPL6-like*. (**A14-B14**) Hmo-miR164a and *HmWDTC1-like*. (**A15-B15**) Hmo-miR156 and *HmSPL6-like*. (**A16-B16**) Hmo-miR156 and *HmSPL16-like*

miRNAs involved in pitaya betalain biosynthesis were screened based on annotated transcripts and previous reports related to pigment synthesis. Fourteen target genes predicted from 11 miRNAs were assayed by RT-qPCR. The target genes from the ‘Guanhuahong’ and ‘Guanhuabai’ pitayas had the same sequences (Figure S5). Sixteen targets showed decreased or increased expression trends along with the increased or decreased expression of the miRNA, suggesting that they might be actively cleaved by miRNAs (Fig. 8). For example, *Hpcyt P450-like3* (Fig. 8A9 and B9) and *Hpcyt P450-like2* (Fig. 8A10 and B10) targeted by Hmo-miR160a, *HmMYB12-like* (Fig. 8A5), *HmMYBC1-like* (Fig. 8A6) and *HmMYB2-like* (Fig. 8A7) targeted by Hmo-miR858, and *HmCYP71A8-like* (Fig. 8A2) targeted by Hmo-miR6020 showed increasing expression trend at first and decreasing thereafter while Hmo-miR160a, Hmo-miR858 and Hmo-miR6020 showed a reverse trend at all pulp coloration stages of pitaya. *HmSPL6-like* (Fig. 8A15) targeted by Hmo-miR156 showed decreasing while Hmo-miR156 showed increasing at all pulp coloration stages of pitaya. However, some targeted genes and their miRNAs such as *HmTPST-like* (Fig. 8B11) targeted by Hmo-novel-15 in ‘Guanhuahong’ pitaya and *HmSPL6-like* (Fig. 8A13) targeted by Hmo-miR529b in ‘Guanhuabai’ pitaya had similar expression patterns at all pulp stages of pitaya.

**Fig. 9:**
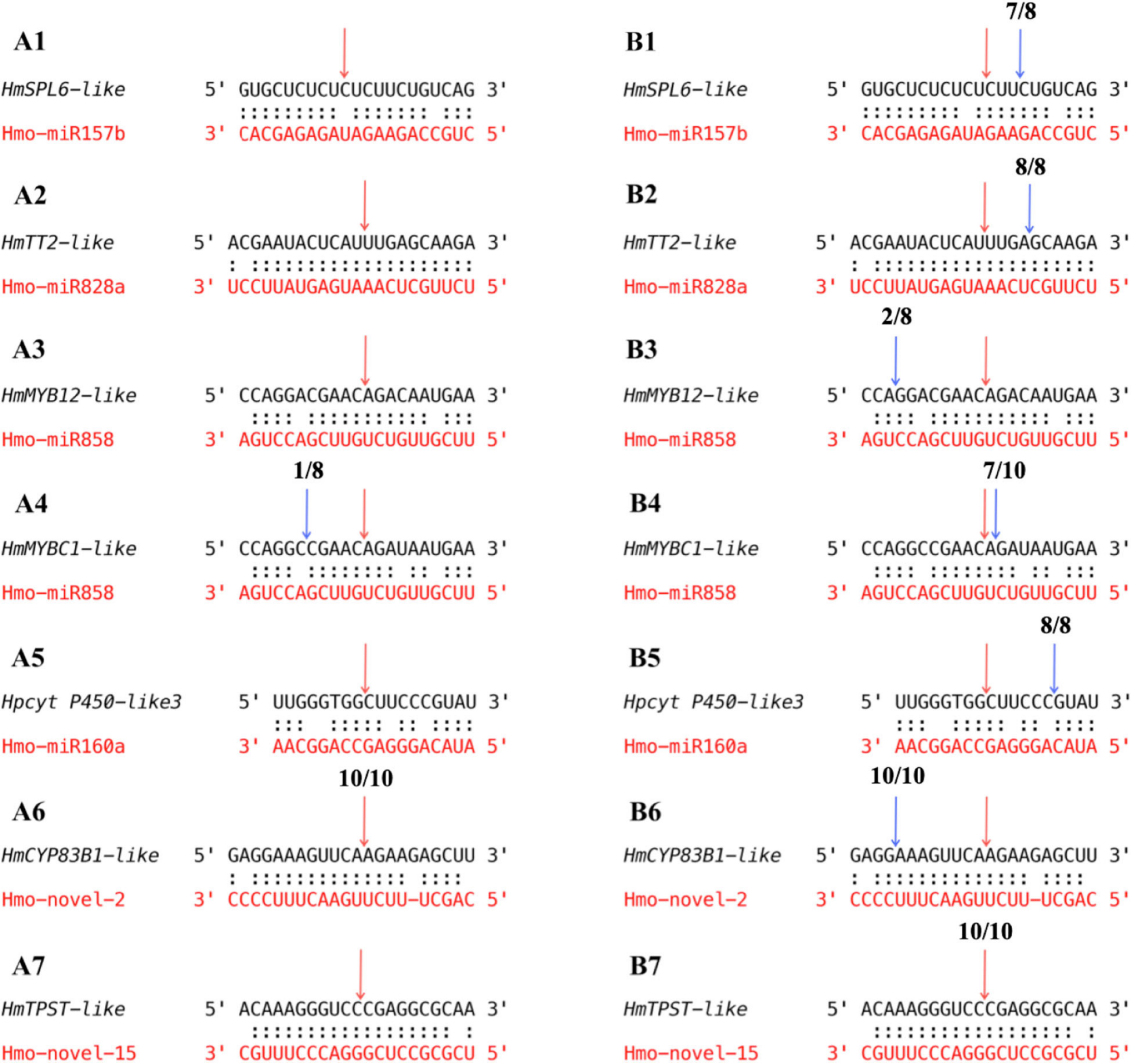
RLM-5′-RACE validation of miRNA-mediated cleavage of target genes. (**A1-A7**) ‘Guanhuabai’ pitaya. (**B1-B7**) ‘Guanhuahong’ pitaya. Top strand (black) depicts a miRNA complementary site, and bottom strand (red) depicts a miRNA complementary site the miRNA. Watson–Crick pairing (:) are indicated. Blue arrows indicate the cleavage sites of targets and the numbers show the frequency of the clones sequenced. Red arrows indicate the cleavage site of miRNA on target gene occurring at the 10^th^ nucleotide from the 5′-end of miRNA in the binding region and the numbers show the frequency of the clones sequenced. The cleavage sites outside of the displayed sequence are not shown

5′RACE analyses were further verified the fourteen candidate targets. The *HmSPL6-like*, *HmTT2-like*, *HmMYB12-like*, *HmMYBC1-like*, *Hpcyt P450-like3*, *HmCYP83B1-like* and *HmTPST-like* were confirmed to be cleaved by their corresponding miRNAs (Fig. 9). The cleavage sites of Hmo-novel-2 on *HmCYP83B1-like* and Hmo-novel-15 on *HmTPST-like* were both occurred at the 10^th^ nucleotide from the 5′-end of miRNAs in the binding region. The cleavage frequency of Hmo-novel-2 on *HmCYP83B1-like* and Hmo-novel-15 on *HmTPST-like* was up to 10/10 both in ‘Guanhuabai’ and ‘Guanhuahong’ pitayas, respectively. Those results confirmed that Hmo-novel-2 and Hmo-novel-15 can guide the cleavage of the mRNA of *HmCYP83B1-like* and *HmTPST-like*, respectively. Cleavage occurred mostly at the 10^th^ nucleotide from the 5′-end in the binding sites. However, *HmMYBC1-like* occurred at the 9^th^ nucleotide in ‘Guanhuahong’ pitaya, which may be due to the wide range of miRNA cutting mRNA caused by siRNA interference. Besides, the same gene from ‘Guanhuahong’ pitaya is different from ‘Guanhuabai’ pitaya (Fig. 9), which may be responsible for the different pulp colors of the two pitaya cultivars.

### The relationship between miRNAs and their target genes

**Fig. 10:**
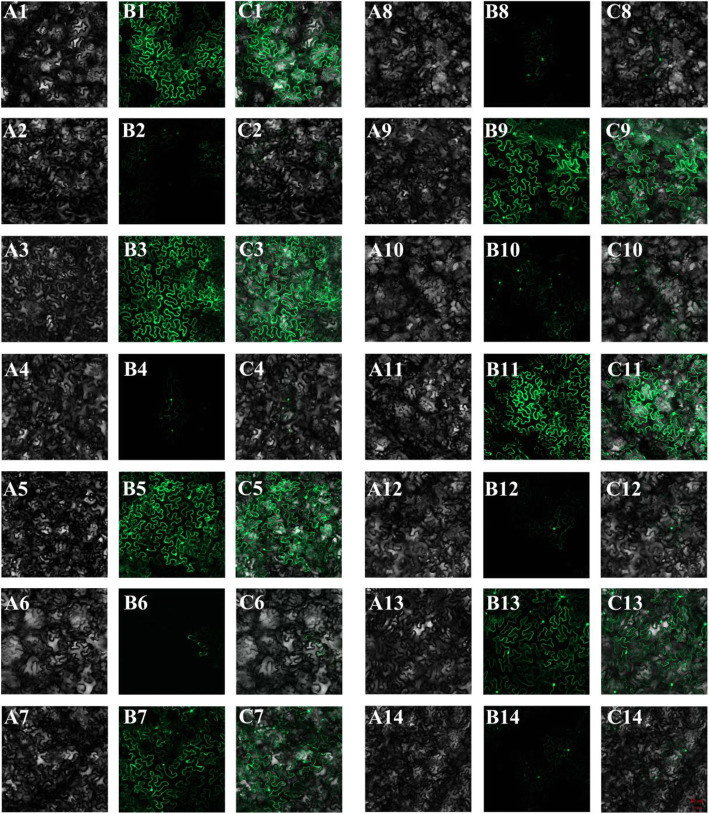
Transient expressions of miRNAs and their target genes (part). (**A1-A14**) bright field. (**B1-B14**) eGFP. (**C1-C14**) merge. (**A1-C1**) 35 s::*HmCYP71A8-like*::eGFP. (**A2-C2**) 35 s::*HmCYP71A8-like*::eGFP+ 35 s::Pre-Hmo-miR6020. (**A3-C3**) 35 s::*HmMYB12-like*::eGFP. (**A4-C4**) 35 s::*HmMYB12-like*::eGFP+ 35 s::Pre-Hmo-miR858. (**A5-C5**) 35 s::*HmMYBC1-like*::eGFP. (**A6-C6**) 35 s::*HmMYBC1-like*::eGFP+ 35 s::Pre-Hmo-miR858. (**A7-C7**) 35 s::*HmMYB2-like*::eGFP. (**A8-C8**) 35 s::*HmMYB2-like*::eGFP+ 35 s::Pre-Hmo-miR858. (**A9-C9**) 35 s::*Hpcyt P450-like2*::eGFP. (**A10-C10**) 35 s::*Hpcyt P450-like2*::eGFP+ 35 s::Pre-Hmo-miR160a. (**A11-C11**) 35 s::*HmCYP83B1-like*::eGFP. (**A12-C12**) 35 s::*HmCYP83B1-like*::eGFP+ 35 s::Pre-Hmo-novel-2. (**A13-C13**) 35 s::*HmTPST-like*::eGFP. (**A14-C14**) 35 s::*HmTPST-like*::eGFP+ 35 s::Pre-Hmo-novel-15. All transient expressions (including negative and positive controls) are presented in Figure S6

The transient expression system was constructed to confirm that miRNAs degrade their target genes in vivo. Target sites of miRNAs in target genes and a modified target site (inactivated target site) were inserted into the over-expression vector harboring an enhanced green fluorescent protein (*eGFP*) gene, respectively. A total of five miRNAs and seven corresponding target genes i.e. Hmo-miR160a-*Hpcyt P450-like2*, Hmo-miR6020-*HmCYP71A8-like*, Hmo-novel-2-*HmCYP83B1-like*, Hmo-novel-15-*HmTPST-like*, Hmo-miR858-*HmMYB12-like*, Hmo-miR858-*HmMYBC1-like* and Hmo-miR858-*HmMYB2-like* were verified their interactions using a tobacco transient expression system (Figure S6). Co-expression of Hmo-miR160a-*Hpcyt P450-like2*, Hmo-miR6020-*HmCYP71A8-like*, Hmo-novel-2-*HmCYP83B1-like*, Hmo-novel-15-*HmTPST-like*, Hmo-miR858-*HmMYB12-like*, Hmo-miR858-*HmMYBC1-like* and Hmo-miR858-*HmMYB2-like* inhibited the expression of *eGFP*, indicating that miRNAs degrade their target genes in vivo (Fig. 10B2, 4, 6, 8, 10, 12 and 14). The result was consistent with the positive control (Figure S6B10, 18, 28, 32, 40 and 48). The interactions between miRNAs and target genes could affect miRNA processing resulting in higher precursor accumulation and reduced mature miRNA in pitaya, and further regulate betalain biosynthesis in pitaya (Fig. 10).

## Discussion

Great progress has been made in pitaya betalain studies in term of physical and chemical properties [47, 48], purification and identification [49–54], antioxidant and radical scavenging capacity [55–57] as well as metabolic and transcriptional analyses [45, 46]. However, no information is available about miRNAs involved in betalain biosynthesis of pitaya. Therefore, identification of pitaya miRNAs associated with their target genes will help our understanding of molecular regulatory mechanisms of pitaya betalain biosynthesis. In this study, transcriptome and sRNAome were performed to explore the role of miRNAs in pitaya coloration mechanisms at different developmental stages. The percentage of 24 nt sRNAs (an average of 58.03%) was much higher than that of 21 nt sRNAs (an average of 12.16%) in pitaya pulp (Figure S1). The result was consistent with the length distribution of sRNAs in apple and litchi [15, 20, 58], but was inconsistent with the findings in strawberry, orange, *Brassica juncea*, and apple [21, 59–61] suggesting that different plant species have different length sRNA distribution. A total of 95 known miRNAs belonging to 53 miRNA families and 41 new miRNAs were identified from ‘Guanhuahong’ pitaya (*H. monacanthus)* (Table S2). miRNAs showed different expression levels during fruit developmental stages of pitaya, indicating that miRNAs play essential roles in pitaya fruit development. Those results suggested that miRNAs are involved in pitaya growth and development through different expressions in various pitaya tissues.

Generally, conserved miRNAs have the same or homologous targets as other plant species, and most of them show a similar function. MIR156 plays a key role in the biosynthesis of secondary metabolites. MIR156 can positively regulate anthocyanin biosynthesis by SPL TFs, while SPL TFs negatively regulate anthocyanin accumulation in *Arabidopsis*, apple, litchi, and *Pyrus pyrifolia* [13, 20, 21, 23]. Bgy-mir156 regulates the target gene *phytoene synthase* (*PSY*) and affects the accumulation of carotenoids in carrot (*Daucus carota*) [12]. Betalains are secondary metabolites but cannot co-exist naturally in one plant at the same time [62]. Pitaya is a high-value, functional fruit containing abundant betalains. In our study, the highest expression level of Hmo-miR157b was detected on the 23^rd^ DAF (color conversion) in ‘Guanhuahong’ pitaya, suggesting that Hmo-miR157b may play significant roles in betalain accumulation. Results from 5′RACE showed that *HmSPL6-like* was targeted by Hmo-miR157b (Fig. 7). Those results indicated that Hmo-miR157b might positively regulate betalain biosynthesis by *SPL* TFs, while *SPL* TFs negatively regulates betalain accumulation in pitaya.

MiR828 participated in anthocyanin biosynthesis by repressing the expression of *MYB* TFs in *Arabidopsis*, apple and potato [14, 15, 21, 24]. In this study, the expression levels of *HmTT2-like* after 23^rd^ DAF of ‘Guanhuahong’ pitaya were lower than that of the ‘Guanhuabai’ pitaya (Fig. 8A1 and B1). *HmTT2-like* showed a negative correlation with betalain accumulation in pitaya [45]. Hmo-miR828a was highly active on the 23^rd^ DAF of ‘Guanhuahong’ pitaya (color conversion). Hmo-miR828a could target *HmTT2-like* (Fig. 9B2), a *MYB* TF, suggesting that Hmo-miR828a positively regulate betalain accumulation in pitaya. miR858 could directly or indirectly control anthocyanin biosynthesis in *Arabidopsis*, cotton, apple and tomato, and negatively regulate anthocyanin accumulation by the *MYB* TF [14, 15, 17, 18]. In the present study, four target genes i.e., *HmMYB315-like*, *HmMYB12-like*, *HmMYBC1-like* and *HmMYB2-like* shared the same negative expression pattern with Hmo-miR858 in ‘Guanhuabai’ pitaya (Fig. 8A5-A8, and B5-B8). 5′RACE and transient expression analyses showed that Hmo-miR858 targeted *HmMYB12-like*, *HmMYBC1-like*, and *HmMYB2-like* in ‘Guanhuahong’ pitaya (Fig. 9B3-B4 and Fig. 10A3-A8, B3-B8 and C3-C8). Those results suggested that Hmo-miR858 can promote pitaya betalain accumulation by *MYB* genes.

*Hpcyt P450-like3* is involved in betalain biosynthesis in *H. monacanthus* [45]. In this study, *Hpcyt P450-like3* was targeted by Hmo-miR160a, suggesting that Hmo-miR160a was involved in betalain biosynthesis (Fig. 9B5). Novel miRNAs are involved in accumulations of chlorophyll, carotenoid, and anthocyanin. Ttu-novel-48 could regulate chlorophyll accumulation in leaves of durum wheat [6]. Csi-novel-03 regulates carotenoid pathways by *AP2* TFs in *Citrus* [8]. In strawberry receptacle fruit ripening, ABA-induced Fa_novel23 resulted in the rapid accumulation of fruit anthocyanin [25]. In our study, two novel miRNAs: Hmo-novel-2 and Hmo-novel-15 were obtained. Hmo-novel-2 influenced betalain accumulation via the regulation of *HmCYP83B1-like* (Fig. 9A6-B6 and Fig. 10A11-C11 and A12-C12). Hmo-novel-15 was identified as a regulator of betalain biosynthesis regulating the expression of *HmTPST-like* (Figs. 9B7 and 10A13-C13 and A14-C14). Hmo-miR6020-*HmCYP71A8-like* was not verified in 5′RACE but in the tobacco transient expression system (Fig. 10A1-C1 and A2-C2) suggesting that the translation inhibited the regulation modes of miRNAs on their targets rather than the degradation of mRNAs. The rest of the differentially expressed miRNAs and their targets in HTS were not confirmed in 5′RACE and tobacco transient expression system, indicating that these genes might not be targets of these miRNAs. Further work is necessary to elucidate their roles in betalain biosynthesis of pitaya.

## Conclusions

In this study, sRNAome and RNA-Seq were first used to identify differentially expressed miRNAs and their target genes involved in betalain biosynthesis. Comprehensive sRNAome analyses uncovered 126 conserved miRNAs and 41 novel miRNAs were obtained from ‘Guanhuahong’ pitaya (*H. monacanthus)*, among which 95 conserved miRNAs belonged to 53 miRNA families. 26.79 Gb raw RNA-Seq data were generated and de novo assembled into 68,505 transcripts, in which 39,737 were annotated. miRNAs and their target genes involved in betalain accumulation were compared at different developmental stages of pitaya fruit. Seven target genes were verified by 5′RACE and a tobacco transient expression system. Those Hmo-miRNAs negatively regulated expression of their target mRNAs through guiding corresponding target mRNA cleavage or inhibiting the translation. Hmo-miR157b-*HmSPL6-like*, Hmo-miR160a-*Hpcyt P450-like3*, Hmo-miR6020-*HmCYP71A8-like*, Hmo-novel-2-*HmCYP83B1-like*, Hmo-novel-15-*HmTPST-like*, Hmo-miR828a-*HmTT2-like*, Hmo-miR858-*HmMYB12-like*, Hmo-miR858-*HmMYBC1-like* and Hmo-miR858-*HmMYB2-like* are possibly involved in betalain biosynthesis in pitaya. The present study provides new information that miRNAs are actively involved in betalain accumulation of pitaya fruit by regulating the upstream TFs, which may contribute to a further understanding of miRNAs in betalain biosynthesis of pitaya.

## Methods

### Plant materials

Two pitaya cultivars, i.e., ‘Guanhuahong’ (red peel with red pulp, *H. monacanthus*) and ‘Guanhuabai’ (red peel with white pulp, *H. undatus*) and *Nicotiana benthamiana* were used as plant materials. ‘Guanhuahong’ and ‘Guanhuabai’ pitayas, authenticated by Professor Guibing Hu and Yonghua Qin (College of Horticulture, South China Agricultural University), were selected from 860 seedlings of ‘Hongshuijing’ (*H. monacanthus*) [63–65]. *Nicotiana benthamiana* was grown in a greenhouse with a condition of 16 h/8 h day/night at 25 °C and was used for interactions between miRNAs and their target gene assays in vivo. The South China Agricultural University provided all plant materials used in this study, and no specific permissions were required for the collection of those samples for research purposes following institutional, national and international guidelines. Fruits of ‘Guanhuahong’ and ‘Guanhuabai’ pitayas from the same orchard of Dalingshan Forest Park were separated into peels and pulps on the 13^th^, 16^th^, 19^th^, 23^rd^, 25^th^, 27^th^ and 29^th^ DAF (Figure S7) for expression analyses of crucial miRNAs and their targets. Pulps from ‘Guanhuahong’ pitaya on the 19^th^ DAF (white pulp stage, Hp19d, Hp19d_1, and Hp19d_2), 25^th^ DAF (pulp coloration stages, Hp25d, Hp25d_1, and Hp25d_2) and 29^th^ DAF (mature stage, Hp29d, Hp29d_1, and Hp29d_2) were used for RNA-Seq and sRNAome. All samples were frozen immediately in liquid nitrogen and stored at − 80 °C until use.

### SRNAome and RNA-Seq

Total RNA was extracted using the TruSeq Small RNA Sample Prep Kits (Illumina, San Diego, USA) according to the manufacturer’s instructions. RNA-Seq and sRNAome libraries were performed according to the procedures of Han et al. (2016) and Liu et al. (2016), respectively [20, 66]. Six small RNA libraries (Hp19d_1, Hp19d_2, Hp25d_1, Hp25d_2, Hp29d_1 and Hp29d_2) from two biological replicates and three RNA-Seq libraries (Hp19d, Hp25d and Hp29d) were constructed (https://dataview.ncbi.nlm.nih.gov/object/PRJNA588519? reviewer=ko91rr55muepqo1d25plnp0kv4). All HTS was performed by LC-BIO (Hangzhou, China).

### Bioinformatic analyses

Clean sRNA sequences were obtained from sRNAome raw data (raw reads) by removing adapters, low-quality tags, and contaminants. Clean sRNA sequences were compared with the Rfam database (http://rfam.sanger.ac.uk/) after removing rRNA, tRNA, snRNA, and snoRNA. To screen known miRNAs, the clean data were mapped to the reference sequence in miRBase21.0 by Bowtie [67]. miRNA precursor was submitted to RNAfold software (http://rna.tbi.univie.ac.at/cgi-bin/RNAfold.cgi) to identify novel miRNA [44].

Analyses of transcriptome raw data was identical to that of sRNAome raw data. Clean transcriptome data were assembled into non-redundant unigenes using Trinity (http://trinityrnaseq.github.io/). Unigenes were tentatively identified based on the best hits against known sequences in the database.

### Prediction and functional annotation of target gene

Target genes of differentially-expressed miRNAs were predicted by Target Finder software. GO, KOG and KEGG were used to analyze the functions of target genes. *E*-value ≤10^− 5^ was considered as significant enrichment.

### Sequence alignment analyses

Multiple sequences were aligned using DNAMAN software (version 8). The miRNA target sites were identified using sequence alignments and manual analyses.

### Analyses of miRNAs and target genes by RT-qPCR

Stem-loop RT-qPCR was used to confirm expression of miRNAs since it is a highly sensitive method for detection of miRNAs [68]. cDNAs were produced from 1.0 μg of total RNA samples using the MMLV-reverse transcriptase (Invitrogen) with miRNA specific stem-loop and oligo(dT) primers, respectively. The specific primers and PCR reactions were performed according to our previous method [20]. RT-qPCR was conducted in ABI 7500 real-time PCR System (Applied Biosystems, CA, USA) using the SYBR qPCR Mix (Vazyme). Twenty microliters reaction mixture contained 2.0 μL of diluted cDNAs (~ 15 ng/μL), 10.0 μL 2 × SYBR qPCR Mix (Vazyme), 0.5 μL of each primer (10 μM) and 7.0 μL ddH_2_O. All experiments were performed in triplicate. *U6* and *actin* gene were used as reference genes [69, 70]. The sequences of miRNAs and target genes were shown in Tables S2 and S10, respectively. All primers used for RT-qPCR analyses were listed in Tables S4, S5 and S6. The expression levels of miRNAs and target genes were calculated by 2^-△△C^_T_ method [71].

### 5′RACE analyses

To verify the miRNA-mediated cleavage events, RNA ligase-mediated 5′RACE (RLM-RACE) was performed using the SMARTer® RACE 5′/3′Kit User Manual (012615) (TaKaRa, Dalian, China) according to the manufacturer’s manual. 1.0 μg total RNA from the pulps of the 29^th^ DAF ‘Guanhuahong’ and ‘Guanhuabai’ pitayas was ligated with 5′RNA adapters, respectively. The ligated mRNA was reversely transcribed by oligo (dT) primer. 5′ end products were obtained using 5′ adaptor primers and 3′ gene-specific primers. PCR products were inserted into pMD18-T vector (TaKaRa). Specific primers used for nested PCR were shown in Table S7.

### Transient expression analysis

A transient expression system was used to confirm the interaction between miRNAs and their target genes in vivo. Construction of expression vectors were constructed following the procedure of Liu et al. [69]. Over-expression vectors of miRNAs related to the betalain biosynthesis and a control miRNA were constructed respectively. Co-expression of miRNAs and their targets in *N. benthamiana* leaves using *Agrobacterium tumefaciens* GV3101 infiltration. Transient expression in *N. benthamiana* was performed as described by Sparkes et al. (2006) [72]. Three days after infiltration, leaves were observed with a laser scanning confocal microscope (Zeiss LSM800) according to the following parameters: laser wavelength of 488 nm (laser intensity of 0.2%), master gain values ranging from 550 to 700 V, ESID gain value of 3 and digital gain value of 1.0. The transient expression assays were repeated at least three  times.

## Supplementary information

**Additional file 1: Figure S1.** The length distributions of miRNA fragments in pitaya (unique).

**Additional file 2: Figure S2.** The length distributions of assembled transcripts and genes. **A**, The length distributions of transcripts; **B**, The length distributions of genes.

**Additional file 3: Figure S3.** GC content distributions of assembled transcripts and genes. **A**, GC content distributions of transcripts; **B**, GC content distributions of genes.

**Additional file 4: Figure S4.** Number of miRNA family members in pitaya pulp.

**Additional file 5: Figure S5.** The sequences of 17 target genes from the two pitaya cultivars. ‘-W’, ‘Guanhuabai’ pitaya. ‘-R’, ‘Guanhuahong’ pitaya. Red lines indicate sequence of target genes primers for real-time PCR. Green lines indicate outer specific primers for nested PCR. Blue lines indicate inner specific primers for nested PCR.

**Additional file 6: Figure S6.** Transient expressions of miRNAs and their target genes (all). A1-A49, bright field; B1-B49, eGFP; C1-C49, merge; A1-C1, 35 s::eGFP; A2-C2, WT; A3-C3, 35 s::Pre-miR164; A4-C4, 35 s::Pre-Hmo-miR6020; A5-C5, 35 s::*HmCYP71A8-like*::eGFP; A6-C6, 35 s::positive control *HmCYP71A8-like*::eGFP; A7-C7, 35 s::negative control *HmCYP71A8-like*::eGFP; A8-C8, 35 s::*HmCYP71A8-like*::eGFP+ 35 s::Pre-miR164; A9-C9, 35 s::*HmCYP71A8-like*::eGFP+ 35 s::Pre-Hmo-miR6020; A10-C10, 35 s::positive control *HmCYP71A8-like*::eGFP+ 35 s::Pre-Hmo-miR6020; A11-C11, 35 s::negative control *HmCYP71A8-like*::eGFP+ 35 s::Pre-Hmo-miR6020; A12-C12, 35 s::Pre-Hmo-miR858; A13-C13, 35 s::*HmMYB12-like*::eGFP; A14-C14, 35 s::positive control *HmMYB12-like*::eGFP; A15-C15, 35 s::negative control *HmMYB12-like*::eGFP; A16-C16, 35 s::*HmMYB12-like*::eGFP+ 35 s::Pre-miR164; A17-C17, 35 s::*HmMYB12-like*::eGFP+ 35 s::Pre-Hmo-miR858; A18-C18, 35 s::positive control *HmMYB12-like*::eGFP+ 35 s::Pre-Hmo-miR858; A19-C19, 35 s::negative control *HmMYB12-like*::eGFP+ 35 s::Pre-Hmo-miR858; A20-C20, 35 s::*HmMYBC1-like*::eGFP; A21-C21, 35 s::*HmMYBC1-like*::eGFP+ 35 s::Pre-miR164; A22-C22, 35 s::*HmMYBC1-like*::eGFP+ 35 s::Pre-Hmo-miR858; A23-C23, 35 s::*HmMYB2-like*::eGFP; A24-C24, 35 s::*HmMYB2-like*::eGFP+ 35 s::Pre-miR164; A25-C25, 35 s::*HmMYB2-like*::eGFP+ 35 s::Pre-Hmo-miR858; A26-C26, 35 s::Pre-Hmo-miR160a; A27-C27, 35 s::*Hpcyt P450-like2*::eGFP; A28-C28, 35 s::positive control *Hpcyt P450-like2*::eGFP; A29-C29, 35 s::negative control *Hpcyt P450-like2*::eGFP; A30-C30, 35 s::*Hpcyt P450-like2*::eGFP+ 35 s::Pre-miR164; A31-C31, 35 s::*Hpcyt P450-like2*::eGFP+ 35 s::Pre-Hmo-miR160a; A32-C32, 35 s::positive control *Hpcyt P450-like2*::eGFP+ 35 s::Pre-Hmo-miR160a; A33-C33, 35 s::negative control *Hpcyt P450-like2*::eGFP+ 35 s::Pre-Hmo-miR160a; A34-C34, 35 s::Pre-Hmo-novel-2; A35-C35, 35 s::*HmCYP83B1-like*::eGFP; A36-C36, 35 s::positive control *HmCYP83B1-like*::eGFP; A37-C37, 35 s::negative control *HmCYP83B1-like*::eGFP; A38-C38, 35 s::*HmCYP83B1-like*::eGFP+ 35 s::Pre-miR164; A39-C39, 35 s::*HmCYP83B1-like*::eGFP+35 s::Pre-Hmo-novel-2; A40-C40, 35 s::positive control *HmCYP83B1-like*::eGFP+ 35 s::Pre-Hmo-novel-2; A41-C41, 35 s::negative control *HmCYP83B1-like*::eGFP+ 35 s::Pre-Hmo-novel-2; A42-C42, 35 s::Pre-Hmo-novel-15; A43-C43, 35 s::*HmTPST-like*::eGFP; A44-C44, 35 s::positive control *HmTPST-like*::eGFP; A45-C45, 35 s::negative control *HmTPST-like*::eGFP; A46-C46, 35 s::*HmTPST-like*::eGFP+ 35 s::Pre-miR164; A47-C47, 35 s::*HmTPST-like*::eGFP+ 35 s::Pre-Hmo-novel-15; A48-C48, 35 s::positive control *HmTPST-like*::eGFP+ 35 s::Pre-Hmo-novel-15; A49-C49, 35 s::negative control *HmTPST-like*::eGFP+ 35 s::Pre-Hmo-novel-15.

**Additional file 7: Figure S7.** Different fruit developmental stages of ‘Guanhuahong’ (A) and ‘Guanhuabai’ (B) pitayas. A1 and B1, 13 d; A2 and B2, 16 d; A3 and B3, 19 d; A4 and B4, 23 d; A5 and B5, 25 d; A6 and B6, 27 d; A7 and B7, 29 d. Bar = 2.0 cm.

**Additional file 8: Table S1.** Statistics of miRNA sequences in pitaya.

**Additional file 9: Table S2.** The information of miRNAs in pitaya.

**Additional file 10: Table S3.** Statistics of gene annotation in six databases.

**Additional file 11: Table S4.** Sequences of miRNA-specific primers for reverse transcription.

**Additional file 12: Table S5.** Sequences of miRNA primers for real-time PCR.

**Additional file 13: Table S6.** Sequences of target genes primers for real-time PCR.

**Additional file 14: Table S7.** Sequences of specific primers for 5′RACE.

**Additional file 15: Table S8.** The information of part RNA-Seq data in pitaya.

**Additional file 16: Table S9.** Prediction of targets for differentially expression miRNAs in pitaya.

**Additional file 17: Table S10.** cDNA sequences of 17 target genes.

## Data Availability

The transcriptome clean raw reads data that support the findings of this study have been submitted to NCBI Sequence Read Archive (SRA) under Accession (SAMN13253272- SAMN13253280), Bioproject: PRJNA588519. All data generated or analyzed during this study are included in this published article and its supplementary information files. The authors are pleased to share with the data upon request.

## Abbreviations

5′RACE: 5′ rapid amplification of cDNA ends
AGO: Argonaute
CAM: Crassulacean acid metabolism
cDNA: Complementary DNA
CYP: Cytochrome P450
Cyt P450: Cytochrome P450
DAF: Day after flowering
DEG: Differential expression gene
DOD: Dioxygenase
DOPA: 4,5-dihydroxy-phenylalanine
eGFP: Enhanced green fluorescent protein
GO: Gene Ontology
GTs: Glucosyltransferases
HTS: High-throughput sequencing
KEGG: Kyoto Encyclopedia of Genes and Genomes
KOG: euKaryotic Ortholog Groups
miRNA: microRNA
MYB: Myb proto-oncogene protein
nt: Nucleotide
PSY: Phytoene synthase
RISC: RNA-induced silencing complex
RNA-Seq: RNA sequencing
RPKM: Reads Per Kilobase of exon model per Million mapped read
RT-qPCR: Reverse transcription quantitative real-time polymerase chain reaction
SPL: Squamosa promoter-binding-like
TFs: Transcription factors
TPST: Protein-tyrosine sulfotransferase
TYR: Tyrosinase
WDTC1: WD and tetratricopeptide repeats protein 1
