# Correction: Integrated sRNAome and RNA-Seq analysis reveals miRNA effects on betalain biosynthesis in pitaya

**DOI:** 10.1186/s12870-023-04129-7

**Published:** 2023-02-23

**Authors:** Canbin Chen, Fangfang Xie, Qingzhu Hua, Noemi Tel-Zur, Lulu Zhang, Zhike Zhang, Rong Zhang, Jietang Zhao, Guibing Hu, Yonghua Qin

**Affiliations:** 1 State Key Laboratory for Conservation and Utilization of Subtropical Agrobioresources/Guangdong Provincial Key Laboratory of Postharvest Science of Fruits and Vegetables/Key Laboratory of Biology and Genetic Improvement of Horticultural Crops (South China), Ministry of Agriculture and Rural Affairs, College of Horticulture, South China Agricultural University, Guangzhou, Guangdong 510642 P. R. China; 2 French Associates Institute for Agriculture and Biotechnology of Drylands, The J. Blaustein Institutes for Desert Research, Ben-Gurion University of the Negev, Sede Boqer Campus, 84990 Beersheba, Israel

**Correction:**  ***BMC Plant Biol*** **20, 437 (2020)**

**https://doi.org/10.1186/s12870-020-02622-x**

Following publication of the original article [1], the authors identified an error in the author name of Noemi Tel-Zur. The family name was not captured correctly.

The incorrect family name is: Zur

The correct family name is: Tel-Zur

The author group has been updated above and the original article [1] has been corrected.
