# Supplementary material for: Integrated sRNAome and RNA-Seq analysis reveals miRNA effects on betalain biosynthesis in pitaya
Source: BMC Plant Biol. 2020 Sep 22;20:437. doi: 10.1186/s12870-020-02622-x (PMC7510087; doi:10.1186/s12870-020-02622-x)
Supplement: Supplementary file 1 — Additional file 1: Figure S1. The length distributions of miRNA fragments in pitaya (unique). [file 12870_2020_2622_MOESM1_ESM.docx]

**FIGURE S1 | The length distributions of miRNA fragments in pitaya (unique).**
