# Supplementary material for: Integrated sRNAome and RNA-Seq analysis reveals miRNA effects on betalain biosynthesis in pitaya
Source: BMC Plant Biol. 2020 Sep 22;20:437. doi: 10.1186/s12870-020-02622-x (PMC7510087; doi:10.1186/s12870-020-02622-x)
Supplement: Supplementary file 2 — Additional file 2: Figure S2. The length distributions of assembled transcripts and genes. A, The length distributions of transcripts; B, The length distributions of genes. [file 12870_2020_2622_MOESM2_ESM.docx]

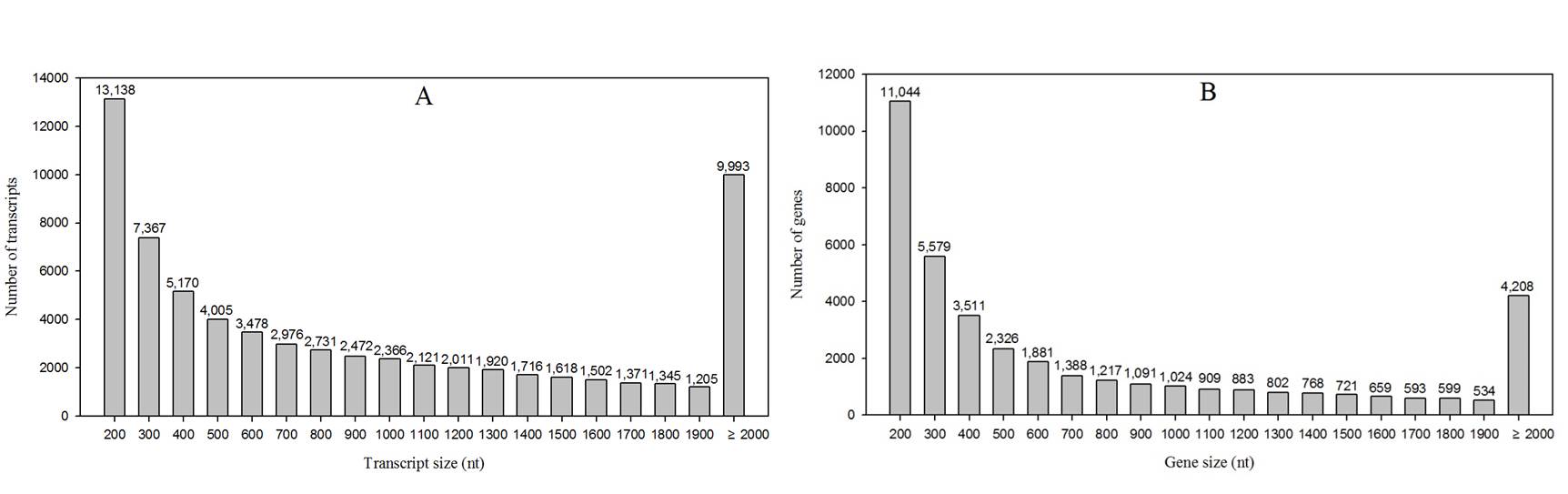


**FIGURE S2 | The length distributions of assembled transcripts and genes.**

**A**, The length distributions of transcripts; **B**, The length distributions of genes.
