# Supplementary material for: Integrated sRNAome and RNA-Seq analysis reveals miRNA effects on betalain biosynthesis in pitaya
Source: BMC Plant Biol. 2020 Sep 22;20:437. doi: 10.1186/s12870-020-02622-x (PMC7510087; doi:10.1186/s12870-020-02622-x)
Supplement: Supplementary file 3 — Additional file 3: Figure S3. GC content distributions of assembled transcripts and genes. A, GC content distributions of transcripts; B, GC content distributions of genes. [file 12870_2020_2622_MOESM3_ESM.docx]

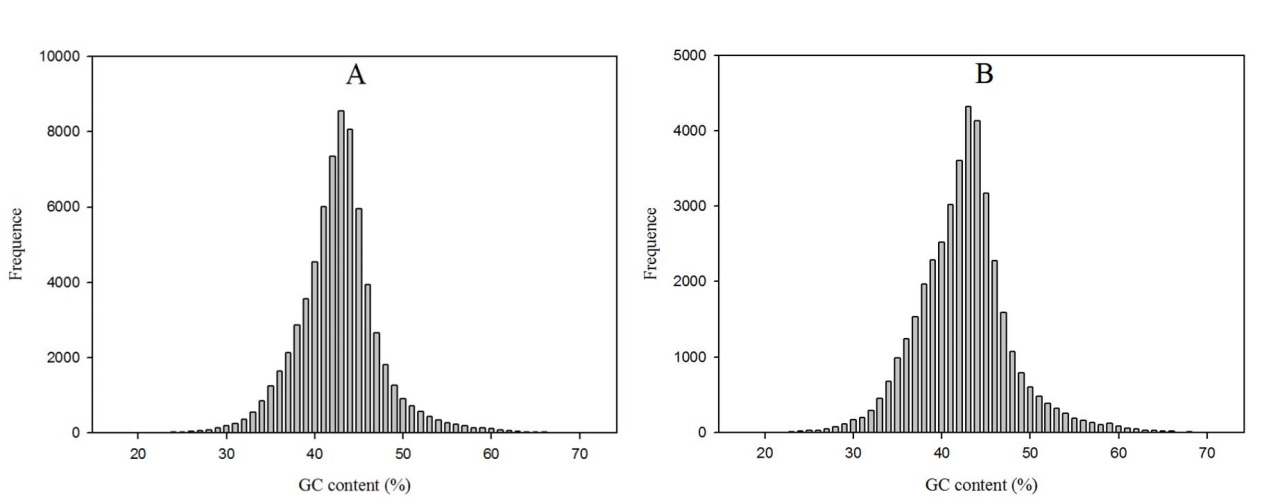


**FIGURE S3 | GC content distributions of assembled transcripts and genes.**

**A**, GC content distributions of transcripts; **B**, GC content distributions of genes.
