# Supplementary figures and images for: Integrated sRNAome and RNA-Seq analysis reveals miRNA effects on betalain biosynthesis in pitaya
Source: BMC Plant Biol. 2020 Sep 22;20:437. doi: 10.1186/s12870-020-02622-x (PMC7510087; doi:10.1186/s12870-020-02622-x)

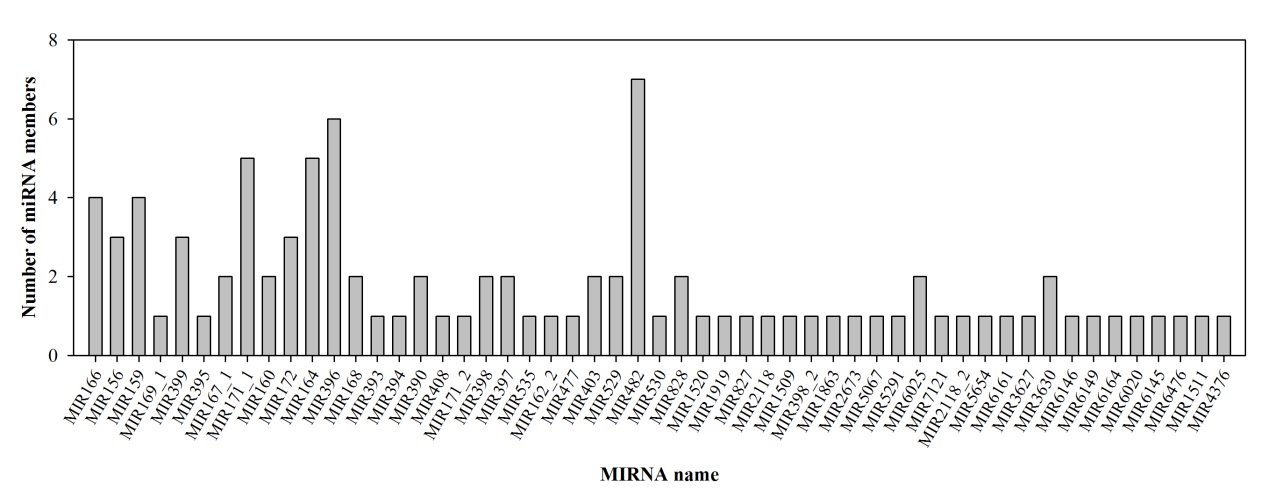


**FIGURE S4 | Number of miRNA family members in pitaya pulp.**

Supplement: Supplementary file 4 — Additional file 4: Figure S4. Number of miRNA family members in pitaya pulp. [file 12870_2020_2622_MOESM4_ESM.docx]
