# Supplementary material for: Integrated sRNAome and RNA-Seq analysis reveals miRNA effects on betalain biosynthesis in pitaya
Source: BMC Plant Biol. 2020 Sep 22;20:437. doi: 10.1186/s12870-020-02622-x (PMC7510087; doi:10.1186/s12870-020-02622-x)
Supplement: Supplementary file 5 — Additional file 5: Figure S5. The sequences of 17 target genes from the two pitaya cultivars. ‘-W’, ‘Guanhuabai’ pitaya. ‘-R’, ‘Guanhuahong’ pitaya. Red lines indicate sequence of target genes primers for real-time PCR. Green lines indicate outer specific primers for nested PCR. Blue lines indicate inner specific primers for nested PCR. [file 12870_2020_2622_MOESM5_ESM.docx]

*HmTT2-like*


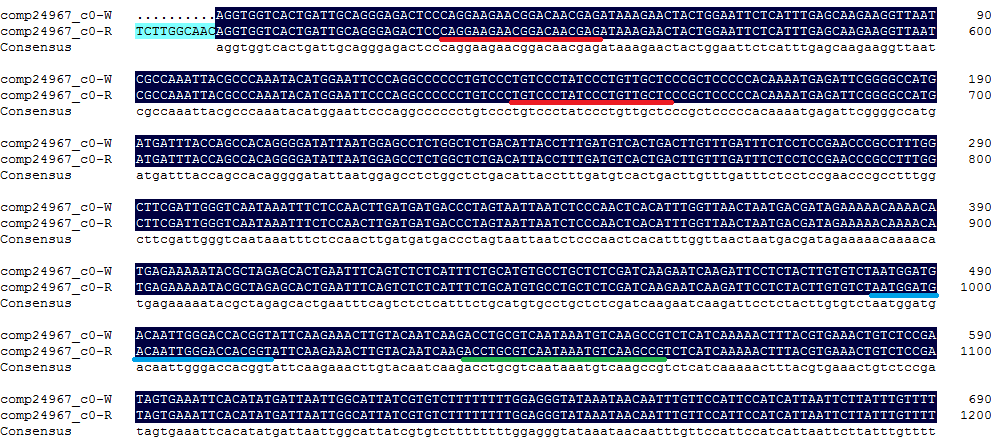


*HmCYP71A8-like*


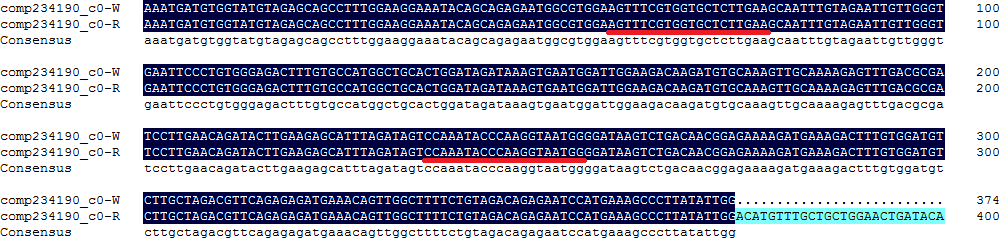


*HmCYP83B1-like*


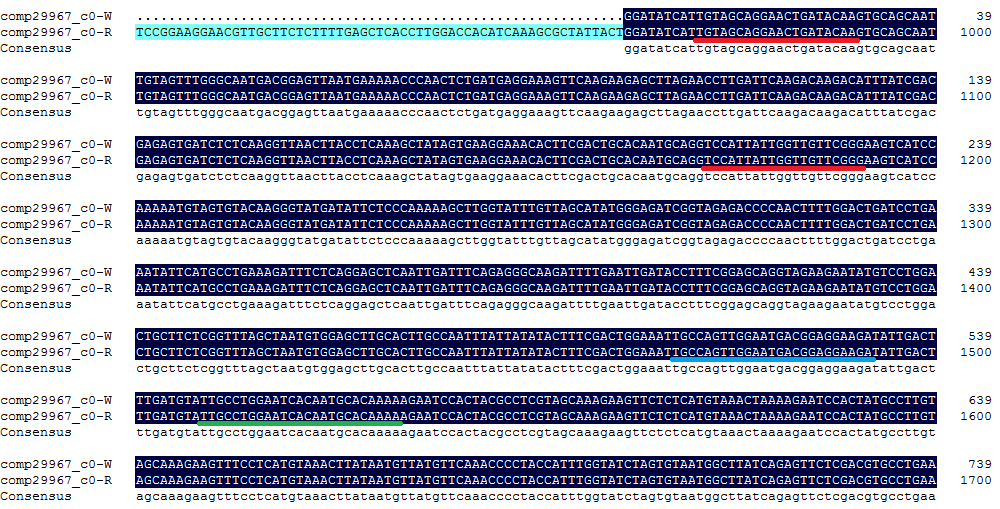


*HmGmSGT2-like*


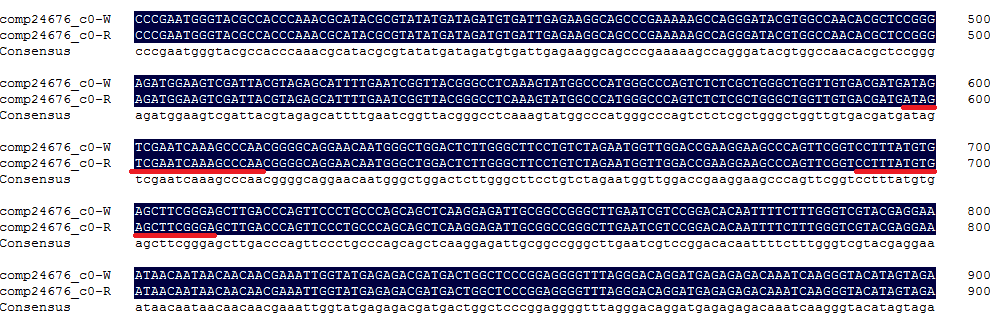


*HmMYB12-like*


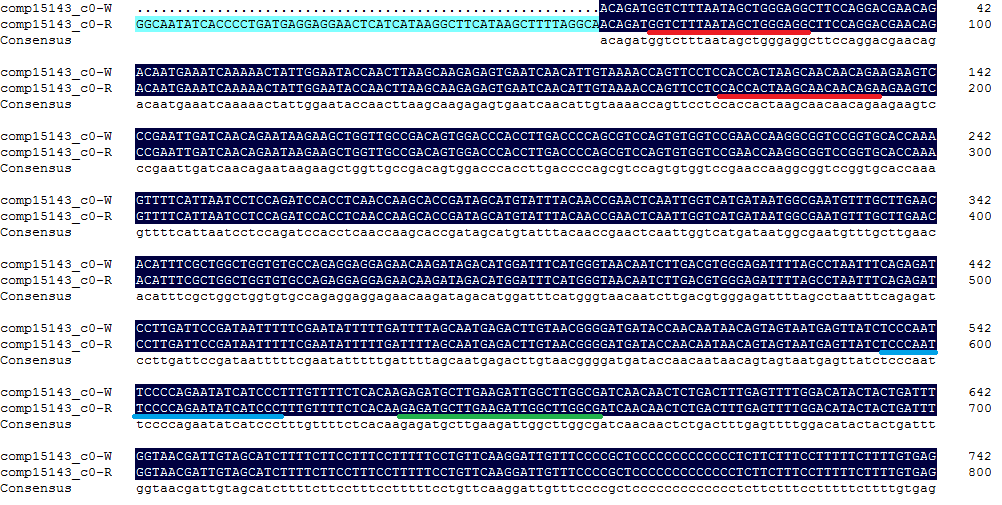


*HmMYBC1-like*


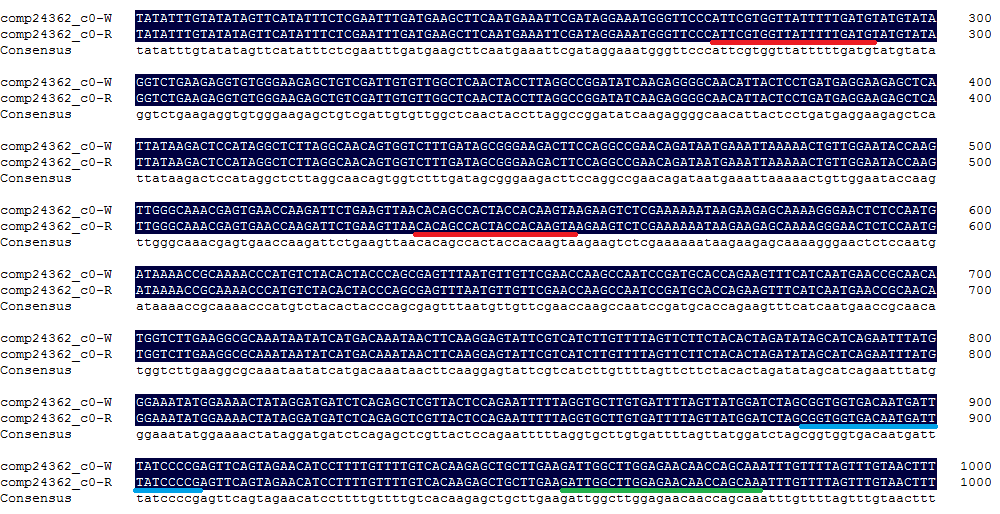


*HmMYB2-like*


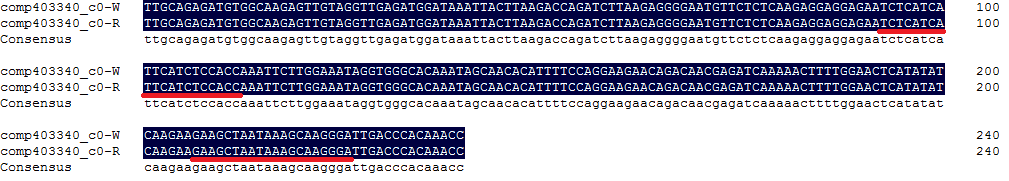


*HmMYB315-like*


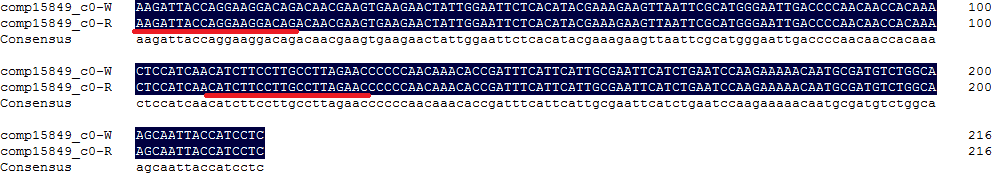


*Hpcyt P450-like3*
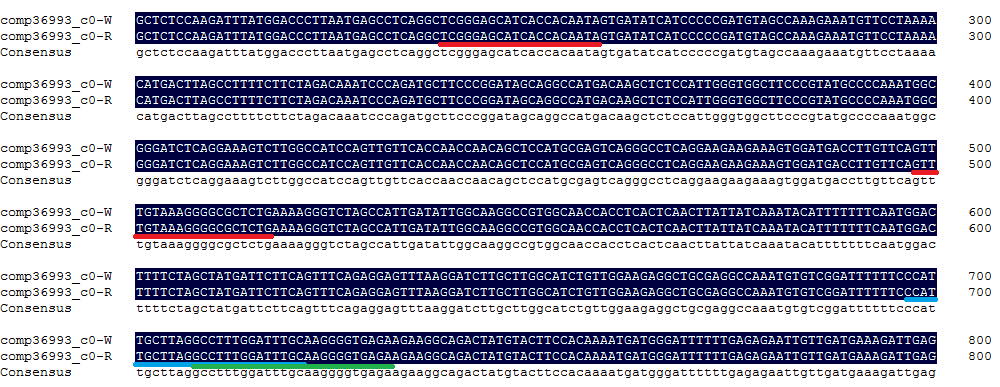


*Hpcyt P450-like2*
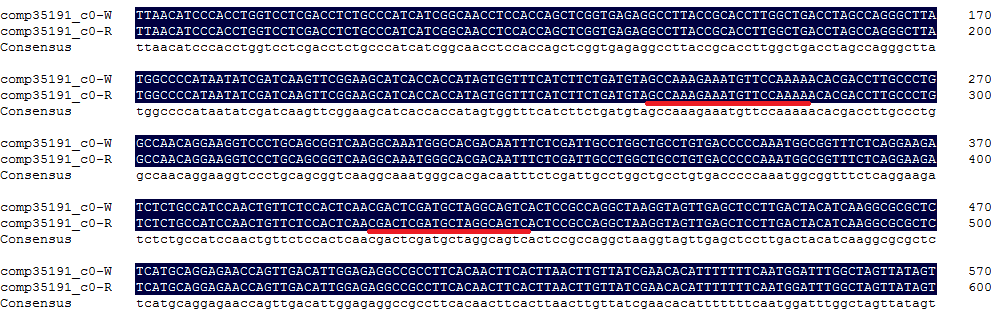


*HmTPST-like*
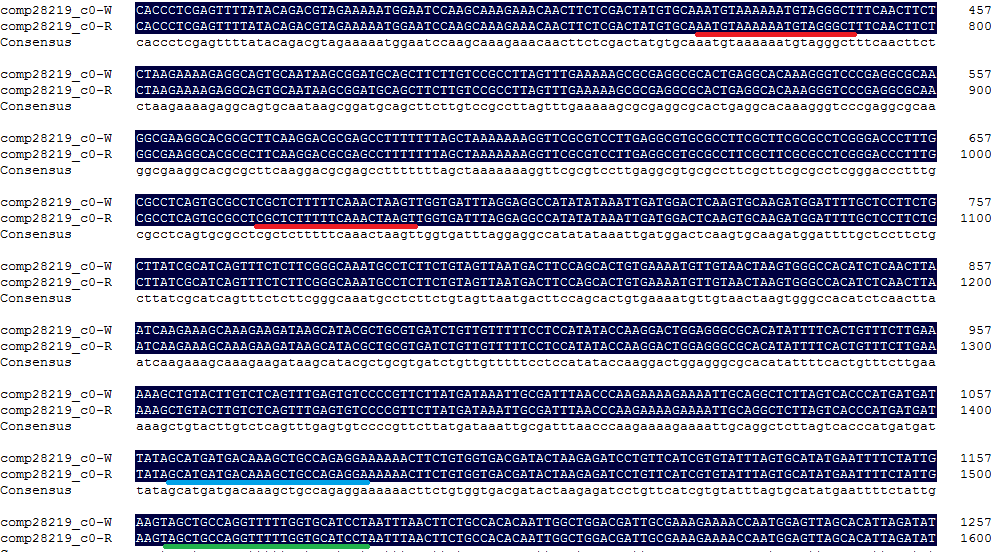


*HmSPL6-like*
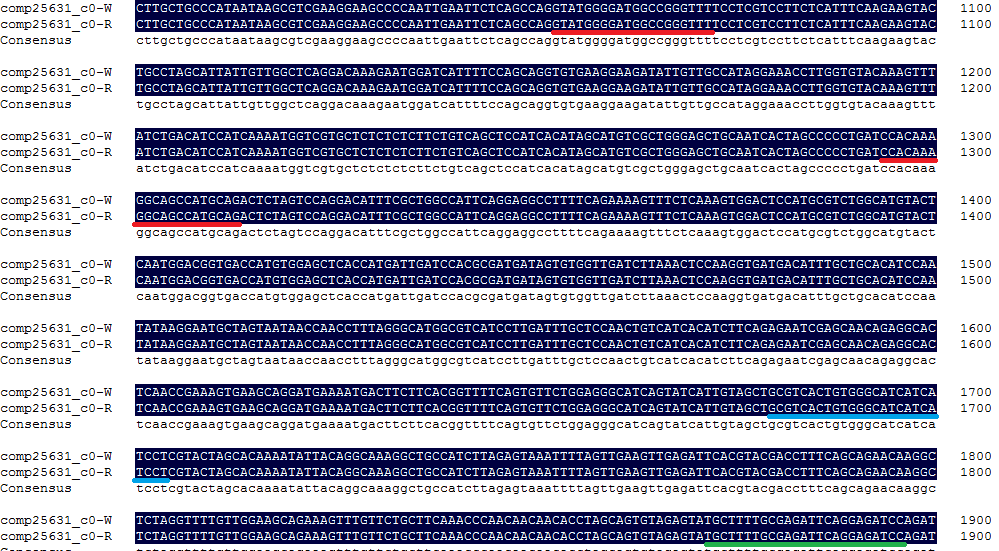


*HmWDTC1-like*


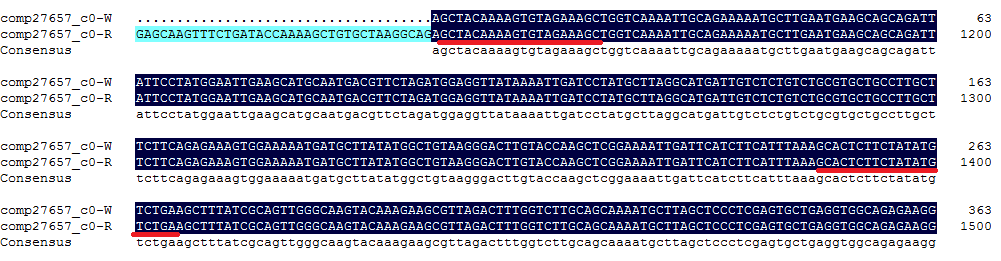


*HmBHLH155-like*


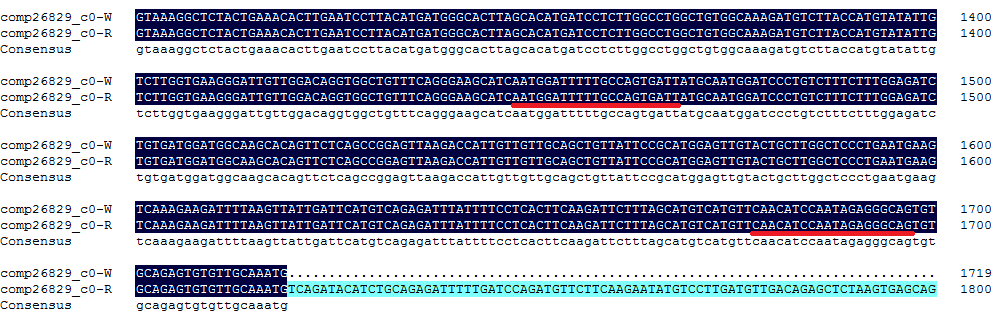


*HmSPL16-like*


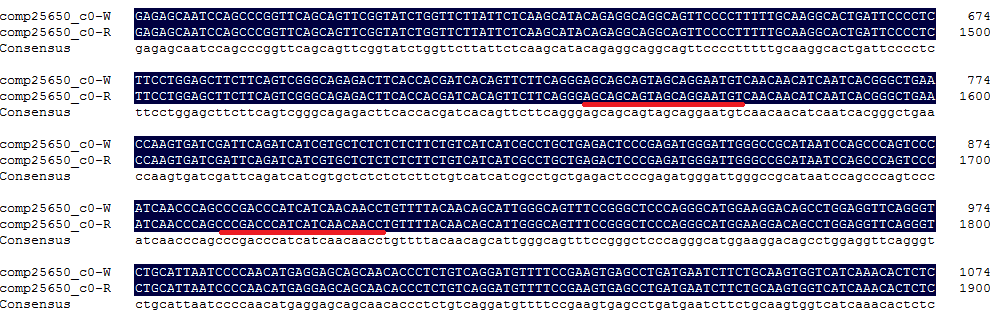


*HmSEC-like*


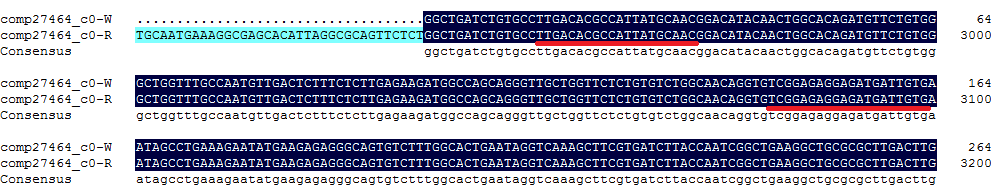


*HmMYB330-like*


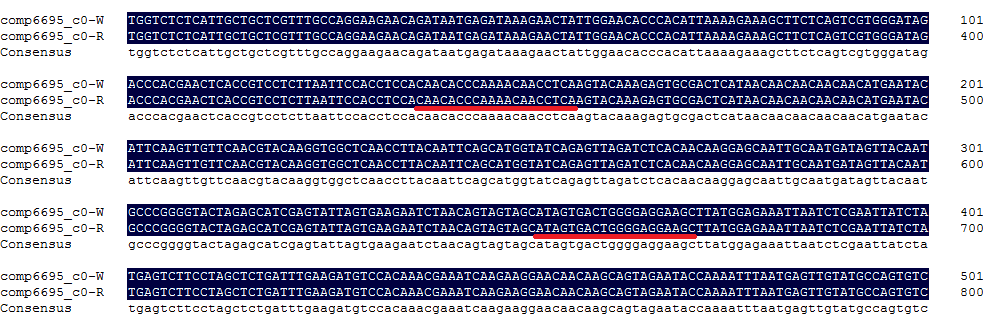


**FIGURE S5 | The sequences of 17 target genes from the two pitaya cultivars.**

‘-W’, ‘Guanhuabai’ pitaya. ‘-R’, ‘Guanhuahong’ pitaya. Red lines indicate sequence of target genes primers for real-time PCR. Green lines indicate outer specific primers for nested PCR. Blue lines indicate inner specific primers for nested PCR.
