# Supplementary material for: Integrated sRNAome and RNA-Seq analysis reveals miRNA effects on betalain biosynthesis in pitaya
Source: BMC Plant Biol. 2020 Sep 22;20:437. doi: 10.1186/s12870-020-02622-x (PMC7510087; doi:10.1186/s12870-020-02622-x)
Supplement: Supplementary file 6 — Additional file 6: Figure S6. Transient expressions of miRNAs and their target genes (all). A1-A49, bright field; B1-B49, eGFP; C1-C49, merge; A1-C1, 35 s::eGFP; A2-C2, WT; A3-C3, 35 s::Pre-miR164; A4-C4, 35 s::Pre-Hmo-miR6020; A5-C5, 35 s::HmCYP71A8-like::eGFP; A6-C6, 35 s::positive control HmCYP71A8-like::eGFP; A7-C7, 35 s::negative control HmCYP71A8-like::eGFP; A8-C8, 35 s::HmCYP71A8-like::eGFP+ 35 s::Pre-miR164; A9-C9, 35 s::HmCYP71A8-like::eGFP+ 35 s::Pre-Hmo-miR6020; A10-C10, 35 s::positive control HmCYP71A8-like::eGFP+ 35 s::Pre-Hmo-miR6020; A11-C11, 35 s::negative control HmCYP71A8-like::eGFP+ 35 s::Pre-Hmo-miR6020; A12-C12, 35 s::Pre-Hmo-miR858; A13-C13, 35 s::HmMYB12-like::eGFP; A14-C14, 35 s::positive control HmMYB12-like::eGFP; A15-C15, 35 s::negative control HmMYB12-like::eGFP; A16-C16, 35 s::HmMYB12-like::eGFP+ 35 s::Pre-miR164; A17-C17, 35 s::HmMYB12-like::eGFP+ 35 s::Pre-Hmo-miR858; A18-C18, 35 s::positive control HmMYB12-like::eGFP+ 35 s::Pre-Hmo-miR858; A19-C19, 35 s::negative control HmMYB12-like::eGFP+ 35 s::Pre-Hmo-miR858; A20-C20, 35 s::HmMYBC1-like::eGFP; A21-C21, 35 s::HmMYBC1-like::eGFP+ 35 s::Pre-miR164; A22-C22, 35 s::HmMYBC1-like::eGFP+ 35 s::Pre-Hmo-miR858; A23-C23, 35 s::HmMYB2-like::eGFP; A24-C24, 35 s::HmMYB2-like::eGFP+ 35 s::Pre-miR164; A25-C25, 35 s::HmMYB2-like::eGFP+ 35 s::Pre-Hmo-miR858; A26-C26, 35 s::Pre-Hmo-miR160a; A27-C27, 35 s::Hpcyt P450-like2::eGFP; A28-C28, 35 s::positive control Hpcyt P450-like2::eGFP; A29-C29, 35 s::negative control Hpcyt P450-like2::eGFP; A30-C30, 35 s::Hpcyt P450-like2::eGFP+ 35 s::Pre-miR164; A31-C31, 35 s::Hpcyt P450-like2::eGFP+ 35 s::Pre-Hmo-miR160a; A32-C32, 35 s::positive control Hpcyt P450-like2::eGFP+ 35 s::Pre-Hmo-miR160a; A33-C33, 35 s::negative control Hpcyt P450-like2::eGFP+ 35 s::Pre-Hmo-miR160a; A34-C34, 35 s::Pre-Hmo-novel-2; A35-C35, 35 s::HmCYP83B1-like::eGFP; A36-C36, 35 s::positive control HmCYP83B1-like::eGFP; A37-C37, 35 s::negative control HmCYP83B1-like::eGFP; A38-C38, 35 s::H [file 12870_2020_2622_MOESM6_ESM.docx]

**
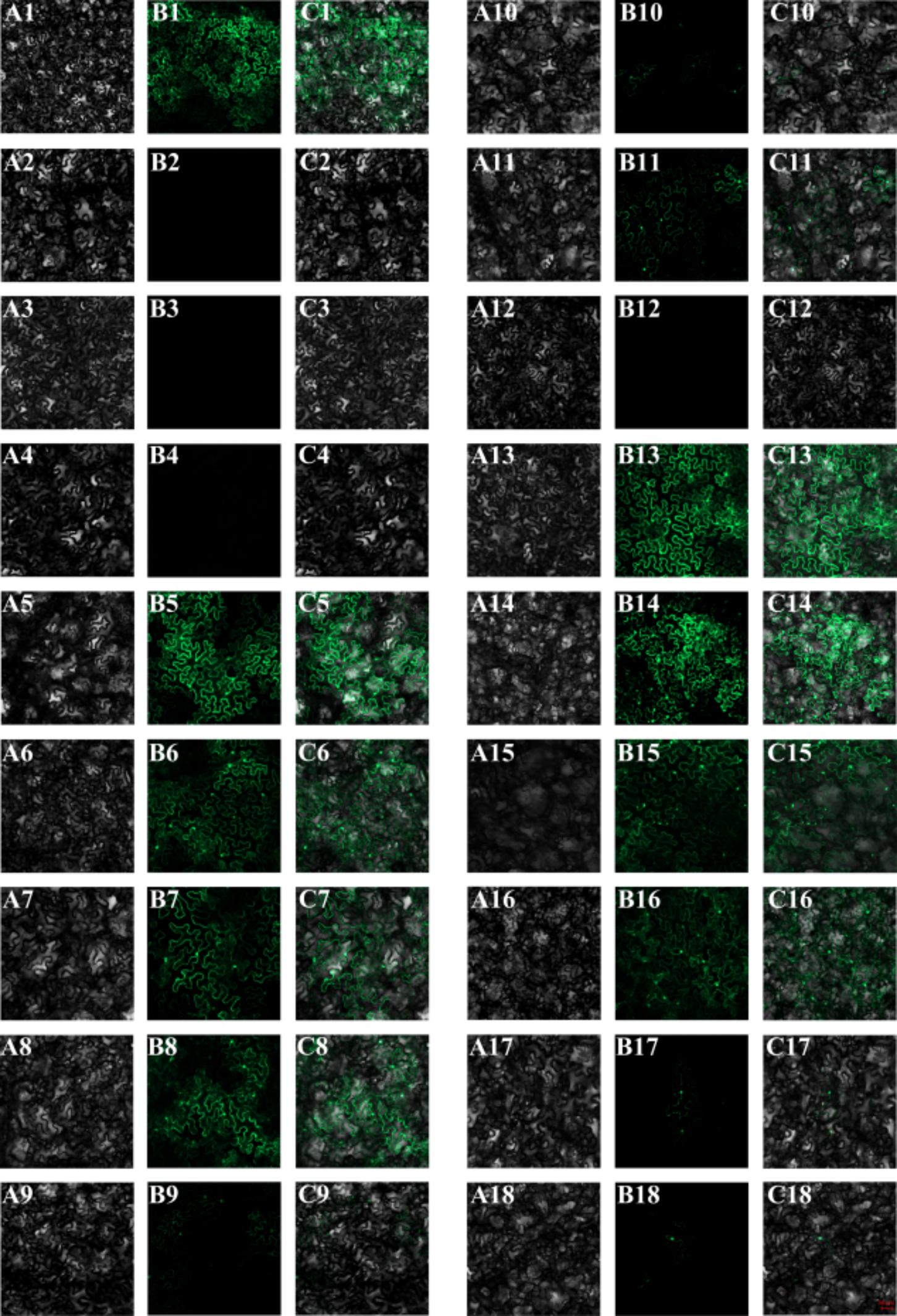
**

**
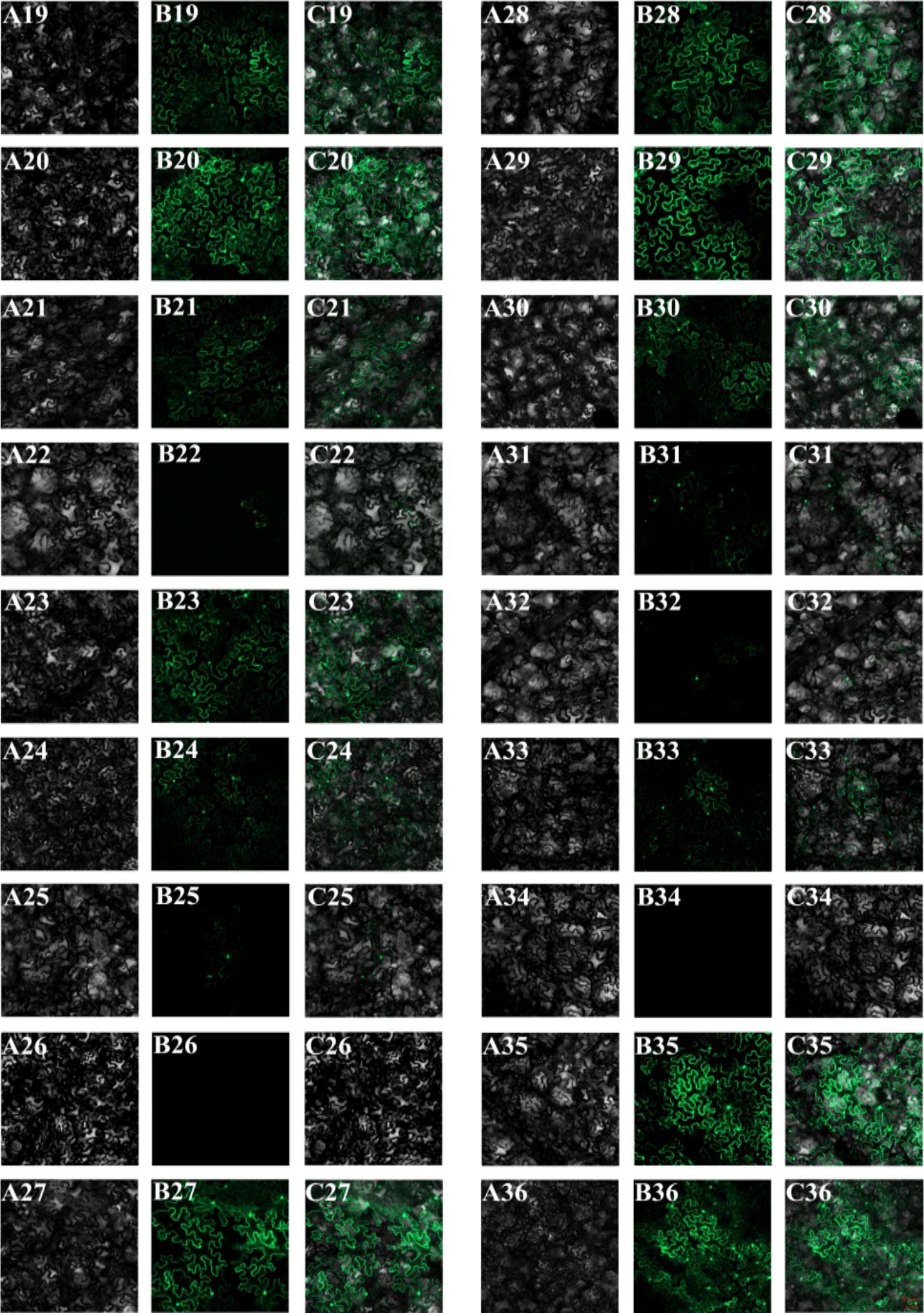
**

**
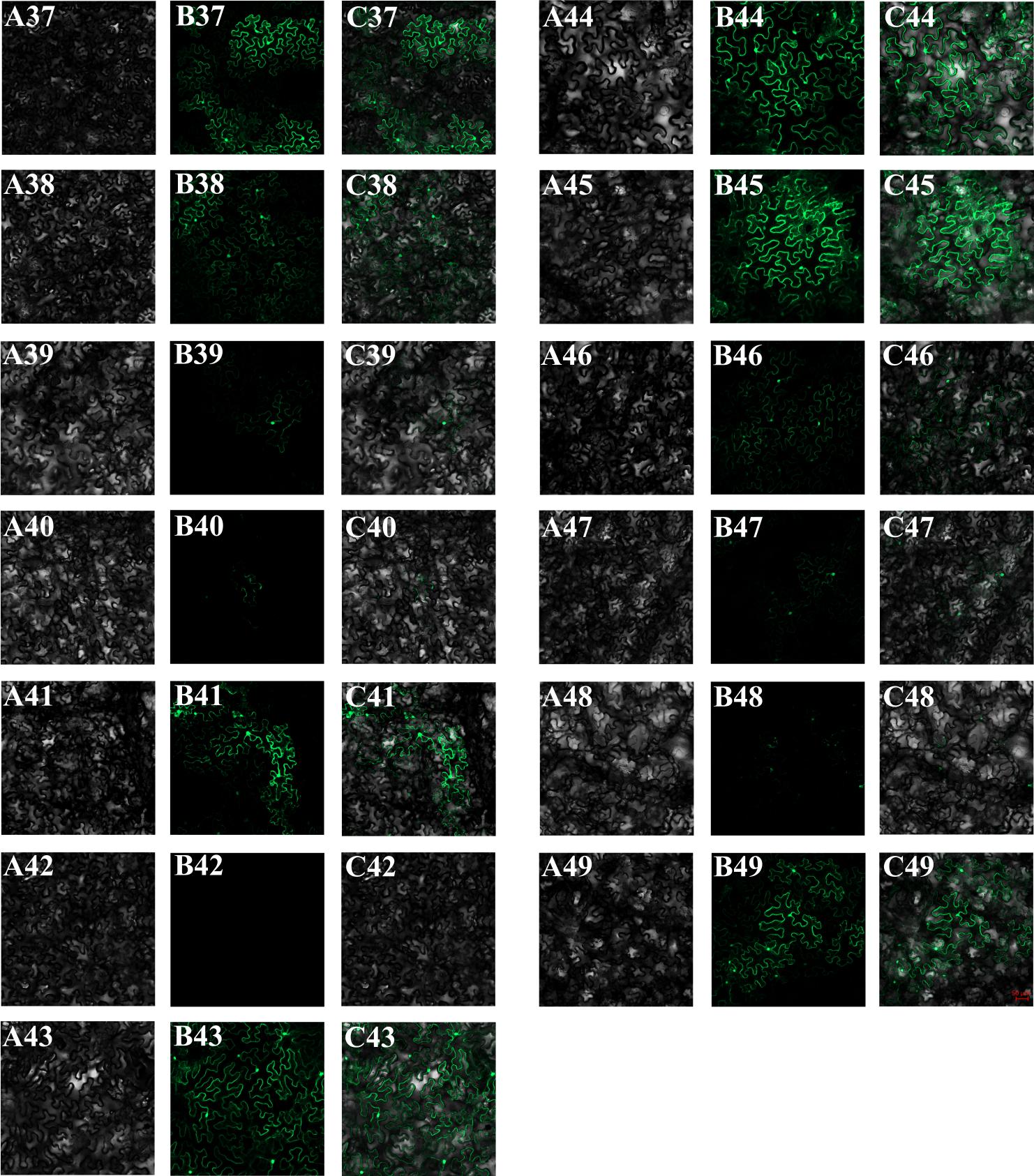
**

**FIGURE S6 | Transient expressions of miRNAs and their target genes (all).**

A1-A49, bright field; B1-B49, eGFP; C1-C49, merge; A1-C1, 35s::eGFP; A2-C2, WT; A3-C3, 35s::Pre-miR164; A4-C4, 35s::Pre-Hmo-miR6020; A5-C5, 35s::*HmCYP71A8-like*::eGFP; A6-C6, 35s::positive control *HmCYP71A8-like*::eGFP; A7-C7, 35s::negative control *HmCYP71A8-like*::eGFP; A8-C8, 35s::*HmCYP71A8-like*::eGFP+35s::Pre-miR164; A9-C9, 35s::*HmCYP71A8-like*::eGFP+35s::Pre-Hmo-miR6020; A10-C10, 35s::positive control *HmCYP71A8-like*::eGFP+35s::Pre-Hmo-miR6020; A11-C11, 35s::negative control *HmCYP71A8-like*::eGFP+35s::Pre-Hmo-miR6020; A12-C12, 35s::Pre-Hmo-miR858; A13-C13, 35s::*HmMYB12-like*::eGFP; A14-C14, 35s::positive control *HmMYB12-like*::eGFP; A15-C15, 35s::negative control *HmMYB12-like*::eGFP; A16-C16, 35s::HmMYB12-like::eGFP+35s::Pre-miR164; A17-C17, 35s::HmMYB12-like::eGFP+35s::Pre-Hmo-miR858; A18-C18, 35s::positive control HmMYB12-like::eGFP+35s::Pre-Hmo-miR858; A19-C19, 35s::negative control HmMYB12-like::eGFP+35s::Pre-Hmo-miR858; A20-C20, 35s::HmMYBC1-like::eGFP; A21-C21, 35s::HmMYBC1-like::eGFP+35s::Pre-miR164; A22-C22, 35s::HmMYBC1-like::eGFP+35s::Pre-Hmo-miR858; A23-C23, 35s::HmMYB2-like::eGFP; A24-C24, 35s::HmMYB2-like::eGFP+35s::Pre-miR164; A25-C25, 35s::HmMYB2-like::eGFP+35s::Pre-Hmo-miR858; A26-C26, 35s::Pre-Hmo-miR160a; A27-C27, 35s::Hpcyt P450-like2::eGFP; A28-C28, 35s::positive control Hpcyt P450-like2::eGFP; A29-C29, 35s::negative control Hpcyt P450-like2::eGFP; A30-C30, 35s::Hpcyt P450-like2::eGFP+35s::Pre-miR164; A31-C31, 35s::Hpcyt P450-like2::eGFP+35s::Pre-Hmo-miR160a; A32-C32, 35s::positive control Hpcyt P450-like2::eGFP+35s::Pre-Hmo-miR160a; A33-C33, 35s::negative control Hpcyt P450-like2::eGFP+35s::Pre-Hmo-miR160a; A34-C34, 35s::Pre-Hmo-novel-2; A35-C35, 35s::HmCYP83B1-like::eGFP; A36-C36, 35s::positive control HmCYP83B1-like::eGFP; A37-C37, 35s::negative control HmCYP83B1-like::eGFP; A38-C38, 35s::HmCYP83B1-like::eGFP+35s::Pre-miR164; A39-C39, 35s::HmCYP83B1-like::eGFP+35s::Pre-Hmo-novel-2; A40-C40, 35s::positive control HmCYP83B1-like::eGFP+35s::Pre-Hmo-novel-2; A41-C41, 35s::negative control HmCYP83B1-like::eGFP+35s::Pre-Hmo-novel-2; A42-C42, 35s::Pre-Hmo-novel-15; A43-C43, 35s::HmTPST-like::eGFP; A44-C44, 35s::positive control HmTPST-like::eGFP; A45-C45, 35s::negative control HmTPST-like::eGFP; A46-C46, 35s::HmTPST-like::eGFP+35s::Pre-miR164; A47-C47, 35s::HmTPST-like::eGFP+35s::Pre-Hmo-novel-15; A48-C48, 35s::positive control HmTPST-like::eGFP+35s::Pre-Hmo-novel-15; A49-C49, 35s::negative control HmTPST-like::eGFP+ 35s::Pre-Hmo-novel-15.
