# Supplementary material for: Integrated sRNAome and RNA-Seq analysis reveals miRNA effects on betalain biosynthesis in pitaya
Source: BMC Plant Biol. 2020 Sep 22;20:437. doi: 10.1186/s12870-020-02622-x (PMC7510087; doi:10.1186/s12870-020-02622-x)
Supplement: Supplementary file 7 — Additional file 7: Figure S7. Different fruit developmental stages of ‘Guanhuahong’ (A) and ‘Guanhuabai’ (B) pitayas. A1 and B1, 13 d; A2 and B2, 16 d; A3 and B3, 19 d; A4 and B4, 23 d; A5 and B5, 25 d; A6 and B6, 27 d; A7 and B7, 29 d. Bar = 2.0 cm. [file 12870_2020_2622_MOESM7_ESM.docx]

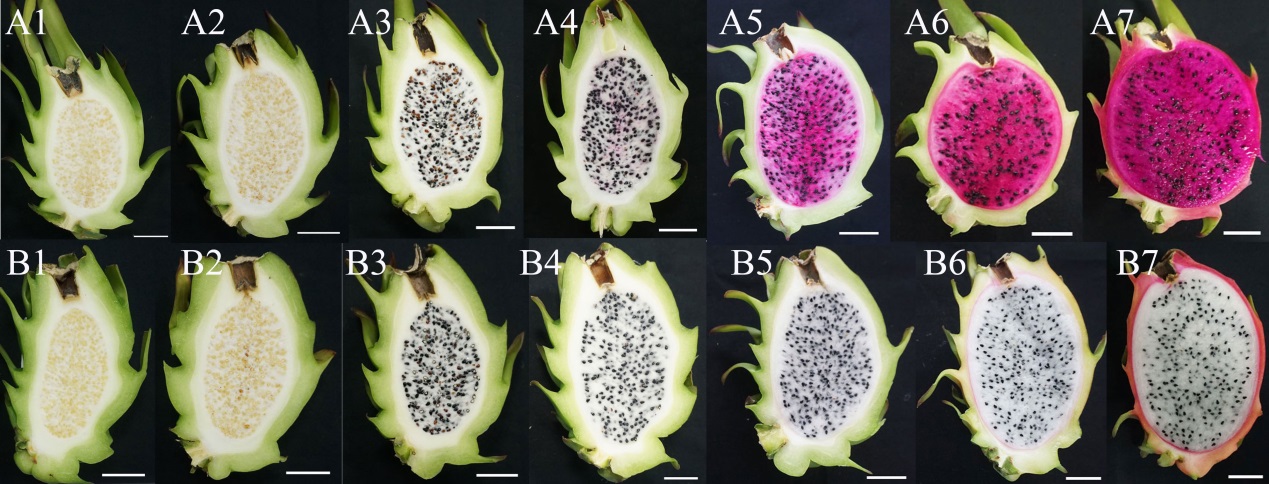


**FIGURE S7 | Different fruit developmental stages of Guanhuahong (A) and Guanhuabai (B) pitayas.**

A1 and B1, 13 d; A2 and B2, 16 d; A3 and B3, 19 d; A4 and B4, 23 d; A5 and B5, 25 d; A6 and B6, 27 d; A7 and B7, 29 d. Bar=2.0 cm.
