# Supplementary material for: Integrated sRNAome and RNA-Seq analysis reveals miRNA effects on betalain biosynthesis in pitaya
Source: BMC Plant Biol. 2020 Sep 22;20:437. doi: 10.1186/s12870-020-02622-x (PMC7510087; doi:10.1186/s12870-020-02622-x)
Supplement: Supplementary file 8 — Additional file 8: Table S1. Statistics of miRNA sequences in pitaya. [file 12870_2020_2622_MOESM8_ESM.docx]

**TABLE S1** **Statistics of miRNA sequences in pitaya**

|  | Hp19d_1 | | Hp19d_2 | | Hp25d_1 | | Hp25d_2 | | Hp29d_1 | | Hp29d_2 | |
| --- | --- | --- | --- | --- | --- | --- | --- | --- | --- | --- | --- | --- |
| lib | Total | % of Total | Total | % of Total | Total | % of Total | Total | % of Total | Total | % of  Total | Total | % of  Total |
| Raw reads | 14,962,839 | 100.00 | 14,984,733 | 100.00 | 15,639,258 | 100.00 | 11,097,450 | 100.00 | 11,764,019 | 100.00 | 13,869,942 | 100.00 |
| 3ADT& filter | 3,261,446 | 21.80 | 3,796,806 | 25.34 | 3,029,548 | 19.37 | 2,320,611 | 20.91 | 2,166,241 | 18.41 | 3,373,433 | 24.32 |
| Junk reads | 12,628 | 0.08 | 7,789 | 0.05 | 41,774 | 0.27 | 20,713 | 0.19 | 23,847 | 0.20 | 16,418 | 0.12 |
| Rfam | 231,864 | 1.55 | 202,787 | 1.35 | 228,182 | 1.46 | 145,837 | 1.31 | 348,656 | 2.96 | 346,469 | 2.50 |
| Repeats | 2,480 | 0.02 | 2,165 | 0.01 | 4,259 | 0.03 | 2,607 | 0.02 | 4,736 | 0.04 | 5,015 | 0.04 |
| valid reads | 11,455,464 | 76.56 | 10,976,049 | 73.25 | 12,337,213 | 78.89 | 8,608,731 | 77.57 | 9,222,106 | 78.39 | 10,130,162 | 73.04 |
| rRNA | 181,858 | 1.22 | 155,328 | 1.04 | 213,591 | 1.37 | 137,111 | 1.24 | 315,843 | 2.68 | 314,833 | 2.27 |
| tRNA | 39,287 | 0.26 | 39,011 | 0.26 | 5,932 | 0.04 | 3,397 | 0.03 | 5,522 | 0.05 | 5,567 | 0.04 |
| snoRNA | 2,139 | 0.01 | 1,418 | 0.01 | 2,393 | 0.02 | 1,499 | 0.01 | 2,430 | 0.02 | 2,410 | 0.02 |
| snRNA | 2,971 | 0.02 | 2,393 | 0.02 | 2,468 | 0.02 | 1,545 | 0.01 | 1,826 | 0.02 | 1,931 | 0.01 |
| other Rfam RNA | 5,609 | 0.04 | 4,637 | 0.03 | 3,798 | 0.02 | 2,285 | 0.02 | 23,035 | 0.20 | 21,728 | 0.16 |
