# Supplementary material for: Integrated sRNAome and RNA-Seq analysis reveals miRNA effects on betalain biosynthesis in pitaya
Source: BMC Plant Biol. 2020 Sep 22;20:437. doi: 10.1186/s12870-020-02622-x (PMC7510087; doi:10.1186/s12870-020-02622-x)
Supplement: Supplementary file 9 — Additional file 9: Table S2. The information of miRNAs in pitaya. [file 12870_2020_2622_MOESM9_ESM.docx]

**TABLE S2** **The information of miRNAs in pitaya**

| Accession names | miRNA names | miRNA sequences | Len | Hairpin  Len | CG% | dG | Hp19d (norm) | Hp25d (norm) | Hp29d (norm) |
| --- | --- | --- | --- | --- | --- | --- | --- | --- | --- |
| mdm-miR1511 | Hmo-miR1511 | ACCTAGCTCTGATACCATGAA | 21 | 83 | 36.20 | -43.70 | 1.57 | 3.42 | 0.96 |
| gma-miR1520j_L-3_1ss23AT | Hmo-miR1520 | AACGTGACACATGACAATCTA | 21 | 176 | 34.70 | -82.90 | 0.00 | 0.74 | 0.00 |
| ssp-miR156_L+1R-1_1ss4AC | Hmo-miR156 | TTGCCAGAAGAGAGTGAGCAC | 21 | 104 | 47.50 | -198.75 | 29.61 | 65.31 | 75.26 |
| aly-miR157d-3p | Hmo-miR157a | GCTCTCTATGCTTCTGTCATC | 21 | 256 | 35.50 | -85.90 | 11.09 | 9.79 | 12.36 |
| aly-miR157d-5p_L+1_1ss4AC | Hmo-miR157b | CTGCCAGAAGATAGAGAGCAC | 21 | 256 | 35.50 | -85.90 | 49.94 | 105.35 | 148.32 |
| nta-miR159 | Hmo-miR159a | TTTGGATTGAAGGGAGCTCTA | 21 | 178 | 43.50 | -86.10 | 197.90 | 182.27 | 108.18 |
| zma-miR159c-3p_L+2R-1 | Hmo-miR159b | GGCTTGGATTGAAGGGAGCTCC | 22 | 215 | 55.30 | -107.50 | 20.01 | 7.12 | 8.90 |
| zma-miR159c-5p_L-1R-2_1ss13AG | Hmo-miR159c | AGCTCCCTTCGGTCCAAT | 18 | 215 | 55.30 | -107.50 | 0.73 | 0.00 | 0.46 |
| aau-miR160_L-4R+1 | Hmo-miR160a | ATACAGGGAGCCAGGCAA | 18 | 80 | 56.80 | -29.20 | 0.00 | 0.00 | 1.01 |
| ttu-miR160_1ss21AG | Hmo-miR160b | TGCCTGGCTCCCTGTATGCCG | 21 | 95 | 52.00 | -52.90 | 164.50 | 189.25 | 76.49 |
| osa-miR162a_R+2 | Hmo-miR162 | TCGATAAACCTCTGCATCCAGTT | 23 | 171 | 52.60 | -71.20 | 0.00 | 3.39 | 2.79 |
| ptc-miR164a | Hmo-miR164a | TGGAGAAGCAGGGCACGTGCA | 21 | 153 | 45.80 | -55.80 | 4478.72 | 27330.72 | 89761.94 |
| sly-miR164a-3p_1ss12TC | Hmo-miR164b | CATGTGCCTGTCTTCCCCATC | 21 | 153 | 45.80 | -55.80 | 1852.24 | 7112.16 | 24054.73 |
| sbi-miR164c_1ss20AC | Hmo-miR164c | TGGAGAAGCAGGACACGTGCG | 21 | 148 | 62.10 | -88.90 | 0.00 | 0.00 | 0.25 |
| csi-MIR164-p3_1ss2GA | Hmo-MIR164-p3 | TATGTGTGTGTGTGTGTGTCT | 21 | 74 | 23.40 | -36.60 | 0.73 | 1.74 | 1.37 |
| csi-MIR164-p5_1ss8GA | Hmo-MIR164-p5 | TGTGTGTATGTGTGTGTCTGT | 21 | 54 | 38.30 | -36.90 | 1.47 | 2.63 | 1.97 |
| cme-miR166e_L+2R-2 | Hmo-miR166a | TCTCGGACCAGGCTTCATTCC | 21 | 270 | 31.90 | -69 | 32.16 | 37.83 | 33.40 |
| zma-miR166l-3p_1ss4GA | Hmo-miR166b | TCGAACCAGGCTTCATTCCTC | 21 | 147 | 62.80 | -71.70 | 11.09 | 15.58 | 10.94 |
| zma-miR166m-5p_1ss4AC | Hmo-miR166c | GGACTGTTGGCTGGCTCGAGG | 21 | 147 | 62.80 | -71.70 | 1.98 | 6.15 | 7.93 |
| ptc-miR166p_1ss21TC | Hmo-miR166d | TCGGACCAGGCTCCATTCCTC | 21 | 141 | 46.60 | -51.60 | 0.00 | 0.14 | 0.00 |
| ahy-miR167-3p_L+3_2ss15CT18TC | Hmo-miR167a | ATTAGATCATGTGGTAGCTTCACC | 24 | 128 | 44.80 | -61.80 | 9.88 | 16.91 | 12.31 |
| mtr-miR167b-5p | Hmo-miR167b | TGAAGCTGCCAGCATGATCTG | 21 | 128 | 44.80 | -61.80 | 1105.87 | 1532.28 | 1251.90 |
| aly-miR168a-3p_L+3_1ss19CT | Hmo-miR168a | GATCCCGCCTTGCATCAATTGAAT | 24 | 155 | 52.90 | -70.40 | 192.63 | 138.56 | 102.43 |
| aly-miR168a-5p | Hmo-miR168b | TCGCTTGGTGCAGGTCGGGAA | 21 | 155 | 52.90 | -70.40 | 2544.02 | 2730.55 | 3002.13 |
| ghb-miR169a | Hmo-miR169 | TAGCCAAGGATGACTTGCCTG | 21 | 171 | 46.00 | -85.20 | 21.56 | 23.72 | 32.81 |
| ctr-miR171 | Hmo-miR171a | TTGAGCCGCGTCAATATCTCC | 21 | 82 | 41.80 | -113.60 | 0.00 | 1.16 | 0.46 |
| csi-miR171b_1ss10TC | Hmo-miR171b | CGAGCCGAACCAATATCACTC | 21 | 126 | 37.60 | -54.90 | 1.23 | 1.51 | 0.76 |
| ssl-miR171b_1ss21TC | Hmo-miR171c | TTGAGCCGCGCCAATATCACC | 21 | 88 | 46.60 | -38.30 | 156.46 | 71.60 | 39.34 |
| gma-miR171c-3p_1ss19AC | Hmo-miR171d | TTGAGCCGTGCCAATATCCCA | 21 | 104 | 37.50 | -45.90 | 9.11 | 1.53 | 3.44 |
| gma-miR171c-5p_1ss1AC | Hmo-miR171e | CGATATTGGTGCGGTTCAATC | 21 | 104 | 37.50 | -45.90 | 0.43 | 0.16 | 0.11 |
| gma-miR171k-5p_1ss5GA | Hmo-miR171f | CGATATTGGTGAGGTTCAATC | 21 | 88 | 46.60 | -38.30 | 3.24 | 1.34 | 1.04 |
| gma-miR172a_1ss1AC | Hmo-miR172a | CGAATCTTGATGATGCTGCAT | 21 | 150 | 46.50 | -65.30 | 460.97 | 369.05 | 227.33 |
| gma-miR172b-5p_L-2R+2 | Hmo-miR172b | AGCATCATCAAGATTCACAT | 20 | 150 | 46.50 | -65.30 | 5.70 | 3.65 | 1.69 |
| nta-miR172d_1ss4AC | Hmo-miR172c | AGACTCTTGATGATGCTGCAT | 21 | 167 | 39.00 | -129.40 | 33.56 | 28.25 | 16.88 |
| cme-miR1863_L+1R-1_1ss20GA | Hmo-miR1863a | AAGCTCTGATACCATGTTAAATTT | 24 | 595 | 50.60 | -159 | 0.00 | 0.86 | 0.87 |
| osa-miR1863b_1ss23TC | Hmo-miR1863b | AGCTCTGATACCATGTTAACTGCT | 24 | 355 | 44.20 | -139.30 | 1.73 | 1.32 | 0.59 |
| osa-miR1873_L+1R-4_1ss21GA | Hmo-miR1873 | CTCAACATGGTATCAGAGCTA | 21 | 196 | 39.60 | -77.30 | 0.00 | 0.79 | 0.00 |
| stu-miR1919-5p_R+2 | Hmo-miR1919 | TGTCGCAGATGACTTTCGCCCTT | 23 | 94 | 42.70 | -35.90 | 1.96 | 0.87 | 1.19 |
| mdm-miR2118a | Hmo-miR2118a | CTACCGATGCCACTAAGTCCCA | 22 | 151 | 46.40 | -78.70 | 0.00 | 2.02 | 0.00 |
| hbr-miR2118_L+5R-1_1ss12GA | Hmo-miR2118b | GAGTGGAAATGAGTGGATGGGAGTG | 25 | 137 | 50.70 | -75.70 | 0.00 | 0.21 | 0.00 |
| mtr-miR2673a_L+1R-2_1ss3CT | Hmo-miR2673a | TCTTCTTCCTCTTCCTCTTCC | 21 | 242 | 43.30 | -71.60 | 0.99 | 0.64 | 0.96 |
| peu-MIR2916-p3_1ss8TC | Hmo-MIR2916-p3 | CAGGGATCGGCGGATGTTGCT | 21 | 57 | 53.60 | -20.50 | 2331.68 | 737.54 | 3764.72 |
| peu-MIR2916-p5_1ss5AG | Hmo-MIR2916-p5 | TACCGTCCTAGTCTCAACCATA | 22 | 57 | 53.60 | -20.50 | 806.87 | 141.31 | 2298.45 |
| osa-miR319a-3p.2-3p_1ss9AG | Hmo-miR319 | TTGGACTGGAGGGTGCTCCC | 20 | 189 | 50.80 | -90.60 | 0.00 | 0.58 | 0.00 |
| ppe-miR3627-5p_R-2_1ss11GA | Hmo-miR3627 | TCGCAGGAGAAATGGCACTG | 20 | 104 | 48.10 | -58 | 0.99 | 0.21 | 0.00 |
| han-miR3630-3p_L-1R-2_1ss2GA | Hmo-miR3630a | ATGGGAATCTCTCTGATGC | 19 | 93 | 36.30 | -19.50 | 0.73 | 0.11 | 0.25 |
| vvi-miR3630-3p_L-2 | Hmo-miR3630b | TGGGAATCTCTCTGATGCAC | 20 | 102 | 44.10 | -34.70 | 21.23 | 4.66 | 6.52 |
| aly-miR390a-3p | Hmo-miR390a | CGCTATCCATCCTGAGTTTCA | 21 | 107 | 38.80 | -51.30 | 186.71 | 57.48 | 84.87 |
| aly-miR390a-5p | Hmo-miR390b | AAGCTCAGGAGGGATAGCGCC | 21 | 107 | 38.80 | -51.30 | 29.35 | 5.47 | 6.14 |
| cme-miR393a_R+1 | Hmo-miR393 | TCCAAAGGGATCGCATTGATCC | 22 | 146 | 37.40 | -41.15 | 696.73 | 560.01 | 404.86 |
| gma-miR394a-5p_1ss2TG | Hmo-miR394 | TGGGCATTCTGTCCACCTCC | 20 | 157 | 44.60 | -78.20 | 6.20 | 1.74 | 2.08 |
| osa-miR395b_L-1 | Hmo-miR395 | TGAAGTGTTTGGGGGAACTC | 20 | 88 | 46.60 | -45.50 | 6.91 | 2.75 | 6.27 |
| csi-miR3952 | Hmo-miR3952 | TGAAGGGCCTTTCTAGAGCAC | 21 | 153 | 47.20 | -59.90 | 0.00 | 0.21 | 0.46 |
| gma-miR396a-5p_L+1_1ss22GT | Hmo-miR396a | CTTCCACAGCTTTCTTGAACTT | 22 | 114 | 39.50 | -44 | 799.45 | 813.04 | 970.62 |
| mtr-miR396b-3p_L+2R-2 | Hmo-miR396b | CGGTTCAATAAAGCTGTGGGA | 21 | 152 | 38.60 | -67.40 | 193.41 | 333.95 | 332.32 |
| mtr-miR396b-3p_1ss7TG | Hmo-miR396c | GTTCAAGAAAGCTGTGGGAAG | 21 | 87 | 35.80 | -37.80 | 16.36 | 24.39 | 31.27 |
| ath-miR396b-3p_L-1R+1_1ss2CT | Hmo-miR396d | TTCAAGAAAGCTGTGGGAAAA | 21 | 150 | 39.50 | -52.30 | 0.73 | 0.79 | 1.47 |
| mtr-miR396b-5p | Hmo-miR396e | TTCCACAGCTTTCTTGAACTG | 21 | 152 | 38.60 | -67.40 | 293677.94 | 440037.56 | 407786.10 |
| lus-miR396d_R+1_1ss20TC | Hmo-miR396f | TCCCACAGCTTTATTGAACCGC | 22 | 150 | 39.50 | -52.30 | 2.72 | 2.98 | 6.08 |
| zma-miR396g-3p_R+2_1ss21AC | Hmo-miR396g | GTTCAAGAAAGCTGTGGAAGCAT | 23 | 114 | 45.70 | -51.90 | 0.73 | 0.43 | 0.46 |
| lus-miR396d_R-1_1ss13AC | Hmo-miR396h | TCCCACAGCTTTCTTGAACT | 20 | 114 | 45.70 | -51.90 | 0.44 | 0.23 | 0.20 |
| hbr-miR396a_R-3 | Hmo-miR396i | CACAGCTTTCTTGAACTT | 18 | 86 | 37.20 | -36 | 44.38 | 33.79 | 35.70 |
| ama-miR396-5p_1ss4CA | Hmo-miR396j | TTCAACAGCTTTCTTGAACTT | 21 | 87 | 35.80 | -37.80 | 3217.87 | 2864.01 | 3341.76 |
| mdm-miR397a_L+6R-2 | Hmo-miR397a | ACATCATTGAGTGCAGCGTTGATGA | 25 | 101 | 41.60 | -41.70 | 0.50 | 0.11 | 0.00 |
| lus-miR397a_L-1R+2 | Hmo-miR397b | TTGAGTGCAGCGTTGATGAAAT | 22 | 118 | 42.50 | -44 | 30.58 | 5.15 | 1.01 |
| aly-miR398a-3p | Hmo-miR398a | TGTGTTCTCAGGTCACCCCTT | 21 | 129 | 49.10 | -72.50 | 64.15 | 27.02 | 25.48 |
| zma-miR398a-3p_R-2 | Hmo-miR398b | TGTGTTCTCAGGTCGCCCC | 19 | 106 | 63.90 | -60 | 88.88 | 11.30 | 6.56 |
| gma-miR398c | Hmo-miR398c | TGTGTTCTCAGGTCGCCCCTG | 21 | 108 | 44.00 | -50.60 | 649.53 | 136.35 | 65.72 |
| ppe-miR399a | Hmo-miR399a | CGCCAAAGGAGAGTTGCCCTT | 21 | 107 | 47.70 | -54.10 | 4.19 | 10.06 | 18.06 |
| stu-miR399a-3p_1ss13GA | Hmo-miR399b | TGCCAAAGGAGAACTGCCCTG | 21 | 62 | 50.00 | -35.10 | 1.73 | 0.43 | 0.25 |
| rco-miR399f_L+1R-1_1ss20CT | Hmo-miR399c | CTGCCAAAGGAGATTTGCTTA | 21 | 85 | 42.50 | -36.70 | 0.00 | 1.01 | 0.00 |
| mdm-miR403a_R+1_2ss20CT21GT | Hmo-miR403a | TTAGATTCACGCACAAACTTTT | 22 | 115 | 47.00 | -43.10 | 0.00 | 0.00 | 0.96 |
| sly-miR403-5p_L-2_1ss15TC | Hmo-miR403b | TTTGTGCGTGAACCTAACA | 19 | 142 | 36.60 | -49.50 | 0.73 | 0.58 | 0.50 |
| mdm-miR408a_1ss1AT | Hmo-miR408 | TTGCACTGCCTCTTCCCTGGC | 21 | 123 | 48.80 | -70.10 | 681.72 | 103.26 | 68.21 |
| sly-miR4376_1ss4CA | Hmo-miR4376 | ACGAAGGAGAGATGATGCTGGA | 22 | 90 | 42.90 | -44.40 | 0.73 | 0.00 | 0.00 |
| csi-miR477b_L+1R-1 | Hmo-miR477 | ACTCTCCCTCAAGGGCTTCTC | 21 | 126 | 47.80 | -60.20 | 0.00 | 1.76 | 0.50 |
| nta-miR482a | Hmo-miR482a | TTTCCAATTCCACCCATTCCTA | 22 | 84 | 40.40 | -40.90 | 3.45 | 1.16 | 2.43 |
| mdm-miR482b | Hmo-miR482b | TCTTTCCTATCCCTCCCATTCC | 22 | 106 | 35.80 | -46 | 0.00 | 2.97 | 0.00 |
| mdm-miR482a-5p_L-1 | Hmo-miR482c | GGAATGGGCTGTTTGGGAAGA | 21 | 155 | 45.80 | -90.60 | 0.00 | 0.79 | 0.46 |
| nta-miR482d | Hmo-miR482d | TTCCCGACTCCCCCCATACCAC | 22 | 98 | 57.00 | -61 | 0.99 | 0.43 | 0.00 |
| mdm-miR482a-3p | Hmo-miR482e | TTCCCAAGCCCGCCCATTCCTA | 22 | 155 | 45.80 | -90.60 | 0.00 | 2.17 | 0.00 |
| stu-miR482a-5p_2ss3AT12AG | Hmo-miR482f | GGTATTGGTGGGTTGGAAAGC | 21 | 84 | 40.40 | -40.90 | 1.73 | 0.43 | 0.00 |
| gra-MIR482d-p3 | Hmo-MIR482g | ATACACACACACACACACACA | 21 | 54 | 38.30 | -36.90 | 5.66 | 9.63 | 10.02 |
| osa-miR5072_L-4_1ss12CT | Hmo-miR5072 | TCCCCAGTGGAGTCGCCA | 18 | 71 | 47.40 | -19.40 | 20.97 | 1.90 | 2.88 |
| osa-miR5077_L-1_1ss5GA | Hmo-miR5077 | TTCACGTCGGGTTCACCA | 18 | 82 | 59.30 | -27.50 | 17.48 | 10.86 | 15.57 |
| rgl-MIR5141-p5_1ss17GC | Hmo-MIR5141-p5 | CAGCATCAAGGTCTGACCCAAA | 22 | 154 | 50.00 | -35.80 | 0.00 | 0.00 | 0.50 |
| bdi-miR5175b_L-2_1ss15TA | Hmo-miR5175 | TCTGTTCCTAAAATCTTGT | 19 | 136 | 36.90 | -58.20 | 0.00 | 0.00 | 0.50 |
| mtr-miR5291a_L-3_1ss22GA | Hmo-miR5291 | TGATGGATGGATTGGATGAAT | 21 | 56 | 30.90 | -22.80 | 0.00 | 0.21 | 0.00 |
| bdi-miR529-5p_R-2_1ss8AG | Hmo-miR529a | AGAAGAGGGAGAGTACAGC | 19 | 107 | 43.90 | -52.70 | 11.87 | 6.09 | 3.82 |
| osa-miR529b_R-1_1ss8AG | Hmo-miR529b | AGAAGAGGGAGAGTACAGCT | 20 | 138 | 45.70 | -59.50 | 1969.65 | 852.54 | 571.58 |
| lus-miR530a_R+1_1ss20TG | Hmo-miR530 | TGCATTTGCACCTGCACCTGA | 21 | 116 | 37.90 | -46.80 | 15.02 | 4.78 | 7.59 |
| sly-miR5300 | Hmo-miR5300 | TCCCCAGTCCAGGCATTCCAAC | 22 | 263 | 35.50 | -98.50 | 0.00 | 0.00 | 0.46 |
| ppe-miR535a | Hmo-miR535 | TGACAACGAGAGAGAGCACGC | 21 | 103 | 50.50 | -63.30 | 3906.91 | 10305.65 | 9059.45 |
| osa-MIR5523-p3 | Hmo-MIR5523 | AAATATGTTGACATGAGGAGGAAC | 24 | 68 | 28.80 | -19.40 | 0.00 | 0.95 | 1.83 |
| bra-miR5654a_R-1 | Hmo-miR5654 | ATAAATCCCAAGCATCATCC | 20 | 190 | 37.90 | -71.80 | 0.00 | 0.21 | 0.00 |
| nta-miR6020b | Hmo-miR6020 | AAATGTTCTTCGAGTATCTTC | 21 | 109 | 32.50 | -45.50 | 4.92 | 0.21 | 1.42 |
| nta-miR6021 | Hmo-miR6021 | TTGGAAGAGGCTGCTATTGGA | 21 | 156 | 47.00 | -39.90 | 0.00 | 0.37 | 0.50 |
| nta-miR6025a | Hmo-miR6025a | TACCAACAATTGAGATAACATC | 22 | 145 | 36.40 | -66 | 7.12 | 0.21 | 1.97 |
| nta-miR6025c | Hmo-miR6025b | TCAATTGAGATGACATCTAGT | 21 | 155 | 38.40 | -76 | 3.97 | 0.00 | 0.00 |
| bna-miR6030 | Hmo-miR6030 | TCCACCCATACCATACAGACCC | 22 | 108 | 46.00 | -55.60 | 0.00 | 0.21 | 0.00 |
| nta-miR6144 | Hmo-miR6144 | TGGCAACTTCTTCATCATGCC | 21 | 132 | 39.70 | -48.20 | 3.45 | 0.00 | 0.00 |
| nta-miR6145e_R-3 | Hmo-miR6145a | ATTGTTACATGTAGCACT | 18 | 74 | 46.20 | -31 | 0.99 | 0.00 | 0.00 |
| nta-miR6145b | Hmo-miR6145b | TTATCATACGTAGCACTAGCC | 21 | 76 | 37.50 | -32.60 | 0.00 | 0.00 | 0.46 |
| nta-miR6145f_L+1 | Hmo-miR6145c | TATCGTAACATATAGCACTAGC | 22 | 78 | 40.70 | -37 | 0.99 | 0.00 | 0.00 |
| nta-miR6146b | Hmo-miR6146 | TTTGTCCAATGAAATACTTATC | 22 | 73 | 39.50 | -34.40 | 1.98 | 0.00 | 0.00 |
| nta-miR6147 | Hmo-miR6147 | TGACATCTTCAAAACCCACTA | 21 | 102 | 34.60 | -44.40 | 0.99 | 0.21 | 0.96 |
| nta-miR6149a | Hmo-miR6149a | TTGATACGCACCTGAATCGGC | 21 | 97 | 38.40 | -38.60 | 38.46 | 6.63 | 10.21 |
| stu-miR6149-3p_L-1R-1_1ss15AT | Hmo-miR6149b | GATTCAGGTTTGTTTGCAAA | 20 | 65 | 28.40 | -26.10 | 0.00 | 0.00 | 0.50 |
| nta-miR6155 | Hmo-miR6155 | TAAGGTTGCCTTGCTCTTGCA | 21 | 120 | 41.80 | -61.30 | 1.98 | 0.00 | 0.00 |
| nta-miR6161c | Hmo-miR6161 | AATATACTGGAGTTCGGTGCACCT | 24 | 62 | 45.60 | -34 | 0.00 | 0.00 | 0.50 |
| nta-miR6164a_L-1R+4_1ss13AT | Hmo-miR6164 | CACATAAATTGTAACGGAGGGAGT | 24 | 220 | 33.80 | -72.30 | 0.73 | 0.00 | 0.00 |
| gma-miR6300 | Hmo-miR6300 | GTCGTTGTAGTATAGTGG | 18 | 250 | 25.90 | -72 | 2638.45 | 88.23 | 161.37 |
| ptc-miR6476a_L-2 | Hmo-miR6476 | AGTGGAGATGAAACATGA | 18 | 95 | 37.40 | -23.20 | 0.00 | 0.21 | 0.00 |
| mdm-miR7121a | Hmo-miR7121 | TCCTCTTGGTGATCGCCCTGT | 21 | 132 | 47.00 | -50.50 | 0.00 | 1.38 | 0.00 |
| mdm-miR7122a | Hmo-miR7122a | TTATACAGAGAAATCACGGTCG | 22 | 122 | 41.80 | -45.30 | 3.19 | 0.79 | 1.51 |
| stu-miR7122-5p_1ss18TG | Hmo-miR7122b | TTATACAGAGAAACCGCGGTCG | 22 | 75 | 40.30 | -31.70 | 0.73 | 0.58 | 0.50 |
| bdi-miR7782-3p_R-5_1ss4TA | Hmo-miR7782 | ACCAGCTCTGATACCATGT | 19 | 127 | 40.00 | -55.90 | 0.00 | 1.36 | 0.50 |
| rgl-miR7972_1ss14TG | Hmo-miR7972 | TTGTCAGGCTTGTGATTCTCC | 21 | 192 | 41.50 | -66.70 | 1.73 | 0.64 | 1.97 |
| stu-miR8036-3p_R-1_1ss2AT | Hmo-miR8036 | TTTGTCTTTCCGATGCCTCCC | 21 | 90 | 28.30 | -31.20 | 0.73 | 0.00 | 1.01 |
| stu-miR8051-5p_L-1_1ss4TC | Hmo-miR8051 | AGCATGGTAGAAAGATTCA | 19 | 41 | 27.90 | -17.90 | 0.99 | 0.00 | 0.00 |
| ath-miR8175_L+4 | Hmo-miR8175 | GTTCGATCCCCGGCAACGGCGCCA | 24 | 189 | 50.30 | -127.60 | 32.54 | 6.85 | 4.35 |
| bdi-miR827-3p | Hmo-miR827 | TTAGATGACCATCAGCAAACA | 21 | 160 | 49.40 | -66.50 | 6.91 | 8.53 | 4.76 |
| mdm-miR828a_1ss22AT | Hmo-miR828a | TCTTGCTCAAATGAGTATTCCT | 22 | 142 | 39.40 | -66.70 | 0.00 | 4.01 | 5.58 |
| aly-miR828-3p_1ss9AC | Hmo-miR828b | AGATGCTCCTTTGAGCAAGCAA | 22 | 142 | 39.40 | -66.70 | 0.21 | 1.43 | 1.63 |
| mdm-miR858 | Hmo-miR858 | TTCGTTGTCTGTTCGACCTGA | 21 | 921 | 35.10 | -242.60 | 0.00 | 3.06 | 0.50 |
| tae-miR9773_R-6_1ss2TC | Hmo-miR9773 | TCTGTTTTTATGTTATTT | 18 | 162 | 26.70 | -30.10 | 0.00 | 0.21 | 0.00 |
| PC-3p-193959_6 | Hmo-novel-1 | TTCCAGAAAGCTGTGGGAAG | 20 | 120 | 35.70 | -55 | 5.18 | 6.66 | 8.24 |
| PC-5p-192_7269 | Hmo-novel-2 | CAGCTTTCTTGAACTTTCCCC | 21 | 104 | 39.60 | -61.60 | 566.21 | 500.93 | 773.07 |
| PC-3p-743880_2 | Hmo-novel-3 | TGGGAAAGTTCAAGAAAGCTGT | 22 | 104 | 39.60 | -61.60 | 0.73 | 1.06 | 0.23 |
| PC-5p-108182_10 | Hmo-novel-4 | ATTAAGTGGGTTTGGGTAAAGATT | 24 | 248 | 42.90 | -186 | 25.23 | 29.70 | 24.30 |
| PC-3p-73758_14 | Hmo-novel-5 | ATTAAACGGGTTAATCGTGTCAAC | 24 | 248 | 42.90 | -186 | 4.42 | 6.53 | 3.11 |
| PC-5p-2548_357 | Hmo-novel-6 | ACCCGACACGATTACCGACCCGAA | 24 | 236 | 43.30 | -168.70 | 641.43 | 812.61 | 744.14 |
| PC-5p-2975_303 | Hmo-novel-7 | TTACTTGGCACTTACGACAGA | 21 | 157 | 40.40 | -90.80 | 29.05 | 62.69 | 58.60 |
| PC-3p-128415_8 | Hmo-novel-8 | TCATAAGTGCCAAGTACCTAG | 21 | 157 | 40.40 | -90.80 | 5.70 | 3.70 | 1.97 |
| PC-5p-118896_9 | Hmo-novel-9 | ATTCGGTCTTCGGTCCGGTCCGGT | 24 | 69 | 31.40 | -63.60 | 19.94 | 13.57 | 10.58 |
| PC-3p-4085574_1 | Hmo-novel-10 | AATATTGATTTGTTTTTCATGGTG | 24 | 69 | 31.40 | -63.60 | 0.73 | 0.79 | 0.46 |
| PC-5p-39655_24 | Hmo-novel-11 | TTGCTTATACTTTCTCTTACC | 21 | 95 | 39.60 | -46.80 | 2.20 | 5.26 | 5.86 |
| PC-3p-53338_18 | Hmo-novel-12 | AGAGAAAGCATAAGCAACTGT | 21 | 95 | 39.60 | -46.80 | 0.00 | 7.62 | 6.07 |
| PC-5p-67683_15 | Hmo-novel-13 | TTGCGATAAGCATATGACTTC | 21 | 305 | 39.00 | -134.90 | 3.71 | 6.26 | 2.84 |
| PC-3p-1321295_2 | Hmo-novel-14 | TAAGATCCATTCGTACAGCTAG | 22 | 305 | 39.00 | -134.90 | 0.00 | 0.79 | 0.91 |
| PC-5p-23845_39 | Hmo-novel-15 | TCGCGCCTCGGGACCCTTTGC | 21 | 196 | 51.70 | -206.30 | 48.30 | 30.55 | 32.10 |
| PC-3p-313265_4 | Hmo-novel-16 | CTCAGTGCGCCTTGCTCTTTT | 21 | 196 | 51.70 | -206.30 | 1.73 | 2.81 | 1.42 |
| PC-5p-5445_159 | Hmo-novel-17 | ATTCCATTACCATGTTTGGTTACC | 24 | 61 | 31.00 | -45.40 | 93.20 | 90.84 | 43.25 |
| PC-3p-69283_14 | Hmo-novel-18 | AATTACAATTACTTGACTTTTCCT | 24 | 61 | 31.00 | -45.40 | 16.27 | 21.26 | 8.05 |
| PC-5p-935900_2 | Hmo-novel-19 | AGGCGGAGGTGGAGGAGTGGT | 21 | 132 | 60.70 | -95.10 | 0.73 | 0.00 | 1.42 |
| PC-3p-17334_53 | Hmo-novel-20 | CACTCCTCCGCCACCGCCTCC | 21 | 132 | 60.70 | -95.10 | 24.43 | 9.02 | 8.65 |
| PC-5p-1835_491 | Hmo-novel-21 | ACGCCTAATGCTGTGTATGGGAGG | 24 | 241 | 36.60 | -151.90 | 178.78 | 221.71 | 117.73 |
| PC-3p-3086_289 | Hmo-novel-22 | CAAGGAGTCGAATACGCAACCTCT | 24 | 241 | 36.60 | -151.90 | 181.46 | 224.38 | 164.12 |
| PC-5p-29055_32 | Hmo-novel-23 | TTAGTTTGAAAAAGCGCGAGGC | 22 | 196 | 49.90 | -205.20 | 4.94 | 5.87 | 5.79 |
| PC-3p-12531_71 | Hmo-novel-24 | CGCACTGAGGCGCAAAGGGTC | 21 | 196 | 49.90 | -205.20 | 37.33 | 47.59 | 48.72 |
| PC-5p-14322_63 | Hmo-novel-25 | CTAATGTCTTCTGTTTTCATG | 21 | 166 | 37.50 | -66.90 | 8.16 | 8.65 | 15.48 |
| PC-3p-86218_12 | Hmo-novel-26 | AAACAGACAACATTAGAGGAT | 21 | 166 | 37.50 | -66.90 | 0.00 | 2.11 | 5.91 |
| PC-5p-672176_2 | Hmo-novel-27 | AAATATTTTGCTAAATTGGTGAACT | 25 | 297 | 33.60 | -152.70 | 1.47 | 0.43 | 0.50 |
| PC-3p-53677_18 | Hmo-novel-28 | ACAATCTTTGACATTAGACAGAGA | 24 | 297 | 33.60 | -152.70 | 7.38 | 14.13 | 13.73 |
| PC-5p-548980_3 | Hmo-novel-29 | CTATTTTTATTTTTCCACAGC | 21 | 152 | 40.60 | -99.80 | 3.93 | 2.85 | 2.79 |
| PC-3p-62943_16 | Hmo-novel-30 | TGTGGGAAAATACAGATAGGA | 21 | 152 | 40.60 | -99.80 | 2.94 | 9.21 | 4.35 |
| PC-5p-1435702_2 | Hmo-novel-31 | CAATCTGACCTTTGACCCTATTT | 23 | 167 | 30.50 | -65.70 | 1.98 | 2.27 | 1.88 |
| PC-3p-879592_2 | Hmo-novel-32 | ATGTTAGACTGTTTGCTGTAA | 21 | 167 | 30.50 | -65.70 | 0.00 | 1.10 | 0.50 |
| PC-5p-1177652_2 | Hmo-novel-33 | ATACACCTGAGAGTATGTACTCCT | 24 | 143 | 34.90 | -50 | 2.94 | 3.24 | 2.38 |
| PC-3p-4296098_1 | Hmo-novel-34 | AGAGTACATACTCCCAGGTGTATG | 24 | 143 | 34.90 | -50 | 0.00 | 1.38 | 0.46 |
| PC-5p-434063_3 | Hmo-novel-35 | ATATAAGAGGGTGTGTACCAT | 21 | 167 | 29.50 | -77.40 | 3.19 | 3.12 | 1.47 |
| PC-3p-5225013_1 | Hmo-novel-36 | CATACGTTTGTATCTAAGAATTT | 23 | 167 | 29.50 | -77.40 | 0.73 | 1.01 | 0.00 |
| PC-5p-40790_23 | Hmo-novel-37 | CGCCTCGCGCTTTTTCAAACT | 21 | 180 | 48.50 | -214 | 22.92 | 17.62 | 14.00 |
| PC-3p-437108_3 | Hmo-novel-38 | CAGGATAAGGATAAAGGAGAATT | 23 | 180 | 48.50 | -214 | 0.99 | 2.17 | 1.51 |
| PC-5p-10950_81 | Hmo-novel-39 | TAGGGTCAAAGGTCAGATTGT | 21 | 231 | 33.40 | -108.60 | 10.83 | 24.18 | 29.06 |
| PC-3p-13164_68 | Hmo-novel-40 | TCAAAGGTCAGATTGTTTGCT | 21 | 231 | 33.40 | -108.60 | 24.82 | 34.12 | 47.38 |
| PC-3p-99134_10 | Hmo-novel-41 | CAGGGCGATCAGGGTAGCTCCT | 22 | 102 | 55.00 | -59 | 13.85 | 11.25 | 16.44 |
