# Supplementary material for: Integrated sRNAome and RNA-Seq analysis reveals miRNA effects on betalain biosynthesis in pitaya
Source: BMC Plant Biol. 2020 Sep 22;20:437. doi: 10.1186/s12870-020-02622-x (PMC7510087; doi:10.1186/s12870-020-02622-x)
Supplement: Supplementary file 10 — Additional file 10: Table S3. Statistics of gene annotation in six databases. [file 12870_2020_2622_MOESM10_ESM.docx]

**TABLE S3 Statistics of gene annotation in six databases**

| gene_number | Swiss-Prot | nr | Pfam | KEGG | KOG | GO |
| --- | --- | --- | --- | --- | --- | --- |
| 39,737 | 14,306 | 21,783 | 17,106 | 7,966 | 19,121 | 12,559 |
| 100% | 36.0% | 54.8% | 43.1% | 20.1% | 48.1% | 31.6% |
