# Supplementary material for: Integrated sRNAome and RNA-Seq analysis reveals miRNA effects on betalain biosynthesis in pitaya
Source: BMC Plant Biol. 2020 Sep 22;20:437. doi: 10.1186/s12870-020-02622-x (PMC7510087; doi:10.1186/s12870-020-02622-x)
Supplement: Supplementary file 11 — Additional file 11: Table S4. Sequences of miRNA-specific primers for reverse transcription. [file 12870_2020_2622_MOESM11_ESM.docx]

**TABLE S4 Sequences of miRNA-specific primers for reverse transcription**

| miRNA names | Primers for reverse transcription (5′ to 3′) |
| --- | --- |
| Hmo-novel-2 | GTCGTATCCAGTGCAGGGTCCGAGGTATTCGACTGGATACGACGGGGAA |
| Hmo-novel-7 | GTCGTATCCAGTGCAGGGTCCGAGGTATTCGACTGGATACGACTCTGTC |
| Hmo-novel-12 | GTCGTATCCAGTGCAGGGTCCGAGGTATTCGACTGGATACGACACAGTT |
| Hmo-novel-15 | GTCGTATCCAGTGCAGGGTCCGAGGTATTCGACTGGATACGACGCAAAG |
| Hmo-novel-21 | GTCGTATCCAGTGCAGGGTCCGAGGTATTCGACTGGATACGACCCTCCC |
| Hmo-miR156 | GTCGTATCCAGTGCAGGGTCCGAGGTATTCGACTGGATACGACGTGCTC |
| Hmo-miR157b | GTCGTATCCAGTGCAGGGTCCGAGGTATTCGACTGGATACGACGTGCTC |
| Hmo-miR159a | GTCGTATCCAGTGCAGGGTCCGAGGTATTCGACTGGATACGACTAGAGC |
| Hmo-miR159c | GTCGTATCCAGTGCAGGGTCCGAGGTATTCGACTGGATACGACATTGGA |
| Hmo-miR160a | GTCGTATCCAGTGCAGGGTCCGAGGTATTCGACTGGATACGACTTGCCT |
| Hmo-miR160b | GTCGTATCCAGTGCAGGGTCCGAGGTATTCGACTGGATACGACCGGCAT |
| Hmo-miR164a | GTCGTATCCAGTGCAGGGTCCGAGGTATTCGACTGGATACGACTGCACG |
| Hmo-miR164b | GTCGTATCCAGTGCAGGGTCCGAGGTATTCGACTGGATACGACGATGGG |
| Hmo-miR171c | GTCGTATCCAGTGCAGGGTCCGAGGTATTCGACTGGATACGACGGTGAT |
| Hmo-miR171d | GTCGTATCCAGTGCAGGGTCCGAGGTATTCGACTGGATACGACTGGGAT |
| Hmo-miR172a | GTCGTATCCAGTGCAGGGTCCGAGGTATTCGACTGGATACGACATGCAG |
| Hmo-miR390a | GTCGTATCCAGTGCAGGGTCCGAGGTATTCGACTGGATACGACTGAAAC |
| Hmo-miR390b | GTCGTATCCAGTGCAGGGTCCGAGGTATTCGACTGGATACGACGGCGCT |
| Hmo-miR393 | GTCGTATCCAGTGCAGGGTCCGAGGTATTCGACTGGATACGACGGATCA |
| Hmo-miR394 | GTCGTATCCAGTGCAGGGTCCGAGGTATTCGACTGGATACGACGGAGGT |
| Hmo-miR396b | GTCGTATCCAGTGCAGGGTCCGAGGTATTCGACTGGATACGACTCCCAC |
| Hmo-miR397b | GTCGTATCCAGTGCAGGGTCCGAGGTATTCGACTGGATACGACATTTCA |
| Hmo-miR398a | GTCGTATCCAGTGCAGGGTCCGAGGTATTCGACTGGATACGACAAGGGG |
| Hmo-miR398b | GTCGTATCCAGTGCAGGGTCCGAGGTATTCGACTGGATACGACGGGGCG |
| Hmo-miR399a | GTCGTATCCAGTGCAGGGTCCGAGGTATTCGACTGGATACGACAAGGGC |
| Hmo-miR408 | GTCGTATCCAGTGCAGGGTCCGAGGTATTCGACTGGATACGACGCCAGG |
| Hmo-miR529b | GTCGTATCCAGTGCAGGGTCCGAGGTATTCGACTGGATACGACAGCTGT |
| Hmo-miR530 | GTCGTATCCAGTGCAGGGTCCGAGGTATTCGACTGGATACGACTCAGGT |
| Hmo-miR535 | GTCGTATCCAGTGCAGGGTCCGAGGTATTCGACTGGATACGACGCGTGC |
| Hmo-miR828a | GTCGTATCCAGTGCAGGGTCCGAGGTATTCGACTGGATACGACAGGAAT |
| Hmo-miR858 | GTCGTATCCAGTGCAGGGTCCGAGGTATTCGACTGGATACGACTCAGGT |
| Hmo-miR5072 | GTCGTATCCAGTGCAGGGTCCGAGGTATTCGACTGGATACGACTGGCGA |
| Hmo-miR6020 | GTCGTATCCAGTGCAGGGTCCGAGGTATTCGACTGGATACGACGAAGAT |
| Hmo-miR6300 | GTCGTATCCAGTGCAGGGTCCGAGGTATTCGACTGGATACGACCCACTA |
| U6 | GTCGTATCCAGTGCAGGGTCCGAGGTATTCGACTGGATACGACCATTTC |
