# Supplementary material for: Integrated sRNAome and RNA-Seq analysis reveals miRNA effects on betalain biosynthesis in pitaya
Source: BMC Plant Biol. 2020 Sep 22;20:437. doi: 10.1186/s12870-020-02622-x (PMC7510087; doi:10.1186/s12870-020-02622-x)
Supplement: Supplementary file 12 — Additional file 12: Table S5. Sequences of miRNA primers for real-time PCR. [file 12870_2020_2622_MOESM12_ESM.docx]

**TABLE S5 Sequences of miRNA primers for real-time PCR**

| miRNA names | miRNA primers for real-time PCR |
| --- | --- |
| Hmo-novel-2-F | CAGCTTTCTTGAACTTTCCCC |
| Hmo-novel-7-F | AGCGCGTTACTTGGCACTTAC |
| Hmo-novel-12-F | AGCGCGAGAGAAAGCATAAGC |
| Hmo-novel-15-F | ATATATCGCGCCTCGGGACCCT |
| Hmo-novel-21-F | ACGCGACGCCTAATGCTGTGT |
| Hmo-miR156-F | AGCGCGTTGCCAGAAGAGAGT |
| Hmo-miR157b-F | AGCGCGCTGCCAGAAGATAGA |
| Hmo-miR159a-F | AGCGCGTTTGGATTGAAGGGA |
| Hmo-miR159c-F | ATATGCGCAGCTCCCTTCGG |
| Hmo-miR160a-F | AGCACGACATACAGGGAGCCA |
| Hmo-miR160b-F | ATATACTGCCTGGCTCCCTGT |
| Hmo-miR164a-F | ATATATGGAGAAGCAGGGCACG |
| Hmo-miR164b-F | ATCGCGCATGTGCCTGTCTTC |
| Hmo-miR171c-F | ACTTATCTTGAGCCGCGCCA |
| Hmo-miR171d-F | ATATCGCTTGAGCCGTGCCA |
| Hmo-miR172a-F | AGCGCGTCCGAATCTTGATGA |
| Hmo-miR390a-F | AGCGCCGCTATCCATCCTGA |
| Hmo-miR390b-F | ACGCAAGCTCAGGAGGGATAG |
| Hmo-miR393-F | AAGGGCTCCAAAGGGATCGCA |
| Hmo-miR394-F | AGCGCATGGGCATTCTGTCC |
| Hmo-miR396b-F | ACGCGCCGGTTCAATAAAGCT |
| Hmo-miR397b-F | AGCGCTTGAGTGCAGCGTTG |
| Hmo-miR398a-F | AGCGCTGTGTTCTCAGGTCA |
| Hmo-miR398b-F | ATAGCTGTGTTCTCAGGTCGC |
| Hmo-miR399a-F | ATAGCGCGCCAAAGGAGAGTT |
| Hmo-miR408-F | AGCGCTTGCACTGCCTCTTC |
| Hmo-miR529b-F | AGCGCGAGAAGAGGGAGAGTA |
| Hmo-miR530-F | ACGGGATGCATTTGCACCTG |
| Hmo-miR535-F | AGCGCGTGACAACGAGAGAGA |
| Hmo-miR828a-F | AGCGCGTCTTGCTCAAATGAGT |
| Hmo-miR858-F | AGCGCGTTCGTTGTCTGTTCG |
| Hmo-miR5072-F | TCCCCAGTGGAGTCGCCA |
| Hmo-miR6020-F | AGCGCGGTTCTTCGAGTATCTTC |
| Hmo-miR6300-F | AGCGCGGTCGTTGTAGTATAG |
| U6-F | AGCGCCGATAAAATTGGAACGATAC |
| ALL-R | ACAGTGCAGGGTCCGAGGTATT |
