# Supplementary material for: Integrated sRNAome and RNA-Seq analysis reveals miRNA effects on betalain biosynthesis in pitaya
Source: BMC Plant Biol. 2020 Sep 22;20:437. doi: 10.1186/s12870-020-02622-x (PMC7510087; doi:10.1186/s12870-020-02622-x)
Supplement: Supplementary file 13 — Additional file 13: Table S6. Sequences of target genes primers for real-time PCR. [file 12870_2020_2622_MOESM13_ESM.docx]

**TABLE S6 Sequences of target genes primers for real-time PCR**

| Names of target genes | Primers of target genes for real-time PCR |
| --- | --- |
| *comp24967_c0*-F | CAGGAAGAACGGACAACGAG |
| *comp24967_c0*-R | GAGCAACAGGGATAGGGACA |
| *comp234190_c0*-F | AGTTTCGTGGTGCTCTTGAA |
| *comp234190_c0*-R | CCATTACCTTGGGTATTTGG |
| *comp29967_c0*-F | TGTAGCAGGAACTGATACAA |
| *comp29967_c0*-R | CCCGAACAACCAATAATGGA |
| *comp24676_c0*-F | ATAGTCGAATCAAAGCCCAA |
| *comp24676_c0*-R | TCCCGAAGCTCACATAAAGG |
| *comp15143_c0*-F | GGTCTTTAATAGCTGGGAGG |
| *comp15143_c0*-R | TCTGTTGTTGCTTAGTGGTG |
| *comp24362_c0*-F | ATTCGTGGTTATTTTTGATG |
| *comp24362_c0*-R | TACTTGTGGTAGTGGCTGTG |
| *comp403340_c0*-F | TCTCATCATTCATCTCCACC |
| *comp403340_c0*-R | TCCCTTGCTTTATTAGCTTC |
| *comp15849_c0*-F | AAGATTACCAGGAAGGACAG |
| *comp15849_c0*-R | GTTCTAAGGCAAGGAAGATG |
| *comp36993_c0*-F | TCGGGAGCATCACCACAATA |
| *comp36993_c0*-R | CAGAGCGCCCCTTTACAAAC |
| *comp35191_c0*-F | GCCAAAGAAATGTTCCAAAA |
| *comp35191_c0*-R | GACTGCCTAGCATCGAGTCG |
| *comp28219_c0*-F | AATGTAAAAAATGTAGGGCT |
| *comp28219_c0*-R | ACTTAGTTTGAAAAAGAGCG |
| *comp25631_c0*-F | GTATGGGGATGGCCGGGTTT |
| *comp25631_c0*-R | CTGCATGGCTGCCTTTGTGG |
| *comp27657_c0*-F | GCTACAAAAGTGTAGAAAGC |
| *comp27657_c0*-R | TCAGACATATAGAAGAGTGC |
| *comp26829_c0*-F | ATGGATTTTTGCCAGTGATT |
| *comp26829_c0*-R | CTGCCCTCTATTGGATGTTG |
| *comp25650_c0*-F | AGCAGCAGTAGCAGGAATGT |
| *comp25650_c0*-R | GGTTGTTGATGATGGGTCGG |
| *comp27464_c0*-F | TTGACACGCCATTATGCAAC |
| *comp27464_c0*-R | CACAATCATCTCCTCTCCGA |
| *comp6695_c0*-F | CAACACCCAAAACAACCTCA |
| *comp6695_c0*-R | GCTTCCTCCCCAGTCACTAT |
