# Supplementary material for: Integrated sRNAome and RNA-Seq analysis reveals miRNA effects on betalain biosynthesis in pitaya
Source: BMC Plant Biol. 2020 Sep 22;20:437. doi: 10.1186/s12870-020-02622-x (PMC7510087; doi:10.1186/s12870-020-02622-x)
Supplement: Supplementary file 14 — Additional file 14: Table S7. Sequences of specific primers for 5′RACE. [file 12870_2020_2622_MOESM14_ESM.docx]

**TABLE S7 Sequences of specific primers for** **5****′RACE**

| Genes names | Specific primers for nested PCR |
| --- | --- |
| *comp24967_c0*-outer | CGGCTTGACATTTATTGACGCAGGT |
| *comp24967_c0*-inner | GATTACGCCAAGCTTACCGTGGTCCCAATTGTCATCCATT |
| *comp29967_c0*-outer | TTTTGTGCATTGTGATTCCAGGCAA |
| *comp29967_c0*-inner | GATTACGCCAAGCTTTCTTCCTCCGTCATTCCAACTGGCA |
| *comp15143_c0*-outer | CGCCAAGCCAATCTTCAAGCATCTC |
| *comp15143_c0*-inner | GATTACGCCAAGCTTGGGATGATATTCTGGGGAATTGGGA |
| *comp24362_c0*-outer | TTGCTGGTTGTTCTCCAAGCCAATC |
| *comp24362_c0*-inner | GATTACGCCAAGCTTCGGGGATAAATCATTGTCACCACCG |
| *comp36993_c0*-outer | TCTCACCCCTTGCAAATCCAAAGGC |
| *comp36993_c0*-inner | GATTACGCCAAGCTTGCAAATCCAAAGGCCTAAGCAATGG |
| *comp28219_c0*-outer | AGGATGCACCAAAAACCTGGCAGCT |
| *comp28219_c0*-inner | GATTACGCCAAGCTTTCCTCTGGCAGCTTTGTCATCATGC |
| *comp25631_c0*-outer | GGATCTCCTGAATCTCGCAAAAGCA |
| *comp25631_c0*-inner | GATTACGCCAAGCTTAGGATGATGATGCCCACAGTGACGC |
