# Supplementary material for: Integrated sRNAome and RNA-Seq analysis reveals miRNA effects on betalain biosynthesis in pitaya
Source: BMC Plant Biol. 2020 Sep 22;20:437. doi: 10.1186/s12870-020-02622-x (PMC7510087; doi:10.1186/s12870-020-02622-x)
Supplement: Supplementary file 15 — Additional file 15: Table S8. The information of part RNA-Seq data in pitaya. [file 12870_2020_2622_MOESM15_ESM.docx]

**TABLE S8** **The information of part RNA-Seq data in pitaya**

| Gene_ID | Length | Names | Annotation | Hp19d_rpkm | Hp25d_rpkm | Hp29d_rpkm |
| --- | --- | --- | --- | --- | --- | --- |
| comp24967_c0 | 1506 | TT2 | myb proto-oncogene protein, plant | 3.89 | 132.91 | 67.84 |
| comp234190_c0 | 847 | CYP71A8 | - | 5.62 | 0.10 | 0.32 |
| comp29967_c0 | 1725 | CYP83B1 | cytochrome P450, family 83, subfamily B, polypeptide 1 | 78.16 | 83.52 | 97.91 |
| comp24676_c0 | 1202 | GmSGT2 | coniferyl-alcohol glucosyltransferase | 2.95 | 2.26 | 0.90 |
| comp15143_c0 | 888 | MYB12 | myb proto-oncogene protein, plant | 6.76 | 2.06 | 0.69 |
| comp24362_c0 | 1232 | C1 | myb proto-oncogene protein, plant | 25.46 | 8.97 | 18.44 |
| comp403340_c0 | 241 | MYB2 | myb proto-oncogene protein, plant | 4.94 | 0.00 | 0.00 |
| comp15849_c0 | 217 | MYB315 | myb proto-oncogene protein, plant | 0.00 | 1.49 | 8.74 |
| comp28219_c0 | 3287 | TPST | - | 81.30 | 71.27 | 59.60 |
| comp25631_c0 | 2183 | SPL6 | - | 12.75 | 7.38 | 3.91 |
| comp27657_c0 | 2848 | WDTC1 | WD and tetratricopeptide repeats protein 1 | 22.83 | 20.46 | 14.51 |
| comp26829_c0 | 3614 | BHLH155 | - | 31.86 | 20.50 | 22.21 |
| comp25650_c0 | 1967 | SPL16 | - | 45.75 | 1.01 | 1.36 |
| comp27464_c0 | 3704 | SEC | polypeptide N-acetylglucosaminyltransferase | 64.34 | 93.95 | 77.28 |
| comp6695_c0 | 906 | MYB330 | myb proto-oncogene protein, plant | 6.71 | 0.60 | 3.15 |
