# Supplementary material for: Integrated sRNAome and RNA-Seq analysis reveals miRNA effects on betalain biosynthesis in pitaya
Source: BMC Plant Biol. 2020 Sep 22;20:437. doi: 10.1186/s12870-020-02622-x (PMC7510087; doi:10.1186/s12870-020-02622-x)
Supplement: Supplementary file 16 — Additional file 16: Table S9. Prediction of targets for differentially expression miRNAs in pitaya. [file 12870_2020_2622_MOESM16_ESM.docx]

**TABLE S9** **Prediction of targets for differentially expression miRNAs in pitaya**

| miRNA names | Gene ID | Score | Gene annotation |
| --- | --- | --- | --- |
| Hmo-novel-12 | comp24432_c0 | 3 | - |
| Hmo-novel-12 | comp22542_c1 | 3 | - |
| Hmo-novel-12 | comp29866_c0 | 3 | sp\|Q9SLF3\|TC132_ARATH Translocase of chloroplast 132, chloroplastic OS=Arabidopsis thaliana GN=TOC132 PE=1 SV=1 |
| Hmo-novel-12 | comp264497_c0 | 4 | - |
| Hmo-novel-12 | comp403295_c0 | 4 | - |
| Hmo-novel-12 | comp22782_c0 | 4 | sp\|Q551H4\|FRAY2_DICDI Serine/threonine-protein kinase fray2 OS=Dictyostelium discoideum GN=fray2 PE=3 SV=1 |
| Hmo-novel-12 | comp25869_c0 | 4 | sp\|Q6R3K6\|YSL6_ARATH Probable metal-nicotianamine transporter YSL6 OS=Arabidopsis thaliana GN=YSL6 PE=2 SV=2 |
| Hmo-novel-21 | comp29758_c0 | 0 | - |
| Hmo-novel-2 | comp802_c0 | 1 | - |
| Hmo-novel-2 | comp25800_c0 | 3 | sp\|P93026\|VSR1_ARATH Vacuolar-sorting receptor 1 OS=Arabidopsis thaliana GN=VSR1 PE=1 SV=2 |
| Hmo-novel-2 | comp30009_c0 | 3.5 | sp\|Q9SV46\|PP282_ARATH Pentatricopeptide repeat-containing protein At3g54980, mitochondrial OS=Arabidopsis thaliana GN=At3g54980 PE=2 SV=1 |
| Hmo-novel-2 | comp20052_c0 | 3.5 | sp\|Q6AWX7\|GRF12_ORYSJ Growth-regulating factor 12 OS=Oryza sativa subsp. japonica GN=GRF12 PE=2 SV=1 |
| Hmo-novel-2 | comp29780_c0 | 4 | sp\|Q8RYD3\|Y3158_ARATH B3 domain-containing protein At3g11580 OS=Arabidopsis thaliana GN=ARF32 PE=2 SV=1 |
| Hmo-novel-2 | comp26546_c0 | 4 | sp\|Q6P0Q8\|MAST2_HUMAN Microtubule-associated serine/threonine-protein kinase 2 OS=Homo sapiens GN=MAST2 PE=1 SV=2 |
| Hmo-novel-2 | comp26675_c0 | 4 | - |
| Hmo-novel-2 | comp29967_c0 | 4 | sp\|O65782\|C83B1_ARATH Cytochrome P450 83B1 OS=Arabidopsis thaliana GN=CYP83B1 PE=1 SV=1 |
| Hmo-novel-2 | comp15700_c0 | 4 | sp\|Q0WVK7\|PPR12_ARATH Pentatricopeptide repeat-containing protein At1g05670, mitochondrial OS=Arabidopsis thaliana GN=At1g05670 PE=2 SV=1 |
| Hmo-novel-15 | comp12607_c0 | 0 | - |
| Hmo-novel-15 | comp417126_c0 | 3 | - |
| Hmo-novel-15 | comp28219_c0 | 3 | sp\|Q3EDG5\|TPST_ARATH Protein-tyrosine sulfotransferase OS=Arabidopsis thaliana GN=TPST PE=1 SV=3 |
| Hmo-novel-15 | comp23343_c0 | 4 | - |
| Hmo-novel-7 | comp23458_c0 | 2.5 | sp\|Q9C6S1\|FH14_ARATH Formin-like protein 14 OS=Arabidopsis thaliana GN=FH14 PE=3 SV=3 |
| Hmo-miR157b | comp15107_c0 | 4 | - |
| Hmo-miR157b | comp1672_c0 | 4 | - |
| Hmo-miR157b | comp25631_c0 | 4 | sp\|Q94JW8\|SPL6_ARATH Squamosa promoter-binding-like protein 6 OS=Arabidopsis thaliana GN=SPL6 PE=2 SV=2 |
| Hmo-miR157b | comp27920_c0 | 4 | - |
| Hmo-miR157b | comp28753_c0 | 3 | - |
| Hmo-miR390a | comp11005_c0 | 4 | sp\|P74516\|TRML_SYNY3 Putative tRNA (cytidine(34)-2'-O)-methyltransferase OS=Synechocystis sp. (strain PCC 680/Kazusa) GN=slr0992 PE=3 SV=1 |
| Hmo-miR390a | comp25133_c0 | 4 | sp\|Q17RB8\|LONF1_HUMAN LON peptidase N-terminal domain and RING finger protein 1 OS=Homo sapiens GN=LONRF1 PE=2 SV=2 |
| Hmo-miR390b | comp21723_c0 | 3.5 | sp\|O04567\|Y1719_ARATH Probable inactive receptor kinase At1g27190 OS=Arabidopsis thaliana GN=At1g27190 PE=1 SV=1 |
| Hmo-miR390b | comp25044_c0 | 4 | sp\|P49299\|CYSZ_CUCMA Citrate synthase, glyoxysomal OS=Cucurbita maxima PE=1 SV=1 |
| Hmo-miR390b | comp28094_c0 | 4 | sp\|O82318\|Y2579_ARATH Probably inactive leucine-rich repeat receptor-like protein kinase At2g25790 OS=Arabidopsis thaliana GN=At2g25790 PE=1 SV=1 |
| Hmo-miR390b | comp30090_c0 | 4 | sp\|C0LGD7\|Y1684_ARATH Probable LRR receptor-like serine/threonine-protein kinase At1g06840 OS=Arabidopsis thaliana GN=At1g06840 PE=1 SV=2 |
| Hmo-miR390b | comp30282_c0 | 4 | sp\|Q5C9Z4\|NOM1_HUMAN Nucleolar MIF4G domain-containing protein 1 OS=Homo sapiens GN=NOM1 PE=1 SV=1 |
| Hmo-miR390b | comp8262_c0 | 3.5 | - |
| Hmo-miR398a | comp18572_c0 | 4 | sp\|O80763\|NRX1_ARATH Probable nucleoredoxin 1 OS=Arabidopsis thaliana GN=At1g60420 PE=1 SV=1 |
| Hmo-miR398a | comp18635_c0 | 4 | sp\|P22233\|SODC_SPIOL Superoxide dismutase [Cu-Zn] OS=Spinacia oleracea GN=SODCC PE=2 SV=1 |
| Hmo-miR393 | comp25079_c0 | 2 | sp\|Q570C0\|TIR1_ARATH Protein TRANSPORT INHIBITOR RESPONSE 1 OS=Arabidopsis thaliana GN=TIR1 PE=1 SV=2 |
| Hmo-miR393 | comp25440_c0 | 3 | - |
| Hmo-miR393 | comp27134_c1 | 3 | sp\|Q570C0\|TIR1_ARATH Protein TRANSPORT INHIBITOR RESPONSE 1 OS=Arabidopsis thaliana GN=TIR1 PE=1 SV=2 |
| Hmo-miR171d | comp27398_c0 | 3 | sp\|O81316\|SCL6_ARATH Scarecrow-like protein 6 OS=Arabidopsis thaliana GN=SCL6 PE=1 SV=1 |
| Hmo-miR171d | comp28828_c0 | 4 | sp\|O81316\|SCL6_ARATH Scarecrow-like protein 6 OS=Arabidopsis thaliana GN=SCL6 PE=1 SV=1 |
| Hmo-miR171d | comp29850_c0 | 3.5 | sp\|Q42371\|ERECT_ARATH LRR receptor-like serine/threonine-protein kinase ERECTA OS=Arabidopsis thaliana GN=ERECTA PE=1 SV=1 |
| Hmo-miR171d | comp9827_c1 | 4 | - |
| Hmo-miR172a | comp18526_c0 | 3 | sp\|P47927\|AP2_ARATH Floral homeotic protein APETALA 2 OS=Arabidopsis thaliana GN=AP2 PE=1 SV=1 |
| Hmo-miR172a | comp21311_c0 | 3 | sp\|P41152\|HSF30_SOLPE Heat shock factor protein HSF30 OS=Solanum peruvianum GN=HSF30 PE=2 SV=1 |
| Hmo-miR172a | comp25023_c0 | 2.5 | sp\|P47927\|AP2_ARATH Floral homeotic protein APETALA 2 OS=Arabidopsis thaliana GN=AP2 PE=1 SV=1 |
| Hmo-miR172a | comp25597_c0 | 4 | sp\|Q9SKB3\|PARG1_ARATH Poly(ADP-ribose) glycohydrolase 1 OS=Arabidopsis thaliana GN=PARG1 PE=1 SV=2 |
| Hmo-miR394 | comp23031_c0 | 3 | sp\|Q9FZK1\|FBX6_ARATH F-box only protein 6 OS=Arabidopsis thaliana GN=FBX6 PE=2 SV=1 |
| Hmo-miR394 | comp23473_c0 | 4 | sp\|Q9ZWQ7\|DAD1_CITUN Dolichyl-diphosphooligosaccharide--protein glycosyltransferase subunit DAD1 OS=Citrus unshiu GN=DAD1 PE=3 SV=1 |
| Hmo-miR394 | comp7994_c0 | 4 | sp\|Q9C533\|ZDHC1_ARATH Probable S-acyltransferase At1g69420 OS=Arabidopsis thaliana GN=At1g69420 PE=2 SV=2 |
| Hmo-miR6300 | comp18934_c0 | 2.5 | - |
| Hmo-miR6300 | comp19321_c0 | 4 | - |
| Hmo-miR6300 | comp22070_c0 | 4 | sp\|P55862\|MCM5A_XENLA DNA replication licensing factor mcm5-A OS=Xenopus laevis GN=mcm5-a PE=1 SV=2 |
| Hmo-miR6300 | comp25373_c0 | 4 | sp\|Q9C6D2\|MTK_ARATH Methylthioribose kinase OS=Arabidopsis thaliana GN=MTK PE=1 SV=1 |
| Hmo-miR6300 | comp25452_c0 | 3 | sp\|P41127\|RL131_ARATH 60S ribosomal protein L13-1 OS=Arabidopsis thaliana GN=RPL13B PE=1 SV=1 |
| Hmo-miR6300 | comp27471_c1 | 4 | - |
| Hmo-miR6300 | comp29941_c0 | 4 | - |
| Hmo-miR6300 | comp331090_c0 | 3.5 | - |
| Hmo-miR6300 | comp338839_c0 | 4 | - |
| Hmo-miR397b | comp302849_c0 | 1.5 | sp\|Q5N9X2\|LAC4_ORYSJ Laccase-4 OS=Oryza sativa subsp. japonica GN=LAC4 PE=2 SV=1 |
| Hmo-miR530 | comp27317_c0 | 4 | sp\|Q8RX22\|MIP1_ARATH MND1-interacting protein 1 OS=Arabidopsis thaliana GN=MIP1 PE=1 SV=1 |
| Hmo-miR530 | comp711452_c0 | 4 | - |
| Hmo-miR408 | comp942_c0 | 3.5 | sp\|O80517\|BCB2_ARATH Uclacyanin-2 OS=Arabidopsis thaliana GN=At2g44790 PE=1 SV=1 |
| Hmo-miR396b | comp14935_c0 | 3.5 | - |
| Hmo-miR396b | comp24998_c0 | 4 | sp\|O04714\|GCR1_ARATH G-protein coupled receptor 1 OS=Arabidopsis thaliana GN=GCR1 PE=1 SV=1 |
| Hmo-miR159a | comp20060_c1 | 2 | - |
| Hmo-miR159a | comp215345_c0 | 4 | - |
| Hmo-miR159a | comp24069_c0 | 4 | - |
| Hmo-miR159a | comp26007_c0 | 4 | sp\|Q9FNY2\|DPB_ARATH Transcription factor-like protein DPB OS=Arabidopsis thaliana GN=DPB PE=1 SV=1 |
| Hmo-miR159a | comp26997_c0 | 3 | sp\|Q756J5\|ESF1_ASHGO Pre-rRNA-processing protein ESF1 OS=Ashbya gossypii (strain ATCC 10895 / CBS 109.51 / FGSC 9923 / NRRL Y-1056) GN=ESF1 PE=3 SV=2 |
| Hmo-miR6020 | comp20177_c0 | 4 | - |
| Hmo-miR6020 | comp234190_c0 | 4 | sp\|Q42716\|C71A8_MENPI Cytochrome P450 71A8 OS=Mentha piperita GN=CYP71A8 PE=3 SV=1 |
| Hmo-miR6020 | comp23934_c0 | 4 | sp\|Q9M092\|WAKLM_ARATH Wall-associated receptor kinase-like 17 OS=Arabidopsis thaliana GN=WAKL17 PE=2 SV=2 |
| Hmo-miR6020 | comp27053_c0 | 4 | - |
| Hmo-miR6020 | comp301_c0 | 3.5 | sp\|F4JP48\|MSH4_ARATH DNA mismatch repair protein MSH4 OS=Arabidopsis thaliana GN=MSH4 PE=2 SV=1 |
| Hmo-miR6020 | comp327_c1 | 4 | - |
| Hmo-miR5072 | comp10331_c0 | 4 | sp\|Q93YW0\|EXEC1_ARATH Protein EXECUTER 1, chloroplastic OS=Arabidopsis thaliana GN=EX1 PE=1 SV=1 |
| Hmo-miR5072 | comp16966_c0 | 4 | - |
| Hmo-miR5072 | comp19822_c0 | 3.5 | - |
| Hmo-miR5072 | comp19971_c0 | 4 | - |
| Hmo-miR5072 | comp20064_c0 | 4 | sp\|Q4FZQ0\|YF1BB_XENLA Protein YIF1B-B OS=Xenopus laevis GN=yif1b-b PE=2 SV=1 |
| Hmo-miR5072 | comp24704_c0 | 4 | - |
| Hmo-miR5072 | comp26539_c0 | 4 | sp\|O23016\|KCAB_ARATH Probable voltage-gated potassium channel subunit beta OS=Arabidopsis thaliana GN=KAB1 PE=1 SV=1 |
| Hmo-miR5072 | comp26934_c1 | 4 | sp\|Q7X996\|CIPK2_ORYSJ CBL-interacting protein kinase 2 OS=Oryza sativa subsp. japonica GN=CIPK2 PE=2 SV=1 |
| Hmo-miR5072 | comp26960_c0 | 4 | sp\|Q9NX74\|DUS2L_HUMAN tRNA-dihydrouridine(20) synthase [NAD(P)+]-like OS=Homo sapiens GN=DUS2L PE=1 SV=1 |
| Hmo-miR5072 | comp27281_c0 | 4 | sp\|Q9SUG3\|ITPK2_ARATH Inositol-tetrakisphosphate 1-kinase 2 OS=Arabidopsis thaliana GN=ITPK2 PE=2 SV=2 |
| Hmo-miR5072 | comp27562_c0 | 3 | - |
| Hmo-miR5072 | comp27700_c0 | 3.5 | - |
| Hmo-miR5072 | comp28551_c0 | 3 | sp\|Q9LY84\|GDL76_ARATH GDSL esterase/lipase At5g14450 OS=Arabidopsis thaliana GN=At5g14450 PE=2 SV=1 |
| Hmo-miR5072 | comp28725_c0 | 4 | sp\|Q9SY66\|FRS11_ARATH Protein FAR1-RELATED SEQUENCE 11 OS=Arabidopsis thaliana GN=FRS11 PE=2 SV=1 |
| Hmo-miR5072 | comp28922_c0 | 3.5 | sp\|Q8LPT9\|GWD1_CITRE Alpha-glucan water dikinase, chloroplastic OS=Citrus reticulata GN=R1 PE=2 SV=1 |
| Hmo-miR5072 | comp29033_c0 | 4 | sp\|Q9SEK3\|HXK1_SPIOL Hexokinase-1 OS=Spinacia oleracea GN=HXK1 PE=2 SV=1 |
| Hmo-miR5072 | comp29937_c0 | 4 | - |
| Hmo-miR5072 | comp30004_c2 | 4 | sp\|Q9SGP2\|HSL1_ARATH Receptor-like protein kinase HSL1 OS=Arabidopsis thaliana GN=HSL1 PE=2 SV=1 |
| Hmo-miR5072 | comp30098_c0 | 4 | sp\|A2XUW1\|CDKG2_ORYSI Cyclin-dependent kinase G-2 OS=Oryza sativa subsp. indica GN=CDKG-2 PE=2 SV=1 |
| Hmo-miR5072 | comp360744_c0 | 4 | - |
| Hmo-miR5072 | comp8163_c0 | 4 | sp\|Q9FNF2\|SSY1_ARATH Starch synthase 1, chloroplastic/amyloplastic OS=Arabidopsis thaliana GN=SS1 PE=2 SV=1 |
| Hmo-miR529b | comp10349_c0 | 4 | - |
| Hmo-miR529b | comp1126_c0 | 4 | - |
| Hmo-miR529b | comp12721_c0 | 4 | - |
| Hmo-miR529b | comp14800_c0 | 4 | - |
| Hmo-miR529b | comp15107_c0 | 2.5 | - |
| Hmo-miR529b | comp16572_c0 | 4 | sp\|Q9LK03\|PERK2_ARATH Proline-rich receptor-like protein kinase PERK2 OS=Arabidopsis thaliana GN=PERK2 PE=2 SV=3 |
| Hmo-miR529b | comp17537_c0 | 3.5 | - |
| Hmo-miR529b | comp18445_c0 | 4 | sp\|Q5F3P8\|SET1B_CHICK Histone-lysine N-methyltransferase SETD1B OS=Gallus gallus GN=SETD1B PE=2 SV=1 |
| Hmo-miR529b | comp18700_c0 | 4 | - |
| Hmo-miR529b | comp18819_c0 | 4 | sp\|Q8BWM0\|PGES2_MOUSE Prostaglandin E synthase 2 OS=Mus musculus GN=Ptges2 PE=1 SV=3 |
| Hmo-miR529b | comp19830_c0 | 4 | - |
| Hmo-miR529b | comp25298_c0 | 4 | - |
| Hmo-miR529b | comp253931_c0 | 4 | - |
| Hmo-miR529b | comp25631_c0 | 3.5 | sp\|Q94JW8\|SPL6_ARATH Squamosa promoter-binding-like protein 6 OS=Arabidopsis thaliana GN=SPL6 PE=2 SV=2 |
| Hmo-miR529b | comp25957_c0 | 4 | sp\|Q8CFQ3\|AQR_MOUSE Intron-binding protein aquarius OS=Mus musculus GN=Aqr PE=2 SV=2 |
| Hmo-miR529b | comp26207_c0 | 3.5 | sp\|Q39199\|RECAC_ARATH DNA repair protein recA homolog 1, chloroplastic OS=Arabidopsis thaliana GN=RECA PE=2 SV=1 |
| Hmo-miR529b | comp27237_c0 | 3.5 | sp\|Q8LDB8\|RING2_ARATH E3 ubiquitin-protein ligase At1g63170 OS=Arabidopsis thaliana GN=At1g63170 PE=2 SV=2 |
| Hmo-miR529b | comp27339_c0 | 3 | sp\|Q9CA86\|PEX2_ARATH Peroxisome biogenesis protein 2 OS=Arabidopsis thaliana GN=PEX2 PE=1 SV=1 |
| Hmo-miR529b | comp30194_c0 | 3 | - |
| Hmo-miR529b | comp30297_c0 | 4 | sp\|Q9XHM1\|EIF3C_MEDTR Eukaryotic translation initiation factor 3 subunit C OS=Medicago truncatula GN=TIF3C1 PE=2 SV=1 |
| Hmo-miR529b | comp379904_c0 | 1.5 | - |
| Hmo-miR529b | comp440504_c0 | 4 | - |
| Hmo-miR529b | comp4643_c0 | 4 | - |
| Hmo-miR399a | comp15713_c0 | 3.5 | - |
| Hmo-miR399a | comp26716_c0 | 3.5 | sp\|Q8VY10\|UBC24_ARATH Probable ubiquitin-conjugating enzyme E2 24 OS=Arabidopsis thaliana GN=UBC24 PE=2 SV=1 |
| Hmo-miR535 | comp23942_c0 | 3 | - |
| Hmo-miR164a | comp12454_c1 | 4 | sp\|Q9FLJ2\|NC100_ARATH NAC domain-containing protein 100 OS=Arabidopsis thaliana GN=NAC100 PE=2 SV=1 |
| Hmo-miR164a | comp24001_c0 | 4 | sp\|Q9FLJ2\|NC100_ARATH NAC domain-containing protein 100 OS=Arabidopsis thaliana GN=NAC100 PE=2 SV=1 |
| Hmo-miR164a | comp27336_c0 | 3 | sp\|Q9FLJ2\|NC100_ARATH NAC domain-containing protein 100 OS=Arabidopsis thaliana GN=NAC100 PE=2 SV=1 |
| Hmo-miR164a | comp27657_c0 | 4 | sp\|Q8N5D0\|WDTC1_HUMAN WD and tetratricopeptide repeats protein 1 OS=Homo sapiens GN=WDTC1 PE=1 SV=2 |
| Hmo-miR164a | comp527128_c0 | 4 | - |
| Hmo-miR164b | comp27780_c0 | 2 | sp\|O23627\|SYGM1_ARATH Glycine--tRNA ligase 1, mitochondrial OS=Arabidopsis thaliana GN=GLYRS-1 PE=2 SV=1 |
| Hmo-miR164b | comp28899_c0 | 4 | sp\|P24859\|SEC14_KLULA SEC14 cytosolic factor OS=Kluyveromyces lactis (strain ATCC 8585 / CBS 2359 / DSM 70799 / NBRC 1267 / NRRL Y-1140 / WM37) GN=SEC14 PE=3 SV=2 |
| Hmo-miR171c | comp15551_c0 | 4 | sp\|Q6NQK2\|NAC8_ARATH NAC domain-containing protein 8 OS=Arabidopsis thaliana GN=NAC008 PE=2 SV=1 |
| Hmo-miR171c | comp22020_c0 | 1.5 | sp\|O23210\|SCL15_ARATH Scarecrow-like protein 15 OS=Arabidopsis thaliana GN=SCL15 PE=2 SV=3 |
| Hmo-miR171c | comp27398_c0 | 2 | sp\|O81316\|SCL6_ARATH Scarecrow-like protein 6 OS=Arabidopsis thaliana GN=SCL6 PE=1 SV=1 |
| Hmo-miR171c | comp28828_c0 | 3 | sp\|O81316\|SCL6_ARATH Scarecrow-like protein 6 OS=Arabidopsis thaliana GN=SCL6 PE=1 SV=1 |
| Hmo-miR171c | comp29850_c0 | 3.5 | sp\|Q42371\|ERECT_ARATH LRR receptor-like serine/threonine-protein kinase ERECTA OS=Arabidopsis thaliana GN=ERECTA PE=1 SV=1 |
| Hmo-miR171c | comp9827_c1 | 2 | - |
| Hmo-miR156 | comp379904_c0 | 3 | - |
| Hmo-miR156 | comp16977_c0 | 3 | - |
| Hmo-miR156 | comp15107_c0 | 3.5 | - |
| Hmo-miR156 | comp25631_c0 | 3.5 | sp\|Q94JW8\|SPL6_ARATH Squamosa promoter-binding-like protein 6 OS=Arabidopsis thaliana GN=SPL6 PE=2 SV=2 |
| Hmo-miR156 | comp28116_c0 | 3.5 | sp\|Q9ZVM9\|Y1461_ARATH Probable serine/threonine-protein kinase At1g54610 OS=Arabidopsis thaliana GN=At1g54610 PE=1 SV=1 |
| Hmo-miR156 | comp524107_c0 | 4 | - |
| Hmo-miR156 | comp30027_-c0 | 4 | sp\|Q4PE39\|SEC23_USTMA Protein transport protein SEC23 OS=Ustilago maydis (strain 521 / FGSC 9021) GN=SEC23 PE=3 SV=1 |
| Hmo-miR156 | comp1672_c0 | 4 | - |
| Hmo-miR156 | comp25650_c0 | 4 | sp\|Q6YZE8\|SPL16_ORYSJ Squamosa promoter-binding-like protein 16 OS=Oryza sativa subsp. japonica GN=SPL16 PE=2 SV=1 |
| Hmo-miR160b | comp18059_c0 | 1 | sp\|Q653H7\|ARFR_ORYSJ Auxin response factor 18 OS=Oryza sativa subsp. japonica GN=ARF18 PE=2 SV=1 |
| Hmo-miR160b | comp23023_c0 | 1 | sp\|Q84WU6\|ARFQ_ARATH Auxin response factor 17 OS=Arabidopsis thaliana GN=ARF17 PE=2 SV=1 |
| Hmo-miR160b | comp6967_c0 | 2 | sp\|Q653H7\|ARFR_ORYSJ Auxin response factor 18 OS=Oryza sativa subsp. japonica GN=ARF18 PE=2 SV=1 |
| Hmo-miR398b | comp24958_c0 | 4 | - |
| Hmo-miR398b | comp26053_c0 | 4 | sp\|Q9SKK0\|EBF1_ARATH EIN3-binding F-box protein 1 OS=Arabidopsis thaliana GN=EBF1 PE=1 SV=1 |
| Hmo-miR398b | comp27297_c0 | 3.5 | - |
| Hmo-miR398b | comp28501_c0 | 4 | sp\|Q944Q0\|WNK8_ARATH Serine/threonine-protein kinase WNK8 OS=Arabidopsis thaliana GN=WNK8 PE=1 SV=1 |
| Hmo-miR398b | comp29201_c1 | 4 | sp\|C0LGU7\|Y5458_ARATH Probable LRR receptor-like serine/threonine-protein kinase At5g45840 OS=Arabidopsis thaliana GN=At5g45840 PE=2 SV=1 |
| Hmo-miR398b | comp31195_c0 | 4 | sp\|P29675\|SF3_HELAN Pollen-specific protein SF3 OS=Helianthus annuus GN=SF3 PE=2 SV=1 |
