# Supplementary material for: Integrated sRNAome and RNA-Seq analysis reveals miRNA effects on betalain biosynthesis in pitaya
Source: BMC Plant Biol. 2020 Sep 22;20:437. doi: 10.1186/s12870-020-02622-x (PMC7510087; doi:10.1186/s12870-020-02622-x)
Supplement: Supplementary file 17 — Additional file 17: Table S10. cDNA sequences of 17 target genes. [file 12870_2020_2622_MOESM17_ESM.docx]

**TABLE S9 cDNA sequences of 17 target genes**

*comp24967_c0*

AATTCAATGAACCTACGCATGTGTATGCATGTATGCCCAACCAACATGTGCTGATTAACCATATCATACCTAGCTCATAATAATATACCCCACTGCATTGCAGCTCTCTCTCTCTCTTTCTCTGTCAGACGCGCACATACACAGGTGCCATCCCTGACATTCTGCCTCATCGAGCTAAGCTGACGTAGCAAACACTCCCTAACAAGCTCATCTTTCTCGATCAGAATGGCAGCAGAGAAGAGTAAGAAGCTCGAAAGCCGTCCGAGTGTGAAGAGAATAATGGTGAACAAAGGAGCATGGACTGCCGAGGAAGATAAGAGGCTGGCTGATTACATTCACTCACACGGCCCCAAGAAGTGGAAAACTGTCGCCCTCCGGGCTGGATTGAATCGATGTGGAAAGAGTTGCCGACTAAGATGGTTGAACTACTTAAGACCAAACATCAAAAGAGGCAACATTTCTCAAGAAGAAGAGGCCTTGATACTTAGACTTCACAAGCTTCTTGGCAACAGGTGGTCACTGATTGCAGGGAGACTCCCAGGAAGAACGGACAACGAGATAAAGAACTACTGGAATTCTCATTTGAGCAAGAAGGTTAATCGCCAAATTACGCCCAAATACATGGAATTCCCAGGCCCCCCTGTCCCTGTCCCTATCCCTGTTGCTCCCGCTCCCCCACAAAATGAGATTCGGGGCCATGATGATTTACCAGCCACAGGGGATATTAATGGAGCCTCTGGCTCTGACATTACCTTTGATGTCACTGACTTGTTTGATTTCTCCTCCGAACCCGCCTTTGGCTTCGATTGGGTCAATAAATTTCTCCAACTTGATGATGACCCTAGTAATTAATCTCCCAACTCACATTTGGTTAACTAATGACGATAGAAAAACAAAACATGAGAAAAATACGCTAGAGCACTGAATTTCAGTCTCTCATTTCTGCATGTGCCTGCTCTCGATCAAGAATCAAGATTCCTCTACTTGTGTCTAATGGATGACAATTGGGACCACGGTATTCAAGAAACTTGTACAATCAAGACCTGCGTCAATAAATGTCAAGCCGTCTCATCAAAAACTTTACGTGAAACTGTCTCCGATAGTGAAATTCACATATGATTAATTGGCATTATCGTGTCTTTTTTTTGGAGGGTATAAATAACAATTTGTTCCATTCCATCATTAATTCTTATTTGTTTTTTCTTTTGTATGTTGGTCCATGAGGAGGGCTTTAATCCATTGTATCTTAAGCCCAATGTGTATAAGTATTAGAAAAATCTTATAAAAGCAATATAATCATGTAGATCCCATAAAGTTGGGTGTATATGCCTTAGATATATACTTGTATAGGAGAAGTTAGTTTACCCGAACAAGTAGATATGCTATTCTCAAGAGACTGATGAAAGATATATTTGTATGATAATGAATTTTATGATACTGCTAAAGACTAATTCATTTTGATTATCTGGATTTATCCGAGTTGGGAAAAAGACCAGGTACTGAAAA

*comp234190_c0*

AAATGATGTGGTATGTAGAGCAGCCTTTGGAAGGAAATACAGCAGAGAATGGCGTGGAAGTTTCGTGGTGCTCTTGAAGCAATTTGTAGAATTGTTGGGTGAATTCCCTGTGGGAGACTTTGTGCCATGGCTGCACTGGATAGATAAAGTGAATGGATTGGAAGACAAGATGTGCAAAGTTGCAAAAGAGTTTGACGCGATCCTTGAACAGATACTTGAAGAGCATTTAGATAGTCCAAATACCCAAGGTAATGGGGATAAGTCTGACAACGGAGAAAAGATGAAAGACTTTGTGGATGTCTTGCTAGACGTTCAGAGAGATGAAACAGTTGGCTTTTCTGTAGACAGAGAATCCATGAAAGCCCTTATATTGGACATGTTTGCTGCTGGAACTGATACAACTTCCACATTGCTAGAATGGGCAATGTCAGAGCTCCTAAGGCACCCGAGAGCATTGAAAAAACTACAAGAAGAGGTGAGAAGGGTTACCGCAGGGAAAGCAACTGCAAACGAGCATGACTTGGAGAAAATGGAGTACCTGAAAGCAGTGATCAAGGAAACTCTAAGGCTGCATCCTCCACTTCCGTTACTAGTTTCTCGAGAATCAATAGAGGATGTCAAAATAAGTGGCTGTGACATTGCAGCAAGAACACAAGTTATCATCAATGCTTGGGCAATCCAAAGAGATCCCCTCTTCTGGGAGAAGCCCAACGAGTTCCATCCTGAGAGGTTTCTGGTGTCCACAGTAGACTTCAAAGGGCACAACTTCCAGCTAATCCCGTTCGGGGTAGGCAGGAGGGGTTGCCCAGGGATTCTTTTTGCCATCAGTGGTGTCGAGCTCGTATTG

*comp29967_c0*

GAAGATTAGATGAACCGTCCAATTTAGAAAGCTGTAGATACCAAGCAAGCATCCACATATACACAGTCATGGTGCTGGTGCTCCTCCTGGTTGTAGTTTGCTCTTTCATCTTCTTCCTTGTAATTCTCCCACATCGCGAAAAACATGGAAAATTCAATCTCCCTCCTGGACCCATAGGCCTCCCCCTCATCGGAAACCTCCACCAGTTCGATTCCTCGGCCCCTCACCTCTACTTCTCCAAGCTTGCCAAGATCTACGGCCCGATCTTGTCCTTGCGATTCGGGTGTAGATCAGCGGTGGTGATTCAATCAGCTGCTCTGGCGAAGGAGGTCCTGAAAACACAGGATCACAACTTCTGTACGAGGCCAACTCTGGTGGCTCAGCAGAGACTGTCTTACAATGGTTCAGACCTTGTTTTTGCGCCCTACAATGACTGTTTCAGAGAGCTTAGGAAATTAAGTGTTGTTCATTTGTTTAGCTCCAAGAAGGTTCAGTCTTCCGCTCCCATTCGACAAGAAGAAGTTTCTAGAATGATCCAGAAGATCACTTCTCTGTATTCTGCTTCGAAAGTCGTTAACTTGAGCAACTTGCTTATGGCTCTTACCAGCTCCATTATTTGCAGGGTTGCTTTTGGCAAGAGATATGAGGATGAAGGTGTTGAGAGAAGCAGATTTCATGAGCTCCTGAATGAAGTTCAAGCTATGGCGGCAGCTCCTTTCTTCACAGATTATTTCCCTTTCATAGGAAGCTGGCTTGATAAGCTAACTGGACTGTCATCCAGGCTTGAGAAGGTGTTTGAGAATATGGATGAATTTTATGATGAAATCATTAATGATCATCTTGATCCCAATAGGCCTAAAGATGATAGTCGTGAGGATATCATTGATGTTCTTCTGCAGCTCCGGAAGGAACGTTGCTTCTCTTTTGAGCTCACCTTGGACCACATCAAAGCGCTATTACTGGATATCATTGTAGCAGGAACTGATACAAGTGCAGCAATTGTAGTTTGGGCAATGACGGAGTTAATGAAAAACCCAACTCTGATGAGGAAAGTTCAAGAAGAGCTTAGAACCTTGATTCAAGACAAGACATTTATCGACGAGAGTGATCTCTCAAGGTTAACTTACCTCAAAGCTATAGTGAAGGAAACACTTCGACTGCACAATGCAGGTCCATTATTGGTTGTTCGGGAAGTCATCCAAAAATGTAGTGTACAAGGGTATGATATTCTCCCAAAAAGCTTGGTATTTGTTAGCATATGGGAGATCGGTAGAGACCCCAACTTTTGGACTGATCCTGAAATATTCATGCCTGAAAGATTTCTCAGGAGCTCAATTGATTTCAGAGGGCAAGATTTTGAATTGATACCTTTCGGAGCAGGTAGAAGAATATGTCCTGGACTGCTTCTCGGTTTAGCTAATGTGGAGCTTGCACTTGCCAATTTATTATATACTTTCGACTGGAAATTGCCAGTTGGAATGACGGAGGAAGATATTGACTTTGATGTATTGCCTGGAATCACAATGCACAAAAAGAATCCACTACGCCTCGTAGCAAAGAAGTTCTCTCATGTAAACTAAAAGAATCCACTATGCCTTGTAGCAAAGAAGTTTCCTCATGTAAACTTATAATGTTATGTTCAAACCCCTACCATTTGGTATCTAGTGTAATGGCTTATCAGAGTTCTCGACGTGCCTGAAAAATAGTCTGCTTGATTTATTCAAA

*comp24676_c0*

TCAAGTCGCCGCCGGAGAAGCAGTTGACATCCGGGTCCTCGATTTTCCCGCCGCCGCCGTCGGACTTCCCGCCGGCGTCGAGAATTACGGCGCCGTAACATCCATTGATATGCGATCCAAGCTTTTCATTGCCATTAATATGCTTCAAAACCCTACCCAAGATTTGATCCGACAAATTTCGCCCGATTGCATCATTTCGGATATGTTTTACCCGTGGACCTCCGATTTCGCGGCTCAAATTGGAGTACCCAGATTCGTGTTCCAGAGCTCCAGCATATTTTCTCTCTGTTGCGCTCACAGTGTCAGGGCCCACGCTCCTCACCAGCGCGTGGAGTCGGATGCCGACGTGGTTAGCCTTCCTGATCTTCCCCATGAGATATTGATGCTTAAATCCCAACTTCCCGAATGGGTACGCCACCCAAACGCATACGCGTATATGATAGATGTGATTGAGAAGGCAGCCCGAAAAAGCCAGGGATACGTGGCCAACACGCTCCGGGAGATGGAAGTCGATTACGTAGAGCATTTTGAATCGGTTACGGGCCTCAAAGTATGGCCCATGGGCCCAGTCTCTCGCTGGGCTGGTTGTGACGATGATAGTCGAATCAAAGCCCAACGGGGCAGGAACAATGGGCTGGACTCTTGGGCTTCCTGTCTAGAATGGTTGGACCGAAGGAAGCCCAGTTCGGTCCTTTATGTGAGCTTCGGGAGCTTGACCCAGTTCCCTGCCCAGCAGCTCAAGGAGATTGCGGCCGGGCTTGAATCGTCCGGACACAATTTTCTTTGGGTCGTACGAGGAAATAACAATAACAACAACGAAATTGGTATGAGAGACGATGACTGGCTCCCGGAGGGGTTTAGGGACAGGATGAGAGAGACAAATCAAGGGTACATAGTAGAGGATTGGGCCCCACAACTTTTGATCTTAGAGCACAAATCCGTGGGGGGAATGGTGACACACTGTGGATGGAACTCGATACTCGAAGGTGTGAATGCGGGGTTGCCTTTGGTTACATGGCCATTGTTCGCGGAGCAATTTTATAATGAAAGGCTAGTGGTGGATGTGCTGAAAATTGGGGTGGCGGTTGGCACCACCACATGGTCTGACATTGAAAAAGTGGGGCAGAAGGAGGTTGTCGGGAGAGAGAAAGTGGCGGTAGCCGCCACAGCAGTGTTGGGAGAGGAGGAACAAGCAACTAATA

*comp15143_c0*

GGCAATATCACCCCTGATGAGGAGGAACTCATCATAAGGCTTCATAAGCTTTTAGGCAACAGATGGTCTTTAATAGCTGGGAGGCTTCCAGGACGAACAGACAATGAAATCAAAAACTATTGGAATACCAACTTAAGCAAGAGAGTGAATCAACATTGTAAAACCAGTTCCTCCACCACTAAGCAACAACAGAAGAAGTCCCGAATTGATCAACAGAATAAGAAGCTGGTTGCCGACAGTGGACCCACCTTGACCCCAGCGTCCAGTGTGGTCCGAACCAAGGCGGTCCGGTGCACCAAAGTTTTCATTAATCCTCCAGATCCACCTCAACCAAGCACCGATAGCATGTATTTACAACCGAACTCAATTGGTCATGATAATGGCGAATGTTTGCTTGAACACATTTCGCTGGCTGGTGTGCCAGAGGAGGAGAACAAGATAGACATGGATTTCATGGGTAACAATCTTGACGTGGGAGATTTTAGCCTAATTTCAGAGATCCTTGATTCCGATAATTTTTCGAATATTTTTGATTTTAGCAATGAGACTTGTAACGGGGATGATACCAACAATAACAGTAGTAATGAGTTATCTCCCAATTCCCCAGAATATCATCCCTTTGTTTTCTCACAAGAGATGCTTGAAGATTGGCTTGGCGATCAACAACTCTGACTTTGAGTTTTGGACATACTACTGATTTGGTAACGATTGTAGCATCTTTTCTTCCTTTCCTTTTTCCTGTTCAAGGATTGTTTCCCCGCTCCCCCCCCCCCCCTCTTCTTTCCTTTTTCTTTTGTGAGGGTAGGGGAGTGCCCAGTCTACTGAAGTGGTCAGAAAACATTTTGCTGCATGTTGTATTGTTGAGCAGAATAATACAGATTATCTTCT

*comp24362_c0*

TACATACAGATACAGAGAGCAGTGTGGAGATGGGCAGGAGGCCATGTTGTGAAAAAGAGGGGTTGAAGCGGGGCGCATGGACCCCTATAGAGGACCAGACTCTGATTGATAACATCAAGGTCCATGGAGAGGGGAGGTGGAGGAATCTTCCCAAGAGGGCAGCTGATCATCCAAGAAACTAAAACAAGAAATAAGAAAAATATATTTGTATATAGTTCATATTTCTCGAATTTGATGAAGCTTCAATGAAATTCGATAGGAAATGGGTTCCCATTCGTGGTTATTTTTGATGTATGTATAGGTCTGAAGAGGTGTGGGAAGAGCTGTCGATTGTGTTGGCTCAACTACCTTAGGCCGGATATCAAGAGGGGCAACATTACTCCTGATGAGGAAGAGCTCATTATAAGACTCCATAGGCTCTTAGGCAACAGTGGTCTTTGATAGCGGGAAGACTTCCAGGCCGAACAGATAATGAAATTAAAAACTGTTGGAATACCAAGTTGGGCAAACGAGTGAACCAAGATTCTGAAGTTAACACAGCCACTACCACAAGTAAGAAGTCTCGAAAAAATAAGAAGAGCAAAAGGGAACTCTCCAATGATAAAACCGCAAAACCCATGTCTACACTACCCAGCGAGTTTAATGTTGTTCGAACCAAGCCAATCCGATGCACCAGAAGTTTCATCAATGAACCGCAACATGGTCTTGAAGGCGCAAATAATATCATGACAAATAACTTCAAGGAGTATTCGTCATCTTGTTTTAGTTCTTCTACACTAGATATAGCATCAGAATTTATGGGAAATATGGAAAACTATAGGATGATCTCAGAGCTCGTTACTCCAGAATTTTTAGGTGCTTGTGATTTTAGTTATGGATCTAGCGGTGGTGACAATGATTTATCCCCGAGTTCAGTAGAACATCCTTTTGTTTTGTCACAAGAGCTGCTTGAAGATTGGCTTGGAGAACAACCAGCAAATTTGTTTTAGTTTGTAACTTTAGAATTGTCAGCCTGTTGGATTCGGAAAGAATATGCAATGTTTAGCTTTTCAAGTAGAGAAGAAACTAGGCAACCTCAGATTTTCAATTTAGATTAGAAGCCAACATAATACTGCGTAGATCTCATATTAATATAGTCTTGTTTTGTGTTGGATTTGTAATAATGGTTTTTTGTCATCGATTCTGAATATCACTAACTACATACATTTTTGAATTAAAAAAAAAAAAAAAAA

*comp403340_c0*

TTGCAGAGATGTGGCAAGAGTTGTAGGTTGAGATGGATAAATTACTTAAGACCAGATCTTAAGAGGGGAATGTTCTCTCAAGAGGAGGAGAATCTCATCATTCATCTCCACCAAATTCTTGGAAATAGGTGGGCACAAATAGCAACACATTTTCCAGGAAGAACAGACAACGAGATCAAAAACTTTTGGAACTCATATATCAAGAAGAAGCTAATAAAGCAAGGGATTGACCCACAAACCC

*comp15849_c0*

AAGATTACCAGGAAGGACAGACAACGAAGTGAAGAACTATTGGAATTCTCACATACGAAAGAAGTTAATTCGCATGGGAATTGACCCCAACAACCACAAACTCCATCAACATCTTCCTTGCCTTAGAACCCCCCAACAAACACCGATTTCATTCATTGCGAATTCATCTGAATCCAAGAAAAACAATGCGATGTCTGGCAAGCAATTACCATCCTCA

*comp36993_c0*

ATGGCAAAAAAACCAAAAACTCATCTTACATCAATGGATTACTACACTGTATCGTTGGTAGTCCTCCCCATTATTATCTATGTCCTCCATAACTTCCTCAAAAAGTCACCTAAAACCAAGCTTCCTCCAGGACCAACACCATGGCCAATCATAGGCAACATCCACCTCCTTGGCTCCAAGCCTCACCGCTCTGTCGCAGAGCTCTCCAAGATTTATGGACCCTTAATGAGCCTCAGGCTCGGGAGCATCACCACAATAGTGATATCATCCCCCGATGTAGCCAAAGAAATGTTCCTAAAACATGACTTAGCCTTTTCTTCTAGACAAATCCCAGATGCTTCCCGGATAGCAGGCCATGACAAGCTCTCCATTGGGTGGCTTCCCGTATGCCCCAAATGGCGGGATCTCAGGAAAGTCTTGGCCATCCAGTTGTTCACCAACCAACAGCTCCATGCGAGTCAGGGCCTCAGGAAGAAGAAAGTGGATGACCTTGTTCAGTTTGTAAAGGGGCGCTCTGAAAAGGGTCTAGCCATTGATATTGGCAAGGCCGTGGCAACCACCTCACTCAACTTATTATCAAATACATTTTTTTCAATGGACTTTTCTAGCTATGATTCTTCAGTTTCAGAGGAGTTTAAGGATCTTGCTTGGCATCTGTTGGAAGAGGCTGCGAGGCCAAATGTGTCGGATTTTTTCCCATTGCTTAGGCCTTTGGATTTGCAAGGGGTGAGAAGAAGGCAGACTATGTACTTCCACAAAATGATGGGATTTTTTGAGAGAATTGTTGATGAAAGATTGAGGGATCAAACAGGTGCAAATGAAGATTTTTTAGGCACTCTACTCAAGCTTGTCAAGGAAGATGAGCTCACCCTTGACGATATCAAACATCTGCTGATTGACTTGTTTATAGCGGGAACAGATACAACTTCCAATGTATTGCAATGGGCAATGACTGAATTACTACGCAATCCAGAAAAAATGACAAAAGCACAAGCTGAAATCGATCAAGTCCTAGGCGAAGGTCAATCAAGGTCAATTGAAGAATCTGACATCTCAAATTTGCCTTACATACAAGCAATACTGAAGGAAACATTGAGGTTGCACCCACCAGTCCCCTTTTTGGTACCCCATAAGGCTGATTGTGAGGTACAATTATGTGGCTACTATGTGCCAAAAAAATGCACAAGTGTGGGTGAATCTATGGTGCCTCAGCCATGA

*comp35191_c0*

ATGGATTACTACACAGCTTTGGCAGCCATTCTTCTCTTCTTATGGACTTGTTTCTACTTCATGAAACTTAACCCATTTTCCTCAAAAGCAACCTCAACCATTAACATCCCACCTGGTCCTCGACCTCTGCCCATCATCGGCAACCTCCACCAGCTCGGTGAGAGGCCTTACCGCACCTTGGCTGACCTAGCCAGGGCTTATGGCCCCATAATATCGATCAAGTTCGGAAGCATCACCACCATAGTGGTTTCATCTTCTGATGTAGCCAAAGAAATGTTCCAAAAACACGACCTTGCCCTGGCCAACAGGAAGGTCCCTGCAGCGGTCAAGGCAAATGGGCACGACAATTTCTCGATTGCCTGGCTGCCTGTGACCCCCAAATGGCGGTTTCTCAGGAAGATCTCTGCCATCCAACTGTTCTCCACTCAACGACTCGATGCTAGGCAGTCACTCCGCCAGGCTAAGGTAGTTGAGCTCCTTGACTACATCAAGGCGCGCTCTCATGCAGGAGAACCAGTTGACATTGGAGAGGCCGCCTTCACAACTTCACTTAACTTGTTATCGAACACATTTTTTTCAATGGATTTGGCTAGTTATAGTTCAGCCGCATCAGGGGAGTTTAAAGAGCTTGTTTGGAAGATAATGGTGGAGATTGGGAAGCCGAATTTGGCGGATTGTTTTCCTCTTGTAGGGTTCATAAGCAAGATGAGTGTGAACAGGGATTTGATGGGGTATGGAAATAAGTTGAATGAGGTGTTTGCAGAGATCATAGAGAAAAGACTAAGTGCAGCTAATTCATCTGAAGGTCGTGGCAATGGTGATGTTCTTGATACCCTACTTAGAATCATGGAAGAAGATGACTCTGAGCTCAGTCTTGATGATATCATGCATCTTCTCATGGACTTCTTTACAGCCGGGACAGACACAACCTCGAGCACCTTAGAATGGGCAATGACGGAGCTACTACATAATCCGGAAAAAATGGCAAAAGCTCAAGCTGAGCTCGAACAGGTCCTTGGCAAGGACACTGTGTCGATTCAAGAATCTGACATCTCAAAATTGCCTTACTTGCAAGCTACAGTGAAAGAGACCCTAAGGATGCACCCACCTACTGTATTTCTGCTGCCTCGAAAGGCAGATTCCGATGTAGAATTGTATGGTCATTTGGTACCTAAGAATGCCCAAGTCTTTGTCAATCTTTGGGCAATTAGCTACGACCCGAGTACTTGGGAGAACCCGGATTCGTTCTCACCCGAGAGGTTCTTGGATCAAGATATCGATATGAAAGGACAAGATTTCGGGTTCATACCATTTGGAGCAGGAAGGAGAATATGTCCTGGACTGACATTGGCATATAGGATGTTGAACCTTATGTTGGGAACACTAATTCATGTTTTCAATTGGAAGCTTGGCGATGGGTTGAGTCCTGAAGATTTGGACATGACTGACAAATTTGGGATTACCATACAGAAGGCTAAGCCCCTCCGGGCTATTCCAATTCTAAAGTGA

*comp28219_c0*

TGGGTTTCGACCTACCGTGAGTTTCGGTCTTCCATTCCCAGTTAGCTATAGTCGATTTGAGGGCCTCGTATGAGTTTGACTGTACAAATGCAGTGATGAGTTTGATCGTACAAATGCAGTGATGAGGGTTAAAGCGATTCAACAGGCTGTTTGATTCAACTGGGGGTTGGGGCTTGATGAATTGCAATAAGGGGAAGGGAACAACGAAAGGAAGTTGAATTATGTCTGGTTGGAAGTAGAAATACAGCTGTGGACTTCGGTTTGTTAATGTTGGGGTGAAGATGCCTAACTAAGTGACTCATCGGCTGACTACTGTCTGATTTTTTACTACACCAGCATTATTGATGTAGGACACCCTAAGAGCAATTCCCACACCTAGACAAATCACACAAGAACTTTCAAAACTCAATAATTTAATTAATTCAAGTGTGTCTTTCCCCAAAGTACGAGGGGGGTTTATATAGGAATCAATACAACTGAAAAAAGGAATAGGAAATATATCCGAAGATTACAATAAGATTATGATACCTAAACATAATCTAAGAGATAAAAAAAAGATAAGATTTTATCCTAAAAGATAACCTATGAAGGTAATAAATTATAAAAATACTTCACATATGCCAGTATTAAGATATATCCTTATCTTCTTAATTCTCCTTTATCCTTGTCCTGAATCATTTTCCCCTGGTTGGAAAAAACTCACCCTCGAGTTTTATACAGACGTAGAAAAATGGAATCCAAGCAAAGAAACAACTTCTCGACTATGTGCAAATGTAAAAAATGTAGGGCTTTCAACTTCTCTAAGAAAAGAGGCAGTGCAATAAGCGGATGCAGCTTCTTGTCCGCCTTAGTTTGAAAAAGCGCGAGGCGCACTGAGGCACAAAGGGTCCCGAGGCGCAAGGCGAAGGCACGCGCTTCAAGGACGCGAGCCTTTTTTTAGCTAAAAAAAGGTTCGCGTCCTTGAGGCGTGCGCCTTCGCTTCGCGCCTCGGGACCCTTTGCGCCTCAGTGCGCCTCGCTCTTTTTCAAACTAAGTTGGTGATTTAGGAGGCCATATATAAATTGATGGACTCAAGTGCAAGATGGATTTTGCTCCTTCTGCTTATCGCATCAGTTTCTCTTCGGGCAAATGCCTCTTCTGTAGTTAATGACTTCCAGCACTGTGAAAATGTTGTAACTAAGTGGGCCACATCTCAACTTAATCAAGAAAGCAAAGAAGATAAGCATACGCTGCGTGATCTGTTGTTTTTCCTCCATATACCAAGGACTGGAGGGCGCACATATTTTCACTGTTTCTTGAAAAAGCTGTACTTGTCTCAGTTTGAGTGTCCCCGTTCTTATGATAAATTGCGATTTAACCCAAGAAAAGAAAATTGCAGGCTCTTAGTCACCCATGATGATTATAGCATGATGACAAAGCTGCCAGAGGAAAAAACTTCTGTGGTGACGATACTAAGAGATCCTGTTCATCGTGTATTTAGTGCATATGAATTTTCTATTGAAGTAGCTGCCAGGTTTTTGGTGCATCCTAATTTAACTTCTGCCACACAATTGGCTGGACGATTGCGAAAGAAAACCAATGGAGTTAGCACATTAGATATTTGGCCGTGGAAGTATTTGGTTCCATGGATGAGAGAAGACCTATTTGCTAGGAGAGATGCTAGGCTACACAAGAATCAAAATCATGTAAAGAGTGATAACTCGTACAATATGGAAAATATGGCTATGCCGCTGCATCAGTATATTAATGATCCAATGGCTTTGGATATTGTACACAATGGGGCTACTTTTCAGAATCACATGAAGTCCGTGGTTGTGTTCAGAAGCATCATTCTCTGGGTAAACTTGTCCTTGATGTGGCTAAGAGGCGGTTGGATCACATGTTGTATGTTGGATTGACTGAAGATCACAAAGGATCTGCAACAACATTTGCACACGTGGTTGGTGCACAGGTTATCTCCCAATTGTTGCCATCAACTTCTAGCACAGAAAGGCTGGCTGACAATACATCAGGAGAGCTCCCCAATTAAAGAGTTTAAATCTGCAAATGATAGTTACAAGAGTGGCCCTAGCGATGAAAATACTAGTGATACTCTTCCATCTGAAGTGGTTGAAGGATCTAGCCAAAGTACAATGACCGTCGGGAAACTCATGGAGGCGTATGAAGTCTGCATTACTAGTTTACGGAAGTCCCAGTCACGCCGACGAATCAACTCTCTGACAAGAATAGCCCCTGTGAACTTTACAAAGGAGGCACGTCTTCGGGTACCTGAATCAACTGTTGAGCGGATAAAATCACTCAATAGACTTGATCTGGAGCTTTACGAGTATGCTCAAGACATCTTTGCCAGGCAACATGATCGAACAATGCAGAAATTGGTGAGTGCCTATGCCATGTATCTTATATAGATTAGTATTTTATCATCCGATGACTAGATTGTGCACAGCTAATGAGTCTTATTACAATTATTTCGACTTTGAAGTCTACCAAAAGCTCACACTGCCCTTTTTTTTGCAAAGTAGGACTAGGGAACCCCCCCCCCCCCTTTTAGGGGAATAAGTAGTCAATGCCTGCTAAAATAAAAATGCCACCTCTGCAATAATAGTAGAGTTATACGAAGTAACTTTTGCACATGTACCCCTTGATACCCTAACCTTTGGTGCAGCTTCTGGGAAATGATTTGGTTTCATTCAGGTGAGAAACTCAGTTTTCAGAGAAACATTGCAGGAAACAAAAGGCACTGTTGTTCTCAATTCCGTTGGGGCTCCCCCTTGGAGAGAGAGTTTACTAGTTATGGCTGTCGTTTTGGTGGGCGTGTGTCTCTTTATAAATGCAAGGCGAAGAACATCTAAAGTTAAGGTGTGAATGTGGCTATGATTTTACACACTTAAGGGTGTAAGAGCTGTTCAATTTTAAGACACAGCTGCAACCGCTTTTTGCTGTTGGGCATCGTTACACAGTATCCAATTTTGAAGTGATGGCAACTGAAGATTCATTAGCCGCATTTTAGTAGTGTAAAATGATTCACTCGGTCATTCTACTGTACTATGGATAGTGAATTTACATAGCTACTTGGAACCATGATTGTTATGTTCAGATTTGAGTCTCATTGACAAATCTTACCGTTATATCTTTTCCGGTTTGACTGTTTCTCTCCACAATTTTGCTAGTAGGAGCTTTGGGTCTTATCATTTAAGGTTTCCTAAGTATCTCAGTTAAAGCTCTTCCTTCTGCCCCCCACCAGCGGGAGAAAATGAATGTTGAAGTGGATCTGTTTGTGTAAAGGC

*comp25631_c0*

CAGCTCATAATCAGTTGAATTGAAGAAATCTGTGGGGGGGAAACTGTTCTTTTTCTTTGTGTCAAATTCAATGTTGATGAAGGATACCAGAAAGCAAAAGGGCAAAAGCAAGGAGCTTGCTTGTTTGTTTGAGTGATTGCAATTTGGCATCAAAAGCTACAAAAAAGAATGCAGACACACTCAGACATCGAGATATTGTCCGAGATACTCATGTTGACCCTCACTGTATCTGCCTCGACCTCCTCAAACAATTAGCTTTGCTTTCTCAACTTCTCTGCAGAACTTTCTCTGTGGTTCCTTGTGAATGGATTCTTGGAAGCCCTTGTCAGATGGAAGGGGTGTTTGGTTATCTGATGAATTAGGTATTCCAGCTGATGGTCATGGAAGATGTAACAACCAAGAAATGGGGTATGAAATCAAACCCTTTTCCAATATTGAAGGTTGCCCTCTTGTCTCAAATACACAGGCACTTGAGAGATTGGATTTTATGGATTTGGGTTTCTCCGATGAGATCAGAAAGTCATATTCAGTTTCAAGGGAACCAAATGGGGAGGTTTTCAGTGGTGAGATTGCTACTGATTCTTATGGAAATGTAATATCTCCACTTTGCGAGGAGAAAGATTCGGGTTCGAGATTGTCAACTGATTTTATGCAATCAAACAGCCAGGATTACTCGCTCATTGATCTGAAGTTGGGTAGGCTGGCAGATGGTGGAGTTGCCGTAACTAGTGAACCTGAATCATCTGCTGTATCATCTTTTCTGGGAAAGAGAGCTCGATCCACCAAATTGTATCCACGGTCGACATGTTGTCAAGTGTTTGGTTGCAACAAGGATCTTAGCTCTTTCAAAGGGTACTACAAACGGCACAAGGTTTGTGATGTGCATACGAAGACCACCAAAGTTATTGTTGATGGAATTGAGCAAAGATTTTGCCAGCAGTGTAGCAGGTTCCACCTGTTGGCGGAATTTGATGATGGTAAGCGTAGTTGTCGTAGGCGTCTTGCTGCCCATAATAAGCGTCGAAGGAAGCCCCAATTGAATTCTCAGCCAGGTATGGGGATGGCCGGGTTTTCCTCGTCCTTCTCATTTCAAGAAGTACTGCCTAGCATTATTGTTGGCTCAGGACAAAGAATGGATCATTTTCCAGCAGGTGTGAAGGAAGATATTGTTGCCATAGGAAACCTTGGTGTACAAAGTTTATCTGACATCCATCAAAATGGTCGTGCTCTCTCTCTTCTGTCAGCTCCATCACATAGCATGTCGCTGGGAGCTGCAATCACTAGCCCCCTGATCCACAAAGGCAGCCATGCAGACTCTAGTCCAGGACATTTCGCTGGCCATTCAGGAGGCCTTTTCAGAAAAGTTTCTCAAAGTGGACTCCATGCGTCTGGCATGTACTCAATGGACGGTGACCATGTGGAGCTCACCATGATTGATCCACGCGATGATAGTGTGGTTGATCTTAAACTCCAAGGTGATGACATTTGCTGCACATCCAATATAAGGAATGCTAGTAATAACCAACCTTTAGGGCATGGCGTCATCCTTGATTTGCTCCAACTGTCATCACATCTTCAGAGAATCGAGCAACAGAGGCACTCAACCGAAAGTGAAGCAGGATGAAAATGACTTCTTCACGGTTTTCAGTGTTCTGGAGGGCATCAGTATCATTGTAGCTGCGTCACTGTGGGCATCATCATCCTCGTACTAGCACAAAATATTACAGGCAAAGGCTGCCATCTTAGAGTAAATTTTAGTTGAAGTTGAGATTCACGTACGACCTTTCAGCAGAACAAGGCTCTAGGTTTTGTTGGAAGCAGAAAGTTTGTTCTGCTTCAAACCCAACAACAACACCTAGCAGTGTAGAGTATGCTTTTGCGAGATTCAGGAGATCCAGATATAAACATTGCTATCCTTGCAAGTGCAACTGTACAAGTGCAAGTAGAGGGACCGGTTCCCAATGTTGCGTTCTGCTTCAAACTCGCTCCCCGAAAAAAACTGAAGGGGTGAGAAAAAGTCAAAGTCTGCAGTCTCCATGGTGGTTGGACTCCTTACTATCTTGAAAATCTAATGTTCTTTAGGCGTCGCTTGAGAATTGGAAGGGTTCAATATTTTGGGTCTACAGCGGTTGGTTGCATTCTGGCCTACTTCTGGAAGTAAATTACTGTCTTTTTCCACTTGT

*comp27657_c0*

TTTTCTTTTTCGCTGATCGTGTTAGGGTTTCTGCTCTTACGATTTCTGATCAACGATCGTTAATGGAGTCCTTTGCCTTTCATGACGGCAACATTACCAATTTGATTGATAGCCGTACTCTCGACTTTCGTCATGATGTCAAGCACAGTCTACAACTGCACTCATCTTTGGTGAAAAGGCTGTCGCTGGAAAGAGAATTGGAGGGGCATCAAGGTTGTGTCAATACTATTGCGTGGAACTCGAAAGGTTCTCTCCTTATATCTGGATCAGATGATACACGGATTAATATTTGGAGCTATTCTGATCGTAAACTGTTGAAAAGCATTGACACGGGCCATTCTACCAACATCTTTTGTACGAAATTTATCCCCGAGACTTCTGATGAACTAGTGGCATCTGGTGCTGGTGATGCAGAGGTTCGCGTGTTCAATATTTCTCGCTTAAGTGGGAGAAGGGTTGAAGAGACTTCCATCGCTCCATTAGCTCATTTTCAATGTCACACAAGGAGAGTGAAAAAGTTAGCGGTTGAAGTTGGGAACCCAAATGTGATTTGGAGTGCTAGTGAAGATGGAACATTGAGGCAGCATGATCTTCGTGAAGGTTGTTCTTGTCCTCCTGCTGGATCTTCCAACCAAGAATGTCGCAATGTTCTACTTGATTTACGTGGTGCTGCAAAACGATCCTTGGCTGAACCTCCTAAGCAGCCTCTTCATTTAAAGTCTTGTGATATCAGTGTGACAAGGCCCCATCTACTTCTTGTTGGAGGAAGTGATGCATTTGCTCGATTATATGATAGAAGGATGCTGCCACCCCTCTCTTCCTGTCAGAGAAAGACTTCGCCGCCTCCATGTGTGAACTATTTCTGCCCAATGCATCTTTCTGAACGTGGACGGTCAAGCTTACATCTGACGCACGTAACATTTAGTCCAAATGGTGAGGAAATTCTTATAAGTTACAGCGGAGAACACGTGTATTTAATGGATGTACATCAAGCTGGTGGAAGTTCTGTGCAGTATACATCTGGAGATGTTGTAAAGCTGTGGACCTATTCTCCGATTCTTAATGGTGTGGAATTGACTTCCTTGGGAGCTGCTGGCTCTGAGCAAGTTTCTGATACCAAAAGCTGTGCTAAGGCAGAGCTACAAAAGTGTAGAAAGCTGGTCAAAATTGCAGAAAAATGCTTGAATGAAGCAGCAGATTATTCCTATGGAATTGAAGCATGCAATGACGTTCTAGATGGAGGTTATAAAATTGATCCTATGCTTAGGCATGATTGTCTCTGTCTGCGTGCTGCCTTGCTTCTTCAGAGAAAGTGGAAAAATGATGCTTATATGGCTGTAAGGGACTTGTACCAAGCTCGGAAAATTGATTCATCTTCATTTAAAGCACTCTTCTATATGTCTGAAGCTTTATCGCAGTTGGGCAAGTACAAAGAAGCGTTAGACTTTGGTCTTGCAGCAAAATGCTTAGCTCCCTCGAGTGCTGAGGTGGCAGAGAAGGTGGATAGCATACGGAAGCAGCTGTCAGCAGCCGAAGCAGAAAAGAATAACAAATCAAATGATGGAGGATCTAGGGCCCAACCACAACCTGGCAGAGTTATATCATTAAGTGACATACTTTACAGGTCAGAAGCAAATAGTGATGCTTCACAAGATGGCCCTAGATCGGAAAGAGAAGACTCTGATTATGATGAAGAACTAGAGTTGGACTTTGAAACATCGATGTCGGGTGATGAGGGTCGTGATGTTGATCCCAATGTTCTTCATGGAAGCTTGAATTTGAGAATTCATAGGAGAGCAGATTCAGCTAGAGAAGCTGGTGCTTCAAATGGTTCATGTGGATCTCCATCTTCATCCAACCAAAATGGCAAACCAAGTTATCAGCCAGAAGCTGTCATTGACATGAAGCAGAGATATGTCGGGCACTGTAACGTAGGAACTGACATAAAGCAAGCCAGTTTTCTGGGTCAGAATGGTGATTATATTGCTAGTGGAAGTGATGATGGTAGATGGTTTATTTGGGAAAAGAAAACTGGTAGATTAATCAAAATGCTTCATGGTGACGAAGCTGTGGTAAATTGTATACAGTGCCATCCTTTTGATTCTCTTGTCGCTACTAGTGGAATAGACAATACAATTAAGATTTGGACCCCGAGCGCTCCAGGCCCGTCAATGGTAGCTAGAGGCGCTGCTGGTCCAGAAACATCGAACGTCTTGGAAGCTATGGAAAACAATCAACGTAGACTATCTCGGACACGTGAAGCTATTCTGCCGATTGAGCTGTTGGAGCGCTTTCGCATGCATGATTTTACAGAAGGAACATTGCATCCTTTTGAGTGTGCTCAGAGCTAGTTTGCTTCGTGCAATTAGGCTGAAAATGCAGGTAGTTGGGTTGTCATGATCAGTTCCATCATCATCTGATTCAATACAAGCTTGTCTGAGCATTCGATGAGACATAGTACAGTTAACCTGTGAGGCTTTTGAGCACCCTGATTGTACAACTACATGAAATAATCTCCATGTTCTATCTTGAGGTTTAAATCTTGCTGAGTCTCGCTGGATGAGTTGAGGGATATAGGTGGGATCTGGGACCCCGACACTCTGTGTCTATGTATATCATTCTGTAGGTTTGTGGAAAATAAGCTATGCTCCGAATAGTGTTGTTGCTTTTCCTAAATTCAACAACAAATACTTCAATTCTCATTGGTCCTGATTTTTCTGTACATAAATAAACTTTCTGTCTTAGCTGAATCTTGGCCTGTGTTCCCGAGGTATTATTCTATTTCAGGCAAATCCTTAAATGTAAATAAGATCTGACTTAAATCACGAGCATACTGTTGGTTTACCCT

*comp26829_c0*

ATGGATTTTTGCCAGTGATTTTTCCACTCCTCCCCCAGAAACCCCCCCTCAAATTCTTCCCCCTTTCAATCTCCTCTGGCTCTTTGAGTGGTTCTCCTGGAAGGGTTTCTGATGAAAAACACTTTAAACACAGTAACAAACTTTTTTTGCATGAGCCGTAGTCTAGATTGCACATGATGTGTAGGATAAGCCAAGGGGGTGTAGATTAAGATAATGCACTTCACATTGCTCACTCAAAAAGAAAGTTGGGAATTCAAAAAGCTACTCTGGTATTATATTCACAGCACCAACAAAGAAGGGCAAAGATCAAATTTTTGTACAATATTGTGGTTTGTTTATCAGGAATCTGAAAAGGGTCTAAACCCTCTGTCACTTGCTCTTTGTACTTTTTTTTGGGAATTCTCTCTCTCTAAAAGGTGGGGTTCATGGAATTGATTGATGTTCAGAGGATTGATGAGCTTGTTGCTGAGGAGCTTGCTCTGAGAGAGAGAGCCTAGAAGCTGGTTGAGTAAAGTTCAGAAGACCCTTGGTTTGCCACTCAATCGTACTTCTTTTTCATCTTCAATCTTAGTAAGAATTGCAATTGCAATTGCTTTTATTCTGCTTCAACTTTTCATATTTCCAACCCGCAATGTCGTCTGATATAAGCTAAATTATAAGCATGTCTGAGTTCCTGGTGTGAAGTGTGGTATGTTTTTGGTGAATTTGGTTGTACCCATATTAAAAATTTTGTGTTGATGGCACTTTGTTTGAGGAACGGTGATTATAGTGTGAACTGTGTTGTGGGTATCTTTGGTTTGGTTAATTTGTGTTATTGAGCTAGTTCAAGATCCTGCTTGAAACTAGGACTAGGGTCTGTTTTAGGGTTCCTTAGTGATAATGGGGACAAATTGCTTGCTGCTATCAACAGTTGGACCGCCAATAAAGCGGCGAAAAGGGTTGATAATTAAACAGGCAGGCAGAGGCTCATACAGAGGAAGTTAGGTTAGAGAATTGAGGACAAATTGGATTGCTGATTTTTAATTGTGTGGCAGATTGATATTTCAACTAACAAAACCGATTGCAGCACGGGGAAAAGTGTTTTTAGTTGGGGGAGGGGGGTGTTTGGATTTTGGATTTTGGATTTGTGGGTAAGTAAGAGGGAAAATGGCTACTCACCTACAACAACTGCTTCAGAGCCTTTGTTGGAATAGTGATTGGAATTACGCGGTCTTCTGGAAGCTCAAGCGTCATGCTCGAATGGTACTGACTTGGGAAGATGCTTACTATAACAACAACTGCAAACATGATGCTTCAGAGAGTAAAGGCTCTACTGAAACACTTGAATCCTTACATGATGGGCACTTAGCACATGATCCTCTTGGCCTGGCTGTGGCAAAGATGTCTTACCATGTATATTGTCTTGGTGAAGGGATTGTTGGACAGGTGGCTGTTTCAGGGAAGCATCAATGGATTTTTGCCAGTGATTATGCAATGGATCCCTGTCTTTCTTTGGAGATCTGTGATGGATGGCAAGCACAGTTCTCAGCCGGAGTTAAGACCATTGTTGTTGCAGCTGTTATTCCGCATGGAGTTGTACTGCTTGGCTCCCTGAATGAAGTCAAAGAAGATTTTAAGTTATTGATTCATGTCAGAGATTTATTTTCCTCACTTCAAGATTCTTTAGCATGTCATGTTCAACATCCAATAGAGGGCAGTGTGCAGAGTGTGTTGCAAATGTCAGATACATCTGCAGAGATTTTTGATCCAGATGTTCTTCAAGAATATGTCCTTGATGTTGACAGAGCTCTAAGTGAGCAGACATATCTCCGCTCTCTTGTTATTCCACCTCACCTGAAACAAAGCAATAATTCTTATGGGACTTCATTGCCTGGTGTCCATCTTGATGAAACAATGATACTGAAGGAGAGAAATCGGTTCCCAGAGGTCATCGACAATCTCAAGCTAATTCAGTCGGTATCTGGGATTCAGCAATCTACAGATCTGCTTAACGACAAAAACTATAGAAGTAACAGTTGTGGTATCCAAAACGCAGGTACTAGTGCTGGCCAACAGGTTTGCTTATCTTCACAAAGTTGCCTTACAGCAAATAGTACTTCATATGATGTTAAACTTTCAGCTGAGAATACTCGAGTTAGATTCAAGCATGCCCCCACAGACTACCTGAAGTCTTCTGCTTGGAATACTTCTGTATTAAATGGTGAAGATTGCGCTTCTGAACTAGAACCTGACAAATTTGTGCCTTCAAATCCCCTGGAAAAACTGAAGTCAGAACAAAATGCAGATTTTCTGCTACAATTCAATTGCCTGGAGGCTGGAGATGAGTTGAAGTCATCCTTCGTGTTCTCCGCAGGCAGTGAGTTGCACGAAGTTTTTGGGCCAGCTTTTGTAAAGAGCTGCCATTTGTCTTGGGAGGCAGATAGGCTTGAAGACTGTTCTGCTTCTCAAATGCCAGCGATTATGGAGAGGAGTCTACTTACTTCAGATTCTAGCTCGGATAATCTTCTAGAAGCCGTGGTAGCTAACTTTTGTCGTAAGGATGTCACAGTTAAGAGCGCAATGTCATTTTCTACATCTGAATCTATGTTGACAACAGAAAAAATGCCTGAGCCTTCTATTGATACAAACCCTACAATTGGTTCATCCTGTTACTCTATTGGCTGCTCCTCAATTCTGGAAGGGAGCATGCAAAGCTGCTTGAATTCTTCGGATACCTGTAGCATTAGATCATCAGCTAGTGCTGGCAGGAACACATTTGTGAGGCCAATTGAACCAGGTAAGACTAGCAAGAAGAGGTCAAGACCTGGCGAGAATCCTCGCCCTCGGCCAAGAGATAGGCAGCTGATCCAAGATCGCATCAAAGAGCTCCGAGAACTTGTGCCCAATGGATCAAAGTGTAGCATTGACTCATTACTAGAGCGGACGATCAAGCACATGGTTTTCATGCAAAGCGTCACTAAACATGCTGACAAGCTCAGTAGCTCTGCAAAATCAAGGGGTTGCTGTAAGGAAACAGTAGGATCTGGTTGTGAGCAAGGCTCAAGCTGGGCAGTGGAGGTCGGAGGCCATATGAAAATATGCCCAATTGTGGTGGAGAATCTCGGTGTGAATGGGCAAATGCTAATAGAGATGCTATGTGAAGATTGTGACCATTTTCTGGAGATTACAGAAGCTATCAAGGGCTTGGGCCTAACTGTTCTTAAAGGCATGACAGATAATCATGGAGACAAAACATGGATGCGCTTTGTGGTCGAGGGTCAGAGCAACGTAAACTTGCATAGGATGGATGTGTTGTGGTCGCTTGTCCAGATACTGCAATCAAAAGCCACTGCCTGATGGCCTACTACTTTGACAGCATATTTTGACATCGCTGTGTGCAAAGTCGTGTGAGTTGTACTATGTTTTATTGGCCGAAGCTTGTCTGACTTAGGTTTTCCTTTGCAATGTTCCTTTTTCTTGATAGCCACTTTGTCAAAAGGTTGTGTTTTTTCATTTTGGGCTTGCATCCTCCCTTCAAGTTTAAGATAACAGCTTGATGGGAAGAAACAATGATGATCCAAGGAATCAGTCTTTTTCATCTCTCTGTTGAGAACTTTTGTGGGCCTTTTGATCTTGAAATGGCTGGTAATTGATC

*comp25650_c0*

GGCAACCTTACCTCTACCGGTATTTCTACCCATAGAGAGGTTGATAATACAGTATAGTAGCGTTACAGAATTAAAGCCTGGAAAAGCTATGTTGATGCCTTGAAATTTAATTTCTTTTTTAACATGGGTAGAAATTCTTATTCTTGGACTCTTGTCTAGAAGGCCTATTGAACTGTTTCAAATGCGAGAGGGTGAAGGTGGGACAAGCCCCACAAAGCCTAGGGCGAAAGGGCCTAGTTTGCACAACCCACACAAATATACTTACAAAAGACTTTGTGCGGACCAAGAATTTAACTTAGGTCTCCAACTTGAAATGCGTCTTGTTCTTTACTAAGTTTGAGTTTTTTGTCAAAGTTGTTGAACTTGATTGTAGGTGAACACTAGAGGGGACTGAAATTGGGAAGGAGAAGGTTATATGGGCTATAATCTAAAGGCGACTTACTGTGACTTGACCAATTTGGTGGAGGGAGGGATAGCCACAGTTTCTGCCATTTTTGGAGAACATACCTCAGAAGGGCCGAAGACAAAGGGGGCCAACGTTGTGGATTTGAATCTTGGAAGCCTTGGTGACTTTGGGGAAGGTCGGATAAGCAGCTTCAGGAATCACAAAGCTCCTCTTATGGAGCCATTGTCAGCAGGATCATCAAAAAGGGCTCGTGCACCAAGCAGTGGAGGCCAGACAGTTTCGTGTTTGGTTGATGGGTGCAAGTCGGACCTCAGCAAGTGCCGTGACTACCACCGACGCCATAAAGTTTGTGAAATGCACTCGAAGGCCCCAAGGGTGACAATCGGGGGTCATGAACAACGCTTCTGTCAGCAGTGCAGCAGGTTCCATTCACTGGGAGAGTTTGACGAGGGAAAACGGAGTTGTAGGAAGCGTCTTGAGGGGCACAATCGGCGTAGAAGGAAGCCTCAGCCTGAACCCCTGCCTGTGAATCACGGAAACTTCCTTTCTGCTAGCCAAGGTATGCGTTGACGTTTGATAATTACTTTTTCTTAGCATATTTGAGCTATTCCGTTCTCCTTTGGGACACAAAGTTTGCCTCTTTTTTGTTGCTTGGACTCTGGTACAAGTGTACCACATCGTATGAATCACATGGCGGATGCGCTAATTTTCTTTGACGTTTCACGTCTTTGGGGCTAAAATGCTAATTGTCTATGCTCGTATTAGAGGGTCAAGTGCTCAATATGGGCACTAGAGAAGCGAAGACAAGTAGAGTAAGATAGCTCCTTACTGTGAGCATTTAGCAAGAGAAGCCTATTAAATTTCAATATACTAATTGACAGAAATTGATATCTATTGCAATTATTGGAACTTTTTAGGTGATAGATATATGTCATTTAGCAATCAGCCAGTGAGTTCAGTGGTGAGCACTACTTGGTCTGGACCTGTAAAAGCTGAGAGCAATCCAGCCCGGTTCAGCAGTTCGGTATCTGGTTCTTATTCTCAAGCATACAGAGGCAGGCAGTTCCCCTTTTTGCAAGGCACTGATTCCCCTCTTCCTGGAGCTTCTTCAGTCGGGCAGAGACTTCACCACGATCACAGTTCTTCAGGGAGCAGCAGTAGCAGGAATGTCAACAACATCAATCACGGGCTGAACCAAGTGATCGATTCAGATCATCGTGCTCTCTCTCTTCTGTCATCATCGCCTGCTGAGACTCCCGAGATGGGATTGGGCCGCATAATCCAGCCCAGTCCCATCAACCCAGCCCGACCCATCATCAACAACCTGTTTTACAACAGCATTGGGCAGTTTCCGGGCTCCCAGGGCATGGAAGGACAGCCTGGAGGTTCAGGGTCTGCATTAATCCCCAACATGAGGAGCAGCAACACCCTCTGTCAGGATGTTTTCCGAAGTGAGCCTGATGAATCTTCTGCAAGTGGTCATCAAACACTCTCCTTTTCCTGGGAGTAGCTTTTCAGTTTGTGTTTAATTTTCCTAGCTTAGTGTCCCCCCCCCCCCCCC

*comp27464_c0*

AAAGAAAAGAAAAGGCCAAAGCAGGAGAGAGAAAACAAAACCAAGCCCATCAAAGTCTGGAAAGAGAAAGAAAAAGAGAGTAGGGACGACGAACAAACGACGAGCAACACCACAACAACAACAGCAGAAAGAAGAAGAACTCGAGGGATTTTGCCGCGTAAAGTCGGCACAATCTTCTCTCAAATCCGCCAGTTTCGTACCTTTGGTGTCTCTCACTTGTTCGTCGATCTGGAAGAGTCTAGAGAAAGAGAGAGAGATTGAGTGAGTGAGGTTGAAATTGATTAGTTTGGAGGGAGAAATCTCTGCGATCGAGCAACAATGTTGTTGCTGCAGAGTGATCCACGGCAGTACCAGCTGCAGCAGCAGAACCTGCAAGAACAACAACAGCAGCAACAACAGCAGTTTCAGATTCAGCAGCAATTGCAATTGGCTAGGGTTTCGTTTGTACTCGATCGGAATGACTCGTTCGTTTTCCATTCTGAGCATCAGCAACAAGCGCCGATTCCATCTTCTACATCTATTGTAAAGGCTTCTAAGCCATTGGACCCTCGCCAAGTTGATGACAATCTGTTGTTAAGTCTTGCCCATCAAAAGTACAAAGCTGGTAACTACAAACAAGCACTTGAGCTCAGCAGTACTTTGTATGAGAGAAATCCCTCCCGTACTGATAATCTTCTATTATTGGGAGCCATTTACTATCAGTTGCATGATTTTGATTCTTGCATCTCAAAAAATGAAGAAGCTATTCAAATTGATCCTCATTTTGCTGAGTGTTATGGAAACATGGCAAATGCATGGAAGGAGAAAGGAAATATTGATCTTGCCGTCCGTTACTACTTGTTTGCAATTGAGCTTCGGCCCAACTTTGCTGATGCATGGTCCAATTTGGCCAATGCATACATGCGGAAAGGGAGGCATACTGAGGCAGCTCAATGCTGCCGGCAGGCTCTTGCACTGAATCCTCACCTGGTTGATGCCCGTTGTACCCTGGGTAATTTAATGAAATCTCAAGGCCTAGTGCAGGAGGCATACAACTGTTATGTCGAGGCTCTTCGCATCCAACCTACTCTTTCAATTGCTTGGTCTAATCTTGCTGGTCTTTTTATGGAGACGGGAGATTATAATAATGCTCTTCAATGCTACAAGGAAGCAATCAAGCACAAACCCACTTGCACGGAGGCCTATTTGAACTTAGGAAATGTTTACAAGGCTCTGGGAATGCCTCAAGAAGCTGTCTTATGTTATCAACGTGCTCTGCAGGTCCGACCAGACTATGCTATAGCTTATGCAAGCTTGGCTAGTATATACTATGAGCAAGGACAGCTGGATATGGCAATCCTACATTATGAGAGAGCCATTTCTCGTGATCCCGCATTCTTGGAAGCATACAATAACTTGGGAAATGCACTGAAAGATGCTGGCCGAGTTGATGAAGCAATGCAGTATTATCGTCAATGTCTTTCTCTCCAACCTAGCCACCCCCAAGCATTAACTAATCTTGGGAACATATATATGGAATGGAATATGATTGGTACTGCTGCACAGTATTACAAGGCCACGCTTAATGTTACTACCGGACTATCTGCACCTTACAATAACCTGGCAATTATTTACAAACAGCAGGGTAATTATGCAGATGCTATATCTTGCTACACAGAAGTCCTTAGGATTGATCCATTGGCAGCCGATGCTTATGTCAATAGGGGGAATACATATAAGGAACTTGGAAGAGTGAATGAAGCTATACAAGAATACACAAATGCTATCAATGCCCGCCCTACAATGGCTGAAGCTCATGCAAATCTTGCTTCTGCCTATAAAGACAGTGGCCATGTCGAAGCAGCTATAAAAAGCTACAAGGATGCATTGCTGTTGCGCCCTGACTTCCCAGAGGCAACATGTAATCTTCTTCACACTTTACAGTGTGTCTGTGAGTGGGAGGATAGAGATAGCAGGCTTATTGAAGTGGAGCGCGTACTGAGGCGGCAAATACAGATGTCTGTTCTACCAAGTGTGCAACCATTCCATGCTTTTGCCTATCCTCTTGATCCAATGCTTGCACTTGAAATTAGTCGCAAATACGCTGAGCACTGTTCTATGGTTGCTTCCCGATATTCACTTCCTCCCTTCAATTATCCTACTGCAATACCTGTGAAGGAAGATGGTGGGAGTGGGCGATTGAGAGTAGGGTATGTTAGCAGTGATTTTGGTAACCATCCGCTATCACATCTTATGGGTTCAGTCTGGGGCATGCACAATAGAGAAAATATTGAGGTGTTTTGTTATGCTTTGACTCCAAGTGATGGCTCAGAGTGGAGGTTGCGGACTCAGTCAGAAGCTGAACATTTCAAGGATGTCTCGTCCATGTCCTCTGACGCCATTGCAAGACTGATTAATGAGGATAAGATTCAGATTCTCATCAACCTTAATGGGTATACAAAGGGTGCAAGAAATGAGATATTTGCTATGCAGCCTGCACCGATTCAAGTCTCCTACATGGGATTTCCTGGAACCATGGGTGCCAGTTACATACATTATCTGGTGACTGATGAGTTTGTCTCTCCTCGACGTCTGTCACACATTTATTCAGAGAACCTGGTTCATGTTCCCCACTGCTACTTTGTTAATGACTATAAGCAGAAAAATCTTGATGTGCTGGATCCAAACTGCCAACCAAAGAGATCGGATTATGGACTACCAGAGGACAAGTTCATCTTTGGGTGCTTTAATCAGCTGTATAAAATTGATCCTGATATTTTTGATGCCTGGTGCAATATTCTTAAACGTGTTCCCAACAGTGCTCTCTGGCTGTTGAGATTTCCAGCTGCGGGTGAGAGTAGGCTTCGTGCATATGCTGCTGCAAGGGGTGTGCAACCAGACCAGATTATCTTTACGGATGTTGCAATGAAAGGCGAGCACATTAGGCGCAGTTCTCTGGCTGATCTGTGCCTTGACACGCCATTATGCAACGGACATACAACTGGCACAGATGTTCTGTGGGCTGGTTTGCCAATGTTGACTCTTTCTCTTGAGAAGATGGCCAGCAGGGTTGCTGGTTCTCTGTGTCTGGCAACAGGTGTCGGAGAGGAGATGATTGTGAATAGCCTGAAAGAATATGAAGAGAGGGCAGTGTCTTTGGCACTGAATAGGTCAAAGCTTCGTGATCTTACCAATCGGCTGAAGGCTGCGCGCTTGACTTGTCCTCTGTTTGACACTGCTCGTTGGGTGAGAAACCTCGAGCGAGCCTATTTCAAGATGTGGAATTTATACTGTGTGGGTCAGCATCCTAAGCCATTCACCGTCACCGAGAACGATTTAGAATGCCCTTATGACAGATGAAAAATACAGCCTTGTAGATAGCCTCATATGCTTAGGTTTAATTTAGGGAGTAGGGGGGTGGGGGGAAGAAAGAGGAGTGATTGGTCATGCTGATACTTATTGTGTTAATAGGGTATTAATTGCTGGAGTTTTCAGGACTTGAAAAACTCAGGTAAATCTTTTGCTTTACAACTGATGGGCATTTGGGGATGTTGGGAGGGAAAGAGTTCTGTTTTGTGTTTCAATTCTGTTTGTCACCTTCACTGATGGGCTTTCTTTTGGTCTGTCTCTCTTCGATTGAGGAACAATTATGGTGTCGTTGATTTGCAACGAAGAAAACCAGCTGTTTCATCCTCCCACAATGGTCGAAGCTGGCATAGAGGGGT

*comp6695_c0*

CTCTATAGATCTCTCTCTCTCCAACACATACAAGAGATGGGCAGATCACCGTGCTGTGAGAAAGAACACACAAACAAAGGGGCATGGACCAAAGAAGAAGATGAGAGACTCATCAATTACATCAAGGCTCATGGTGAAGGTTGTTGGAGGTCTCTTCCTAAAGCAGCTGGTTTGCAAAGATGTGGCAAAAGTTGCAGATTGCGATGGATAAACTATCTAAGACCAGGGCTTAAGAGGGGCAATTTCACTGAAGAAGAAGATGAACTCATCATCAACCTCCACAGCTTGCTAGGAAATAAATGGTCTCTCATTGCTGCTCGTTTGCCAGGAAGAACAGATAATGAGATAAAGAACTATTGGAACACCCACATTAAAAGAAAGCTTCTCAGTCGTGGGATAGACCCACGAACTCACCGTCCTCTTAATTCCACCTCCACAACACCCAAAACAACCTCAAGTACAAAGAGTGCGACTCATAACAACAACAACAACATGAATACATTCAAGTTGTTCAACGTACAAGGTGGCTCAACCTTACAATTCAGCATGGTATCAGAGTTAGATCTCACAACAAGGAGCAATTGCAATGATAGTTACAATGCCCGGGGTACTAGAGCATCGAGTATTAGTGAAGAATCTAACAGTAGTAGCATAGTGACTGGGGAGGAAGCTTATGGAGAAATTAATCTCGAATTATCTATGAGTCTTCCTAGCTCTGATTTGAAGATGTCCACAAACGAAATCAAGAAGGAACAACAAGCAGTAGAATACCAAAATTTAATGAGTTGTATGCCAGTGTCCACAAATGCAACAACTATAGTTGCCAATGATTTCCATGGATTTTCCAAACCTCATAGTTTATCATGAGGATATATACATTATAGTTAGTGTTATAAATTCAGTTAC

‘NN’ indicates sequence of target genes primers for real-time PCR. ‘NN’ indicates outer specific primers for nested PCR. ‘NN’ indicates inner specific primers for nested PCR.
